# Lysophosphatidic acid reverses Temsirolimus-induced changes in lipid droplets and mitochondrial networks in renal cancer cells

**DOI:** 10.1371/journal.pone.0233887

**Published:** 2020-06-03

**Authors:** Ravneet Chhabra, Meera Nanjundan

**Affiliations:** Department of Cell Biology, Microbiology, and Molecular Biology, University of South Florida, Tampa, Florida, United States of America; Faculty of Medicine, University of Belgrade, SERBIA

## Abstract

Increased cytoplasmic lipid droplets (LDs) and elevated AKT/mTOR signaling are characteristics of clear cell renal cell carcinoma (ccRCC). Lysophosphatidic acid (LPA), a potent lipid mitogen generated via autotaxin (elevated in ccRCC), can modulate tumor progression but its role in altering chemotherapeutic sensitivity to mTOR inhibitors is unclear and thus is the focus of the studies presented herein. Using malignant (A-498, 769-P and 786-O) and normal immortalized kidney (HK-2) cell lines, we investigated their cellular responsiveness to Temsirolimus (TEMS, mTOR inhibitor) in the absence or presence of LPA by monitoring alterations in AKT/mTOR pathway mediators (via western blotting), LDs (using LipidTOX and real-time PCR to assess transcript changes in modulators of LD biogenesis/turnover), mitochondrial networks (via immunofluorescence staining for TOM20 and TOM70), as well as cellular viability. We identified that TEMS reduced cellular viability in all renal cell lines, with increased sensitivity in the presence of an autophagy inhibitor. TEMS also altered activation of AKT/mTOR pathway mediators, abundance of LDs, and fragmentation of mitochondrial networks. We observed that these effects were antagonized by LPA. In HK-2 cells, LPA markedly increased LD size and abundance, coinciding with phospho-MAPK and phospho-S6 activation, increased diacylglycerol O-acetyltransferase 2 (DGAT2) mRNA (which produces triacylglycerides), and survival. Inhibiting MAPK partially antagonized LPA-induced LD changes. Collectively, we have identified that LPA can reverse the effects of TEMS by increasing LDs in a MAPK-dependent manner; these results suggest that LPA may contribute to the pathogenesis and chemotherapeutic resistance of ccRCC.

## Introduction

Renal cell cancer (RCC) is one of the most common urological malignancies. Contributing factors to disease pathogenesis include smoking, obesity, as well as mutations in Von Hippel-Lindau (VHL) [1]. Of the five major subtypes of RCC, clear cell RCC (ccRCC) is the most common and lethal subtype; it is a metabolic disease characterized by dysregulated lipid metabolism, altered gene regulation due to multiple genomic aberrations, and increased abundance of lipid droplets (LDs) [1–3]. Regrettably, the overall patient survival rate is <15% for advanced RCC disease [1] and thus an improved understanding of the underlying mechanisms of RCC pathogenesis is direly needed to develop improved treatment regimens. There currently exists several first-line targeted therapies which are FDA approved for ccRCC, including mTOR targeting agents [1]. The PI3K/AKT/mTOR pathway is highly dysregulated in ccRCC [4]; targeting mTOR (which modulates cellular survival, blood vessel development, and nutrients) with rapamycin can modulate LD formation [5]. Specifically, mTORC1 can regulate the lipogenesis and lipolysis pathways via peroxisome proliferator-activated receptor gamma (PPAR-γ) and sterol regulatory element-binding protein 1 (SREBP1) [4, 5]. Notably, LDs can physically associate with mitochondria at defined contact sites; these organellar interactions promote cellular protection from stress via the process of β-oxidation (the breakdown of fatty acids to acetyl-CoA, which can then be utilized in the citric acid cycle to generate cellular energy) [6]. However, the role of mTOR clinical targeting agents (including Rapalogs such as Temsirolimus (TEMS) [7]) in the regulation of mitochondrial networks and LD biogenesis has not yet been investigated in ccRCC.

mTOR inhibitors are associated with low clinical efficacy and this may be due to the activation of the cytoprotective autophagic pathway (a “self-eating” mechanism [8]) which may then antagonize the cell death promoting effects of such inhibitors. Indeed, improvements to cellular sensitivity to mTOR inhibitors has been demonstrated by co-targeting of the autophagic pathway [9]. In a phase I clinical trial combining TEMS with hydroxychloroquine (HCQ), there was improved clinical response in melanoma patients [9, 10]. Another potential contributor to diminished cellular sensitivity to mTOR inhibitors may include the presence of the potent lipid mitogen, lysophosphatidic acid (LPA), which activates G-protein coupled receptors to increase cellular proliferation, migration, and invasive potential via activation of the AKT pathway [11, 12]. This mitogen is produced via the action of autotaxin (ATX), a member of the endonucleotide pyrophosphatase and phosphodiesterase family of enzymes (ENPP2), which elicits lysophospholipase D (lysoPLD) activity (which hydrolyses lysophosphatidylcholine (LPC) to generate LPA [11, 12]. Interestingly, ATX mRNA and protein in addition to its lysoPLD activity are elevated in RCC (relative to normal epithelium) [13–15]. Furthermore, the LPA-ATX axis can contribute to resistance against sunitinib in RCC pathogenesis [14]. Although a derivative of LPA (phosphatidic acid, PA) has been shown to contribute to LD enlargement by promoting their fusion [16], to the best of our knowledge, it remains unclear whether LPA can modulate lipid droplet abundance, a key characteristic of ccRCC, in renal cancer cells.

Herein, we have analyzed the effect of TEMS in a series of ccRCC cell lines (769-P, 786-O, and A-498) together with an immortalized normal human kidney cell line (HK-2) to identify alterations in signaling, lipid droplet formation, and mitochondrial networks following treatment with TEMS alone. We also assessed whether combinatorial treatment of TEMS with the autophagic inhibitor, hydroxychloroquine (HCQ) could modulate cellular viability and lipid droplet abundance. Finally, we investigated whether the presence of LPA could hinder the effect of TEMS treatment in the ccRCC cell lines in terms of lipid droplet abundance and AKT/mTOR signaling. Collectively, our results identify that the LPA-ATX signaling axis may be an important target for combating the resistance acquired by RCC cells towards molecular-based therapies.

## Materials and methods

### Cell culture

Human epithelial renal cancer cell lines (769-P, 786-O, and A-498) and an immortalized kidney proximal tubule cell line (HK-2) [17] were purchased from ATCC (Manassas, VA). HK-2 cells were cultured in Keratinocyte-serum free media (K-SFM, #17-005-042, Fisher Scientific, Pittsburgh, PA) supplemented with 50 μg/ml bovine pituitary extract (BPE, #10450–013) and 5 ng/ml human recombinant epidermal growth factor (EGF, #13028–014). 769-P and 786-O cells were grown in RPMI 1640 media containing 8% fetal bovine serum (FBS) and 1% penicillin/streptomycin whereas A-498 cells were cultured in EMEM media supplemented with 8% FBS and 1% penicillin/streptomycin. All cell lines used in this manuscript were authenticated by Short Tandem Repeat (STR) profiling (Genetica DNA Laboratories, Cincinnati, OH) and were tested to be mycoplasma negative. For the experiments presented in this manuscript, the above cell lines were utilized at the following passage numbers: (1) 769-P cells, p = 41–54; (2) 786-O cells, p = 113–127; (3) A-498 cells, p = 38–53; and (4) HK-2 cells, p = n+4 –n+20.

### Cell treatments

A 10 mM stock of the mTOR inhibitor, Temsirolimus (TEMS, #50-811-7, Fisher Scientific, Pittsburgh, PA) was prepared by reconstitution in dimethyl sulfoxide (DMSO) and used at a final concentration of 10 μM. Oleic acid (#O1383, Sigma-Aldrich, St. Louis, MO) was reconstituted in 100% ethanol to prepare a 0.35 M solution, which was utilized at a final concentration of 0.25 mM. The autophagic flux inhibitor, hydroxychloroquine (HCQ, AC263011000, Fisher Scientific, Pittsburgh, PA), was prepared in phosphate buffered saline (PBS) at 50 mM and used at a final concentration of 25 μM. The MAPK inhibitor, U0126 (#9903S, Cell Signaling Technology, Danvers, MA), was reconstituted in DMSO at a concentration of 10 mM and utilized at a final concentration of 10 μM.

Fatty acid-free bovine serum albumin (FAF-BSA, BP9704-100, Fisher Scientific, Pittsburgh, PA) was dissolved in PBS to generate a 0.5% stock solution. 1-Oleoyl-2-hydroxy-sn-glycero-3-phosphate (sodium LPA salt in chloroform, #857130C, Avanti Polar Lipids, Alabaster, AL) was air-dried and reconstituted in 0.5% FAF-BSA to generate a stock solution of 3.5 mM and used at a final concentration of 10 μM.

### siRNA transfections

HK-2 cells were seeded at 350,000 cells/well in 6-well plates. After overnight adherence, cells were transfected with the following siRNA using Dharmafect I (#NC1308404, Fisher Scientific, Pittsburgh, PA) according to previously published methods [18, 19]: ribosomal protein S6 (RPS6) siRNA (#L-003024-00-0005), ribosomal protein S6 kinase B1 (p70S6K1) siRNA (#L-003616-00-0005), or non-targeting ON-TARGETplus (D-001810-10-20) siRNA (Dharmacon, Lafayette, CO). The day after transfection and following cell recovery, cells were treated with 10 μM LPA (or with an equivalent volume of 0.5% BSA) for 24 hours followed by protein analyses or immunofluorescence LD staining (as described below).

### Protein isolation and western analyses

Using previously published methodology [18–20], protein lysates were collected and normalized to at least 1,000 μg/ml using the Bicinchoninic Assay (BCA, Fisher Scientific, Pittsburgh, PA). The samples were then run on 10% SDS-PAGE gels and analyzed by western blotting using primary antibodies at the following dilutions: (1) Beclin-1 rabbit polyclonal (#3738, 1:1000), DRP1 rabbit monoclonal (#8570, 1:1000), hVPS34 rabbit monoclonal (#3358, 1:1000), LC3B rabbit polyclonal (#2775, 1:1000), p-AKT (Ser-473) rabbit monoclonal (#4060, 1:1000), p-GSK3 (Ser-21/Ser-9) rabbit polyclonal (#9331, 1:1000), p-S6 ribosomal protein (Ser-235/236) rabbit monoclonal (#4858, 1:1000), Pan-Actin rabbit polyclonal (#4968, 1:500), PARP rabbit polyclonal (#9542, 1:1000), p-DRP1 (Ser-616) rabbit monoclonal (#4494, 1:1000), p-p42/44 MAPK (Thr-202/Tyr-204) rabbit polyclonal (#9101, 1:750), Pan-AKT rabbit monoclonal (#4685, 1:1000), p42/44 MAPK rabbit monoclonal (#4695, 1:1000), p70S6K rabbit monoclonal (#2708, 1:1000), and S6 rabbit monoclonal (#2217, 1:1000) which were obtained from Cell Signaling Technology (Danvers, MA); (2) p62 mouse monoclonal (#610832, 1:1000) was obtained from BD Biosciences (San Jose, CA, USA); (3) Perilipin mouse monoclonal (#SC-390169, 1:500), TOM20 rabbit polyclonal (#SC-11415, 1:7500), TOM40 mouse monoclonal (#SC-365467, 1:1000), and TOM70 mouse monoclonal (#SC-390545, 1:1000) were obtained from Santa Cruz Biotechnology (Dallas, TX, USA); and (4) ATG7 rabbit polyclonal (#PM039, 1:1000) from MBL International Corporation (Woburn, MA, USA).

### RNA isolation and real-time PCR

Total RNA was isolated using the RNeasy Mini kit (QIAGEN, Valencia, CA) according to previously published methods [19, 20]. Real-time PCR utilized the TaqMan RNA-to-C_T_ One-Step Kit (#4392938, ThermoFisher Scientific, Waltham, MA) and the following FAM-labelled probes and primers: (1) Phospholipid Phosphatase 1 (PLPP1), Hs00170356_m1; (2) Diacylglycerol O-acyltransferase 2 (DGAT2), Hs01045913_m1; (3) Perilipin 1 (PLIN1), Hs00160173_m1; (4) Cell death-inducing DFFA-like effector c (CIDEC), Hs00535724_gH; (5) Adipose Triglyceride Lipase (ATGL/PNPLA2) Hs00386101_m1; and (6) Autotaxin (ATX/ENPP2), Hs00905117_m1. Normalization of C_T_ values was performed using β-actin (#401846, ThermoFisher Scientific, Waltham, MA) and RNA-fold changes were calculated using the formula 2^−ΔΔCT^.

### Cell viability assay

Cells were seeded at 7,500 cells (Fig 2B) or at 4,000 cells (Fig 4C) per well in a 96-well plate or at 125,000 cells per well (Fig 5B) in a 6-well plate. After cellular treatment for appropriate time periods, cells were then stained with crystal violet for 15 minutes, extensive washing with nanopure water, and overnight drying. Next, samples were solubilized with 200 μl of Sorenson’s buffer, incubated for 2 hours at room temperature on a rotating platform, and then read at 570 nm with a Biotek plate reader according to previously published methods [18, 21].

### Mitochondrial network staining via immunofluorescence

Immunofluorescence staining methods have been previously described [18, 22]. Briefly, cells were seeded onto glass coverslips at 250,000 cells/well (in 6-well plates). After overnight attachment, cells were treated with 10 μM TEMS or with 0.25 mM oleic acid for 24 hours. Mitochondrial networks were assessed using antibodies targeting TOM20 (1:100) or TOM70 (1:100) followed by incubation using the appropriate fluorophore-conjugated secondary antibodies. Coverslips were mounted using anti-fade solution (containing DAPI) onto glass slides which were viewed and imaged using the 63X objective (oil immersion) on the PerkinElmer UltraVIEW Confocal spinning disc microscope (CMMB Core Facility, University of South Florida, Tampa, Florida). For quantification, the mitochondrial patterns were categorized according to the following five categories: (1) Tubular elongated; (2) Tubular shortened; (3) Tubular shortened fragmented; (4) Fragmented mitochondria; and (5) Fused not elongated [18]. One hundred cells per sample were assessed and cells were assigned to these five categories.

### Lipid droplet (LD) staining using LipidTOX

LD staining was performed as previously reported [18]. Briefly, cells were seeded at 250,000 cells/well onto glass coverslips in 6-well plates. After overnight attachment, cells were treated with 10 μM TEMS or with 0.25 mM oleic acid for 24 hours. LDs were stained using LipidTOX neutral lipid dye. Slides were viewed and imaged at 63X (oil immersion) magnification using the PerkinElmer UltraVIEW Confocal spinning disc microscope (CMMB Core Facility, University of South Florida, Tampa, Florida).

LipidTOX stained immunofluorescence images were analyzed using Image J to quantify LD size (area covered by LD) and number (number of particles). The color threshold of the images analyzed were adjusted by using the Hue, Saturation, Brightness (HSB) color model; the hue and saturation were kept constant at 0 and the brightness using the red threshold color was varied but kept consistent for different treatments with an independent experiment to allow appropriate comparison between them to facilitate an unbiased analyses. LDs within each image were analyzed for the number of LDs as well as the area covered by each droplet (size: 0 to infinity). The total number of LDs and their sizes were then calculated for each image with respect to the total number of cells present (i.e., number of DAPI stained nuclei within the image) to obtain the total area covered by LDs per cell as well as the total number of LDs per cell. The values obtained from treated samples were normalized to the values obtained from the corresponding untreated samples.

### Measurement of cholesterol

Total protein lysates were normalized using the BCA assay as previously described [18]. In these samples, the Amplex Red Cholesterol Assay Kit (#A12216) obtained from Life Technologies (Grand Island, NY) was utilized to assess total cholesterol levels as previously described [18].

### Statistical analyses

All experiments were performed independently at least three times. The error bars shown in all displayed figures represent standard deviations; p-values were obtained using the standard student’s t-test using the GraphPad Prism software (**** = p ≤ 0.0001, *** = p ≤ 0.001, ** = p ≤ 0.01, * = ≤ 0.05, and ns = not significant (p>0.05)).

## Results

### Comparative analyses of normal and a subset of malignant renal cell lines

**Fig 1:**
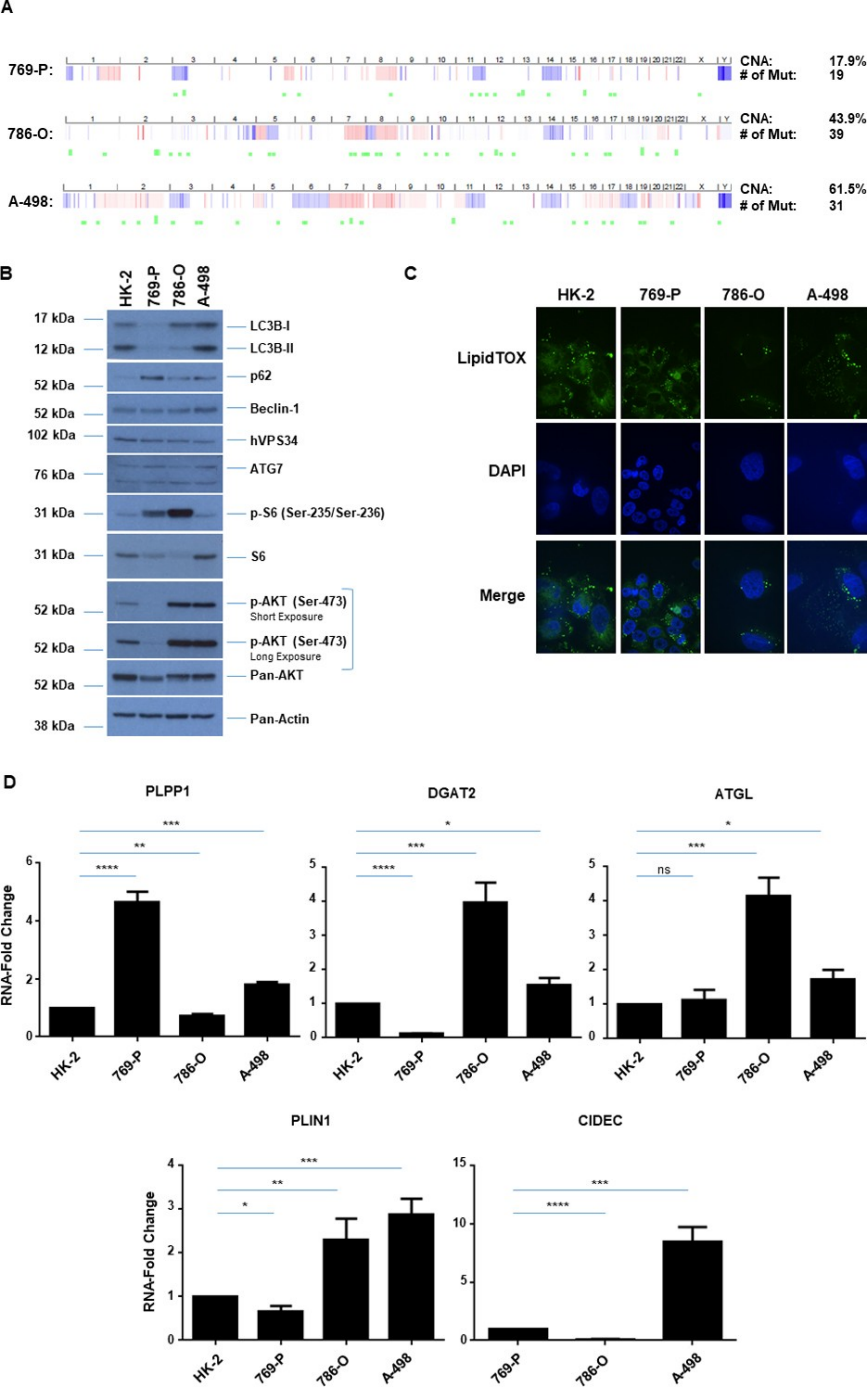
Status of genetic alterations, AKT/mTOR pathway, and LDs across normal and malignant renal cell lines. (A) Copy number alterations (CNA) across three malignant ccRCC cell lines (769-P, 786-O, A-498) and mutational status (Mut) derived from TCGA. (B) Western blot analyses of ccRCC cell lines and HK-2 cells with the indicated antibodies. Three independent experiments were performed and representative blots are displayed. (C) LD assessment of ccRCC cell lines and HK-2 cells via immunofluorescence staining using LipidTOX. Three independent experiments were performed and representative images are displayed. (D) Real-time PCR analyses of genes involved in LD biogenesis and regulation in ccRCC cell lines and HK-2 cells. Data shown is the composite of three independent experiments.

It is well established that genomic aberrations are characteristic of numerous cancer types and such alterations provide insight into patient survival as well as response to chemotherapeutic regimens [23, 24]. In ccRCC, loss of 3p (which harbors the VHL gene) appears to be an obligate event in RCC pathogenesis [24, 25]. The additional loss of chromosome 14q leads to increased aggressiveness of ccRCC and is associated with a poor patient outcome [24, 26]. Apart from these 3p and 14q alterations, gain at 5q (harboring SQSTM1) is another common alteration in ccRCC [24, 27]. To correlate responsiveness of ccRCC cell lines to established forms of treatments with extent of genomic aberrations, we analyzed a subset of malignant clear cell renal cell lines, via The Cancer Genome Atlas (TCGA: [28, 29]). Indeed, TCGA analysis showed that 769-P cells contained the least percentage of genomic alterations at 17.9%, followed by 786-O at 43.9%, and A-498 at 61.5% (Fig 1A). Specifically, the 769-P and A-498 cells were characterized by extensive deletions at both the 3p and 14q loci; however, although the 786-O cell line contained 14q deletions, it was lacking chromosomal losses at the 3p locus.

Since the AKT/mTOR and autophagy pathway has been reported to be dysregulated in a variety of tumors including ccRCC [4, 30, 31] with potential to alter chemotherapeutic cellular response, we assessed the baseline expression and activation status of key signaling mediators in the AKT/mTOR pathway across a subset of malignant renal cancer cell lines. As presented in Fig 1B, the activation status of phospho-S6 was elevated in 769-P and 786-O cells relative to A-498 cells whereas phospho-AKT was markedly elevated in 786-O and A-498 cells relative to 769-P cells. These patterns, however, did not correlate with the 3p and 14q TCGA-defined extent of genomic aberrations. Similarly, no association was uncovered with lipidated LC3B and other key autophagic markers (Fig 1B). Since ccRCC is characterized by the presence of LDs [1, 2] and lipidomic analyses have uncovered alterations within the lipidome [32], we next assessed the quality and abundance of LDs across these cell lines. All cell lines assessed contained LDs as identified by immunofluorescence LipidTOX staining and their quality/abundance did not appear to be correlated with the extent of genomic alteration at 3p and 14q (Fig 1C). Furthermore, the RNA levels (via real-time PCR) of key mediators involved in LD formation/turnover such as phospholipid phosphatase 1 (PLPP1, which converts PA to DAG), diacylglycerol acyltransferase 2 (DGAT2, which converts DAG to TAG), adipose triglyceride lipase (ATGL, which regulates lipolysis), and cell death inducing activator like effector C (CIDEC, involved in LD enlargement) were more elevated in the A-498 cell line (relative to the normal immortalized HK-2), a cell line associated with increased genomic aberrations. All malignant renal cell lines had detectable expression of these LD markers with the exception of CIDEC in HK-2 cells (Fig 1D).

### TEMS reduces cellular viability in normal and malignant renal cell lines

**Fig 2:**
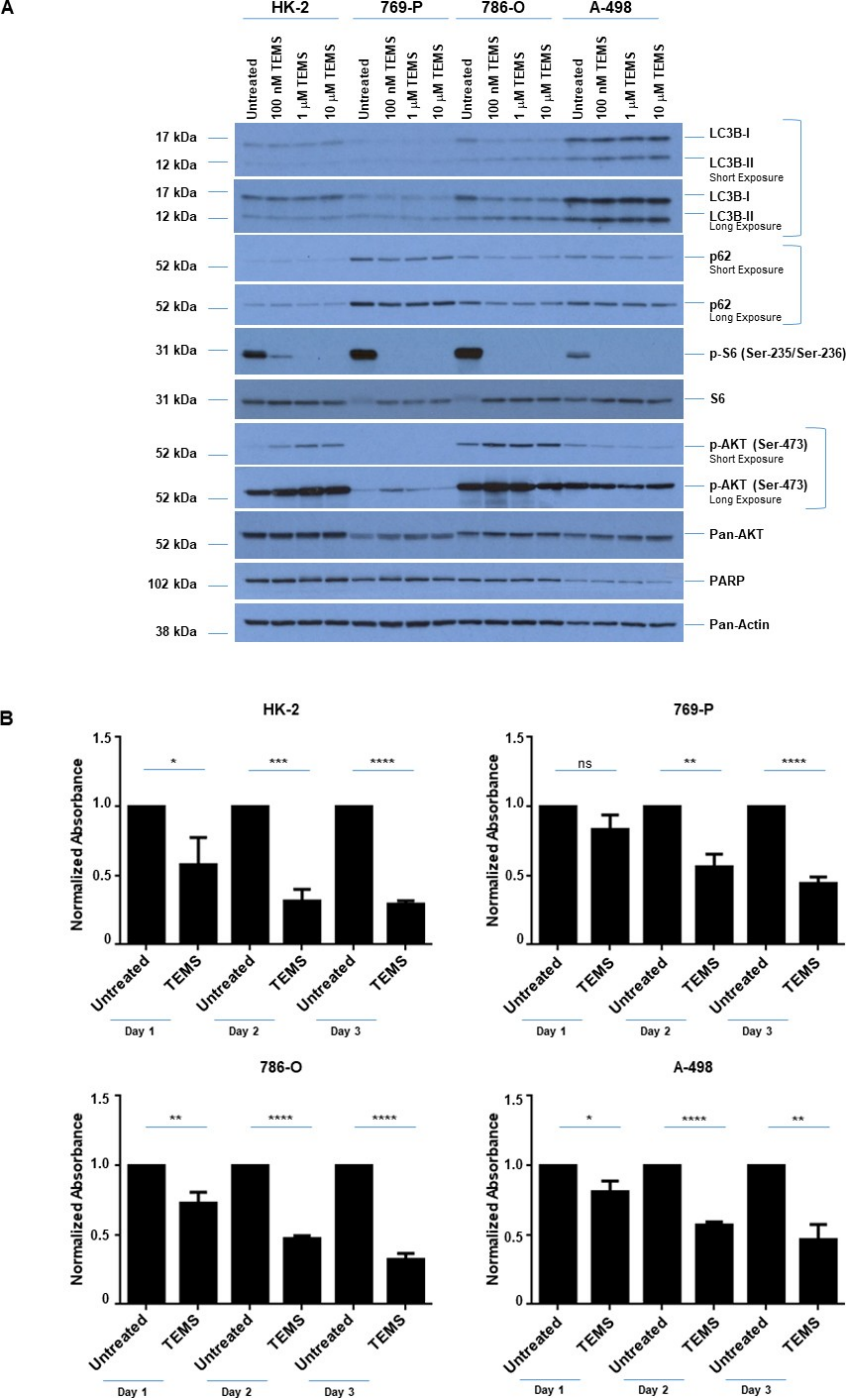
TEMS alters activation status of AKT/mTOR, expression of autophagic mediators, and reduces cellular viability in normal and malignant renal cell lines. (A) Western blot analyses of ccRCC cell lines and HK-2 cells treated with increasing doses of TEMS (100 nM– 10 μM) with the indicated antibodies. Three independent experiments were performed and representative blots are displayed. (B) Assessment of cellular viability in ccRCC cell lines and HK-2 cells at Day 1–3 in response to 10 μM TEMS. The composite of three independent experiments is displayed.

Since targeting the mTOR pathway is a part of the chemotherapy regimen for advanced kidney cancer patients [4], we next assessed whether the responsiveness to this agent was correlated with the extent of genomic aberrations at 3p and at 14q. Thus, we first tested a variety of doses of the mTOR inhibitor (100 nM to 10 μM of TEMS) in 769-P, 786-O, A-498 malignant renal cancer cells as well as the normal immortalized HK-2 cell line. Cell lysates were collected following a 24 hour treatment period and analyzed via western blotting for mTOR pathway mediators as well as autophagy markers. As shown in Fig 2A, phospho-S6 was markedly reduced with TEMS treatment across all four cell lines, which validated mTORC1 inhibition. Phospho-AKT (at Ser-473) increased in the 769-P and 786-O cells as well as the HK-2 cells with increasing doses of TEMS whereas in the A-498 cells, the activation status of AKT decreased. With respect to the autophagy pathway, both lipidated LC3B and p62 were subtly reduced with increasing doses of TEMS in HK-2 as well as 786-O and A-498 cells. Since we noted that the TEMS-induced cellular changes were most striking at 10 μM, this dose was selected to assess changes in cellular viability across these four renal cell lines. We noted that all cell lines were susceptible to growth inhibition in response to TEMS up to 3 days of treatment (Fig 2B). Specifically, the cell numbers of HK-2, 786-O, 769-P, and A-498 were reduced by 42.6%/70.5%, 26.9%/67.7%, 16.8%/55.5%, and 19.3%/53.4%, respectively on day 1/day 3 of treatment, respectively, although this was not accompanied by alterations in PARP cleavage (a marker of caspase-dependent apoptosis) (Fig 2A).

### TEMS alters LD abundance and mitochondrial networks in malignant renal cell lines

**Fig 3:**
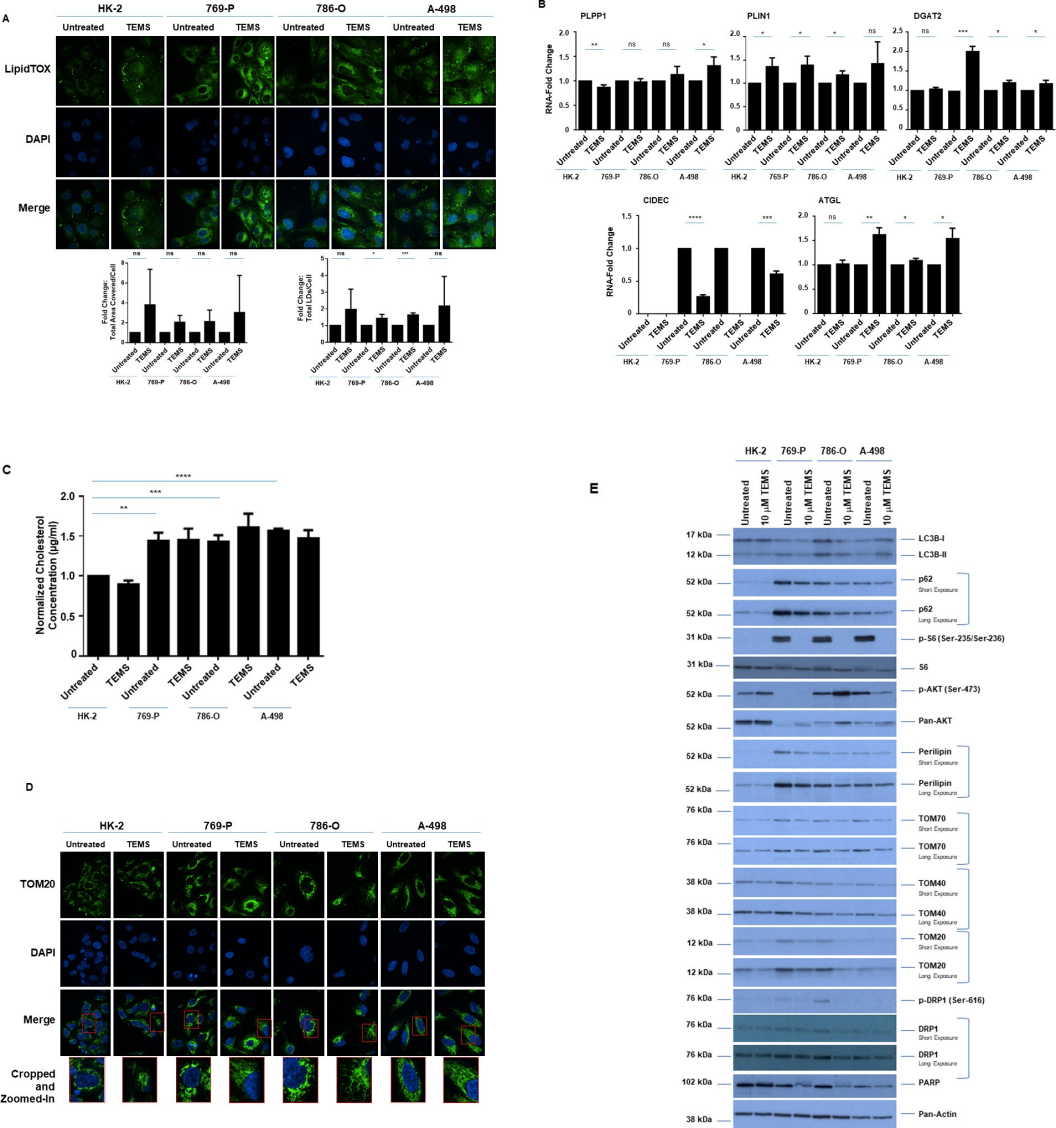
TEMS alters LD abundance and mitochondrial networks in malignant renal cell lines. (A) LD assessment of ccRCC cell lines and HK-2 cells in response to 24 hour treatment with 10 μM TEMS via immunofluorescence staining using LipidTOX. Three independent experiments were performed and representative images are displayed as well as quantification of Total Area Covered/Cell and Total LDs/Cell (presented as normalized Fold-Change). (B) Real-time PCR analyses of genes involved in LD biogenesis and regulation in ccRCC cell lines and HK-2 cells in response to 24 hour treatment with 10 μM TEMS. Data shown is the composite of three independent experiments. (C) Assessment of total cholesterol levels in ccRCC cell lines and HK-2 cells in response to 72 hour treatment with 10 μM TEMS. The composite of three independent experiments is displayed. (D) Mitochondrial network assessment of ccRCC cell lines and HK-2 cells in response to 24 hour treatment with 10 μM TEMS via immunofluorescence staining of TOM20. Three independent experiments were performed and representative images are displayed. (E) Western blot analyses of ccRCC cell lines and HK-2 cells treated for 72 hours with 10 μM TEMS with the indicated antibodies. Three independent experiments were performed and representative blots are displayed.

Since increased LDs are a characteristic feature of ccRCC [1, 2], we next assessed whether LD size and/or abundance was altered by targeting the mTOR pathway with TEMS. Thus, we treated the three malignant cell lines (769-P, 786-O, and A-498) and the normal immortalized HK-2 cells with TEMS for 24 hours followed by staining with LipidTOX. As shown in Fig 3A, we observed increased LD abundance in 769-P and 786-O cells, smaller more numerous numbers of LDs in A-498 cells, and a slight LD increase in HK-2 cells. Furthermore, the mRNA levels of lipid droplet regulators (PLPP1, CIDEC, DGAT1, ATGL and PLIN1) following 24 hours of TEMS treatment showed significantly marked alterations in their expression (Fig 3B), notably in the malignant renal cell lines. Interestingly, ATGL contributes to the process of lipolysis, which may lead to the increased numbers of LDs following TEMS treatment in A-498 cells.

Since LDs are composed of cholesteryl esters as well as triacylglycerides and since cellular cholesterol is linked with cancer progression [33], we next assessed total cellular cholesterol levels upon 72 hours of TEMS treatment in the three malignant RCC cell lines as well as HK-2 cells. Using the Amplex Red Assay, we noted that cholesterol was significantly higher in the malignant ccRCC cells (769-P, 786-O, and A-498) relative to the normal immortalized kidney HK-2 cell line (Fig 3C). However, TEMS treatment did not have any effect on cholesterol in any of these cell types. These data suggest that the changes in lipid droplets observed upon TEMS treatment do not alter total cellular cholesterol levels.

Although LDs interact with mitochondria to promote energy production via the β-oxidation pathway [6], the role of TEMS in regulating mitochondrial networks has not yet been investigated in ccRCC. Thus, we analyzed whether mitochondrial networks are affected by 10 μM TEMS following 72 hours treatment in renal cells. We detected mitochondrial alterations via immunofluorescence staining of both TOM20 as well as TOM70 (outer mitochondrial membrane proteins which facilitate the movement of pre-proteins through the TOM40 translocation pore [34]). As shown in Fig 3D, we observed that the fragmented mitochondrial appearance (in control cells) changed to a fused tubular elongated structure following TEMS treatment. To assess whether expression of these mitochondrial proteins was altered, we performed western blot analyses of renal cells treated for 72 hours with 10 μM TEMS. As shown in Fig 3E, we identified that TOM20, TOM40, and TOM70 proteins were decreased following TEMS treatment in the malignant renal cancer cell lines (most notably in 786-O cells) in addition to HK-2 cells. Interestingly, these changes coincided with decreased protein expression of perilipin, p62, and the DRP1 (Dynamin-related protein1) mitochondrial fission protein. Further, marked alterations in the lipidated form of LC3B was noted in the malignant renal cell lines and the total protein levels of the DNA repair mediator PARP were reduced upon TEMS treatment suggesting that mTORC1 inhibition may deregulate autophagy as well as downregulate the DNA repair mechanism.

### Oleic acid alters LD abundance and cellular viability of normal immortalized HK-2 cells

**Fig 4:**
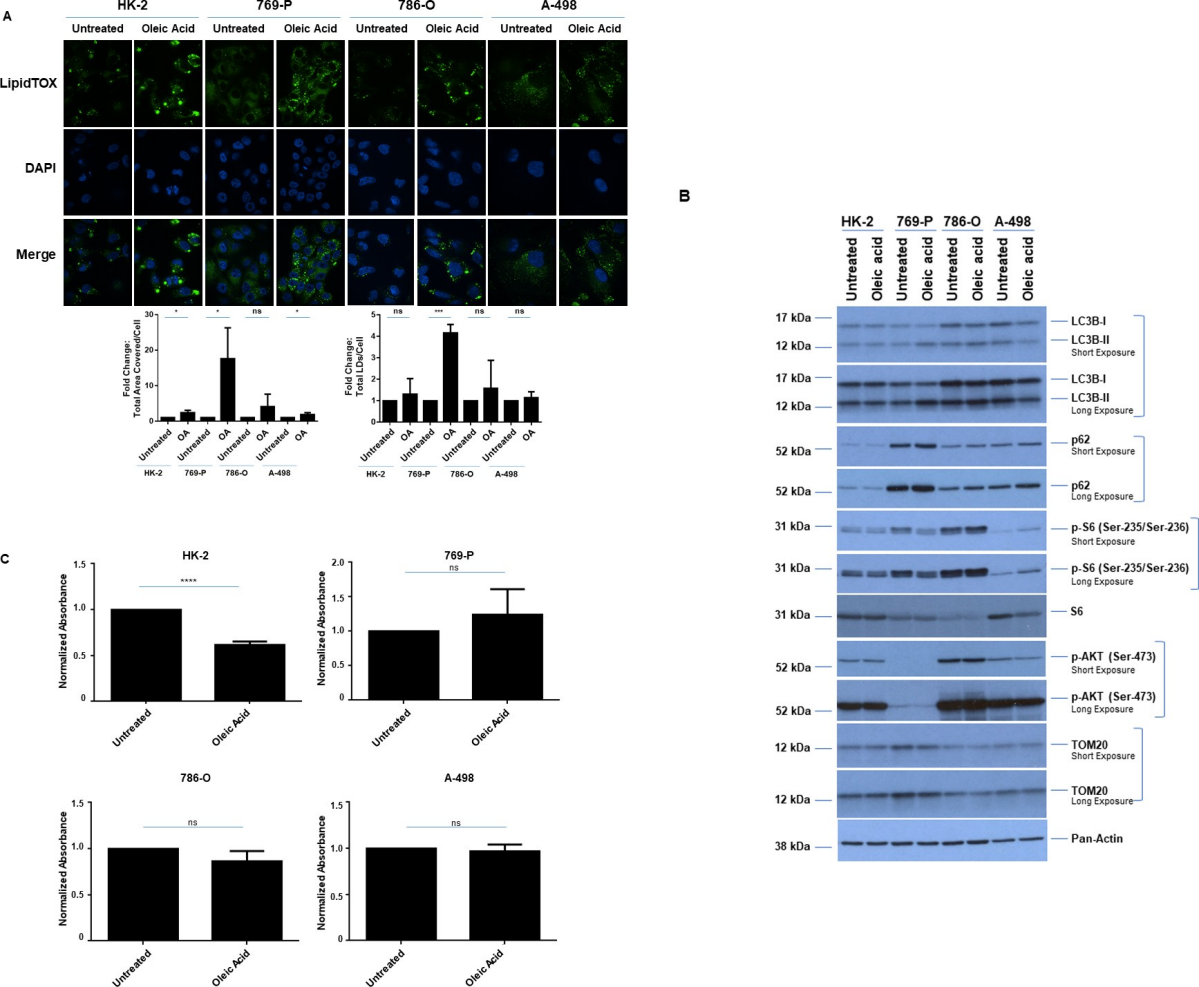
Oleic acid alters LD abundance and cellular viability of normal immortalized HK-2 cells. (A) LD assessment of ccRCC cell lines and HK-2 cells in response to 24 hour treatment with 0.25 mM oleic acid via immunofluorescence staining using LipidTOX. Three independent experiments were performed and representative images are displayed as well as quantification of Total Area Covered/Cell and Total LDs/Cell (presented as normalized Fold-Change). (B) Western blot analyses of ccRCC cell lines and HK-2 cells treated for 72 hours with 0.25 mM oleic acid with the indicated antibodies. Three independent experiments were performed and representative blots are displayed. (C) Assessment of cellular viability in ccRCC cell lines and HK-2 cells at Day 3 in response to 0.25 mM oleic acid. The composite of three independent experiments is displayed.

Lipid metabolic pathways are commonly exploited by cancer cells, which contribute to increased cellular proliferation and survival [35]. Since exogenous lipid supply (i.e., fatty acids) can increase LDs [36] and we noted that TEMS increases LD abundance, we next assessed whether oleic acid could modulate pathways similar to TEMS. Thus, we treated renal cells (three malignant and a normal immortalized HK-2 cell line) with 0.25 mM oleic acid (selected after testing a range of doses from 250 nM to 2.5 mM [37–39]). First, we investigated whether exogenous presentation of oleic acid (for 24 hours) to these cell lines could modulate lipid droplet formation. As shown in Fig 4A, oleic acid treatment increased lipid droplet size in all four cell lines with statistically significant alterations in HK-2, 769-P, and A-498 cells; in 769-P cells, the number of lipid droplets also significantly increased. However, western blot analyses showed variable alterations in phospho-S6 levels and LC3B-II with no change in p62, phospho-AKT, or TOM20 (Fig 4B) across the four cell types treated with oleic acid for 72 hours. These results suggest that the mechanism of LD increase via oleic acid may differ to that mediated by TEMS and across different cell types. Furthermore, we identified that oleic acid could alter HK-2 cell viability (but not the malignant cancer cells) when treated up to three days (Fig 4C). These results suggest that excess fatty acids and possibly LD formation may have a negative effect on the viability of normal cells whereas the renal cancer cells may utilize this monounsaturated fatty acid for essential cellular metabolic processes to support their current proliferative and survival capacity.

### Hydroxychloroquine (HCQ) alters LDs and synergizes with TEMS to reduce cellular viability in renal cell lines

**Fig 5:**
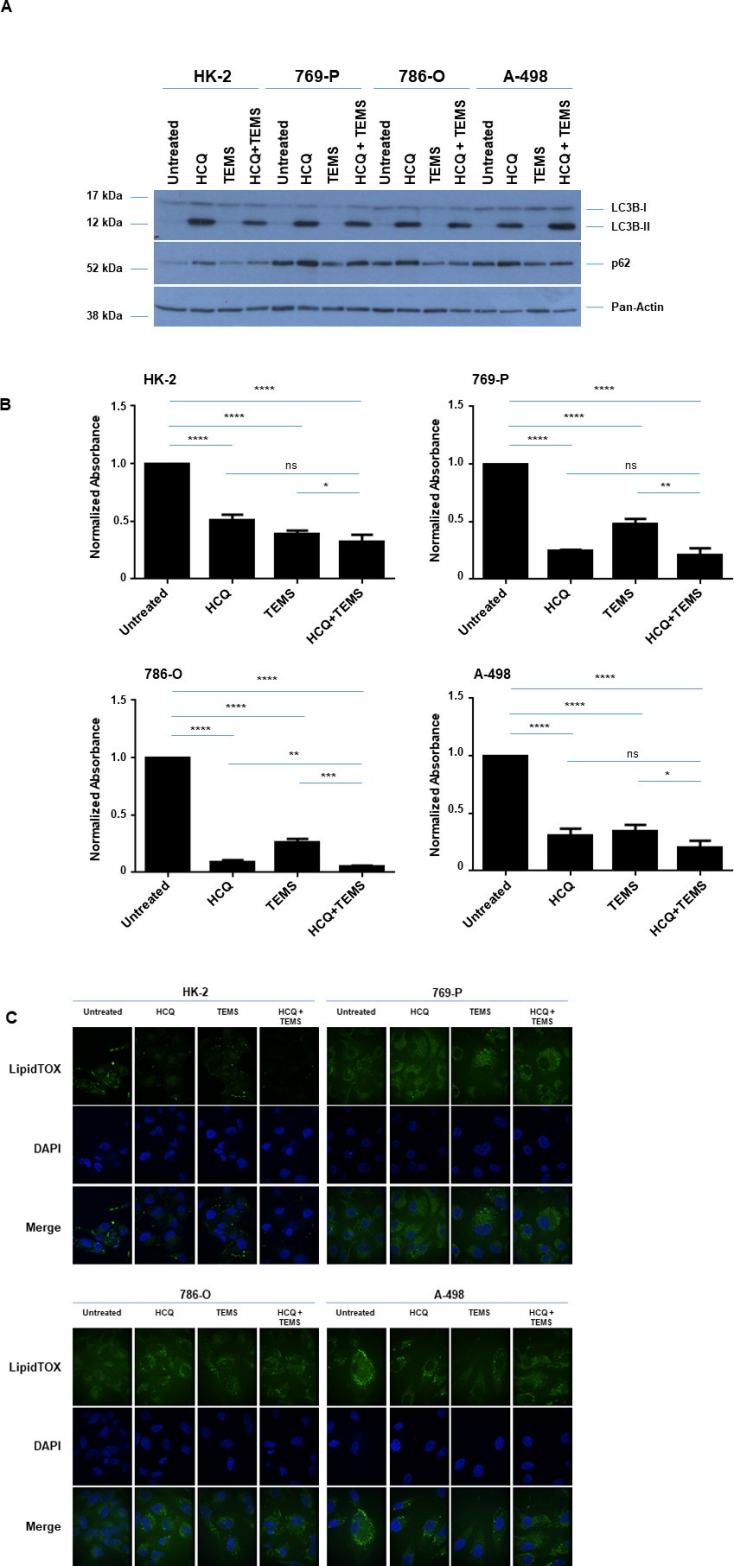
HCQ alters LDs and synergizes with TEMS to reduce cellular viability in renal cell lines. (A) Western blot analyses of ccRCC cell lines and HK-2 cells treated for 72 hours with 25 μM HCQ and/or 10 μM TEMS with the indicated antibodies. Three independent experiments were performed and representative blots are displayed. (B) Assessment of cellular viability in ccRCC cell lines and HK-2 cells at Day 3 in response to 25 μM HCQ and/or 10 μM TEMS. The composite of three independent experiments is displayed. (C) LD assessment of ccRCC cell lines and HK-2 cells in response to 24 hour treatment with 25 μM HCQ and/or 10 μM TEMS via immunofluorescence staining using LipidTOX. Three independent experiments were performed and representative images are displayed.

Since the PI3K/AKT/mTOR pathway negatively regulates autophagy, effective renal cancer cell treatment with mTOR inhibitors (such as TEMS) may thus be antagonized by the resulting increased cellular protective autophagy (7, 8). Indeed, induction of autophagy with mTORC1 inhibition has previously been reported, and thus may provide a mechanism underlying low patient response rates in clinical trials to mTOR inhibitors [9, 10]. In this regard, we assessed whether the combinatorial treatment of hydroxychloroquine (HCQ) with TEMS alters LD abundance in the normal and malignant renal cell lines. We first validated HCQ activity by treating cells (769-P, 786-O, A-498 and HK-2) with 25 μM HCQ alone or in combination with 10 μM TEMS and then detecting lipidated LC3B levels via western blot analyses. As shown in Fig 5A, we observed that LC3B-II and p62 protein levels were increased indicating that autophagic flux was effectively inhibited. Further, we noted a significant reduction in cellular viability in response to the combinatorial HCQ and TEMS treatment in the malignant cell lines but not in the normal HK-2 cells (Fig 5B). These findings suggest that the combinatorial treatment of autophagic and mTOR inhibition is more effective than the individual treatment regimens. Interestingly, upon LipidTOX staining, we noted that the LDs appeared to be sequestered within an autophagosomal compartment (Fig 5C); indeed, HCQ mediates inhibition of the autophagosome-lysosome fusion event [8]. Altogether, these findings suggest that HCQ dominates TEMS-induced alterations in LD abundance potentially leading to diminished LD turnover.

### LPA antagonizes TEMS-induced cellular alterations in a malignant renal cell line

**Fig 6:**
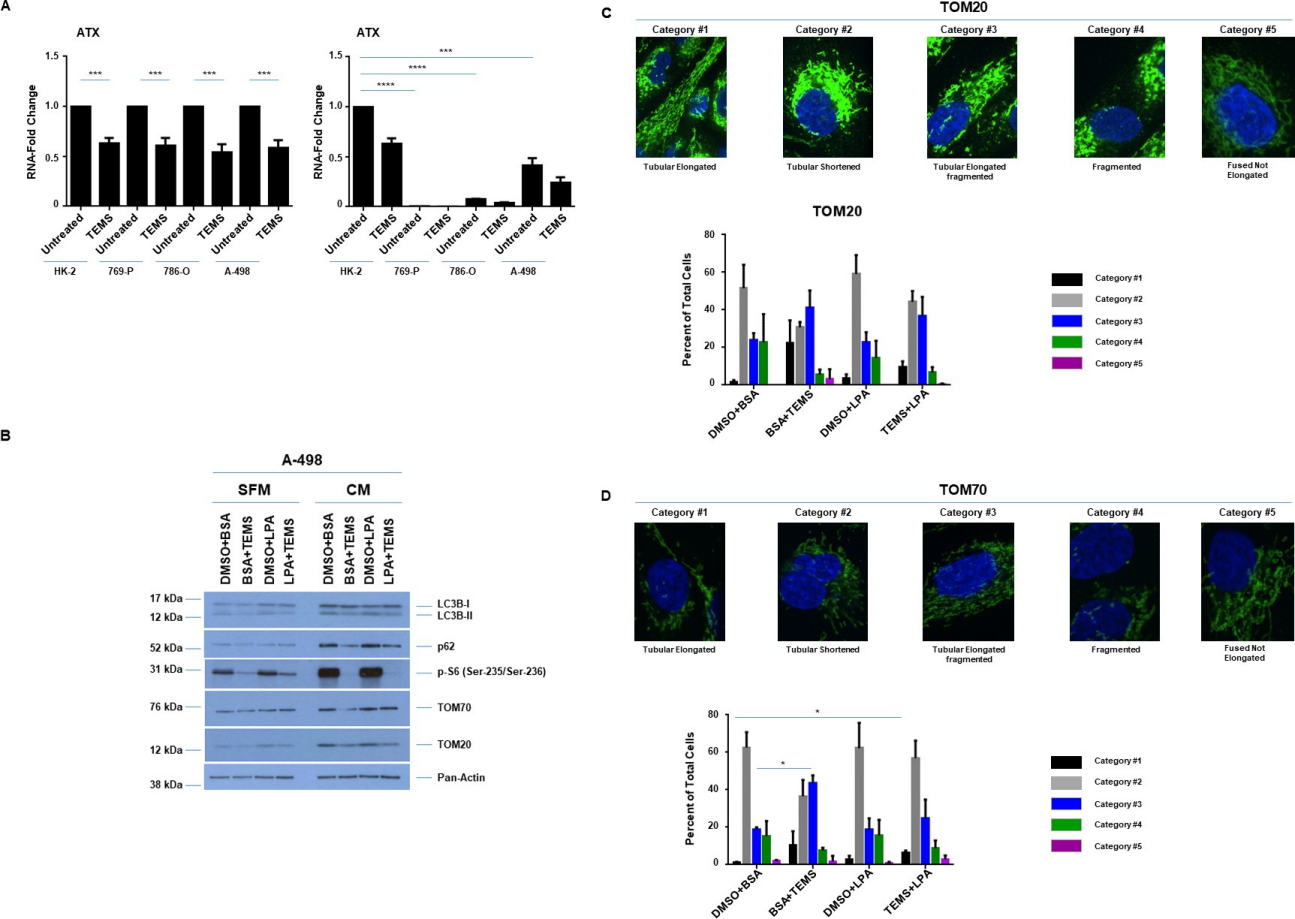
LPA antagonizes TEMS-induced mitochondrial alterations in a malignant renal cell line. (A) Real-time PCR analyses of ATX in ccRCC cell lines and HK-2 cells in response to 24 hour treatment with 10 μM TEMS. Data shown is the composite of three independent experiments. Left panel, normalized to each cell line (untreated). Right panel, normalized to HK-2 cells (untreated). (B) Western blot analyses of A-498 cells (grown in CM or SFM media conditions) treated with 10 μM TEMS in the absence or presence of 10 μM LPA with the indicated antibodies. Three independent experiments were performed and representative blots are displayed. (C) Mitochondrial network assessment of A-498 cells in response to 24 hour treatment with 10 μM TEMS with/without 10 μM LPA via immunofluorescence staining of TOM20. Three independent experiments were performed and representative images are displayed. (D) Mitochondrial network assessment of A-498 cells in response to 24 hour treatment with 10 μM TEMS with/without 10 μM LPA via immunofluorescence staining of TOM70. Three independent experiments were performed and representative images are displayed.

LPA is a potent mitogenic lipid that mediates its extracellular effects via binding and activation of G-protein coupled receptors [11, 12]. Although LPA is elevated (along with autotaxin (ATX)) in kidney cancers [13–15] and can promote malignancy of patient-derived tumor cells [40], the role of this mitogenic lipid in chemotherapeutic resistance with the mTOR inhibitor, TEMS, has not yet been explored. First, we assessed whether ATX levels were altered by TEMS treatment in the malignant and normal renal cell line via real-time PCR. As shown in Fig 6A (top and bottom panels), we noted that ATX mRNA was significantly reduced following 24 hours of TEMS treatment and unexpectedly, these levels were significantly lower in ccRCC cells in comparison to HK-2 cells. Since LPA mediates its effects via GPCRs to activate multiple intracellular signaling pathways including AKT/mTOR [11, 12], we next assessed whether addition of LPA alters the AKT/mTOR pathway in a malignant renal tumor cell line. The dose of LPA utilized (10 μM) was based on reported pathophysiological concentrations identified within tumors and in serum [41]. Specifically, we selected A-498 cells to investigate whether LPA modulates TEMS responsiveness since these cells displayed marked changes in LDs and mitochondrial networks in addition to harboring the most genomic aberrations (out of the three malignant RCC lines studied herein). We cultured A-498 cells in both serum free media (SFM) and complete media (CM) to determine the effect of exogenously presented LPA in these cells. As shown in Fig 6B, we observed that the reduction in protein expression in phospho-S6, TOM20, TOM70, LC3B-II, and p62 under both culture conditions (consistently noted in SFM) were increased nearly to baseline control levels in the presence of LPA with TEMS.

**Fig 7:**
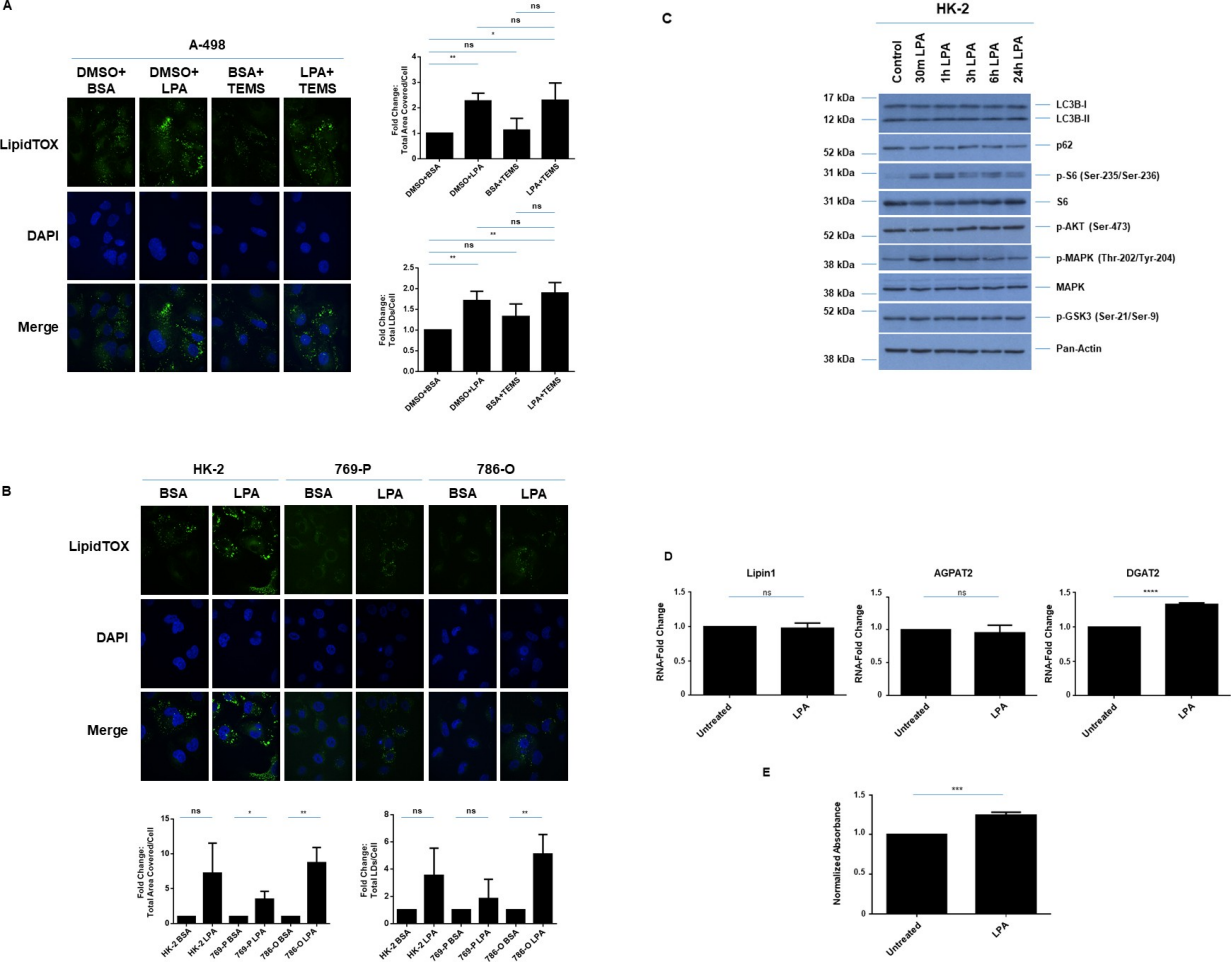
LPA increases LDs in a malignant renal cell line. (A) LD assessment of A-498 cells in response to 24 hour treatment with 10 μM LPA in the absence/presence of 10 μM TEMS via immunofluorescence staining using LipidTOX. Three independent experiments were performed and representative images are displayed as well as quantification of Total Area Covered/Cell and Total LDs/Cell (presented as normalized Fold-Change). (B) LD assessment of ccRCC cell lines and HK-2 cells in response to 24 hour treatment with 10 μM LPA in the absence/presence of 10 μM TEMS via immunofluorescence staining using LipidTOX. Three independent experiments were performed and representative images are displayed as well as quantification of Total Area Covered/Cell and Total LDs/Cell (presented as normalized Fold-Change). (C) Western blot analyses of HK-2 cells treated with 10 μM LPA across a time course (30 minutes to 24 hours) with the indicated antibodies. Three independent experiments were performed and representative blots are displayed. (D) Real-time PCR analyses of genes involved in LD biogenesis and regulation in HK-2 cells in response to 24 hour treatment with 10 μM LPA. Data shown is the composite of three independent experiments. (E) Cell viability in HK-2 cells was assessed following LPA treatment for 3 days. Data shown is the composite of three independent experiments.

We next assessed whether LPA could antagonize TEMS-induced alterations in mitochondrial networks via immunofluorescence analyses (staining for TOM20 and TOM70) in A-498 cells (Fig 6C and 6D). Since the SFM culture conditions led to shrunken cells, which resulted in challenges in assessing the mitochondrial networks, we focused this assessment using A-498 cells maintained in complete media. In control cells, we observed mitochondrial networking (staining for TOM20 and TOM70 were similar) close to the nuclear compartment of which most had a fragmented appearance (with a few extensions). LPA treatment alone did not modulate this mitochondrial network and TEMS treatment, itself, caused the mitochondrial network to become more extended (Fig 3D). Interestingly, the addition of LPA with TEMS cause a marked rearrangement of the mitochondrial networks to a tubular shortened form, which we quantified (bottom panels, Fig 6C and 6D). These findings suggest that LPA has the ability to partially antagonize the cellular response to TEMS. Since the contribution of LPA in modulating LD biogenesis remains unknown which may thus contribute to its pro-tumorigenic effect, we next investigated LD formation upon cellular treatment with LPA in A-498 cells using CM (Fig 7A) culture conditions. In control cells, we observed that 60–70% cells contained large LDs homogeneously dispersed in the cytoplasm with the remaining 30–40% of the cell population containing finer LDs. In the presence of LPA, nearly the entire cell population contained large LDs that were homogeneously dispersed in the cytoplasm. In contrast to TEMS-treated cells which contained finer LDs in 80% of the cell population, the combinatorial treatment of TEMS with LPA resulted in nearly the entire population containing large LDs homogeneously distributed throughout the cell cytoplasm. These findings suggest that LPA may antagonize the LD-induced changes mediated by TEMS.

**Fig 8:**
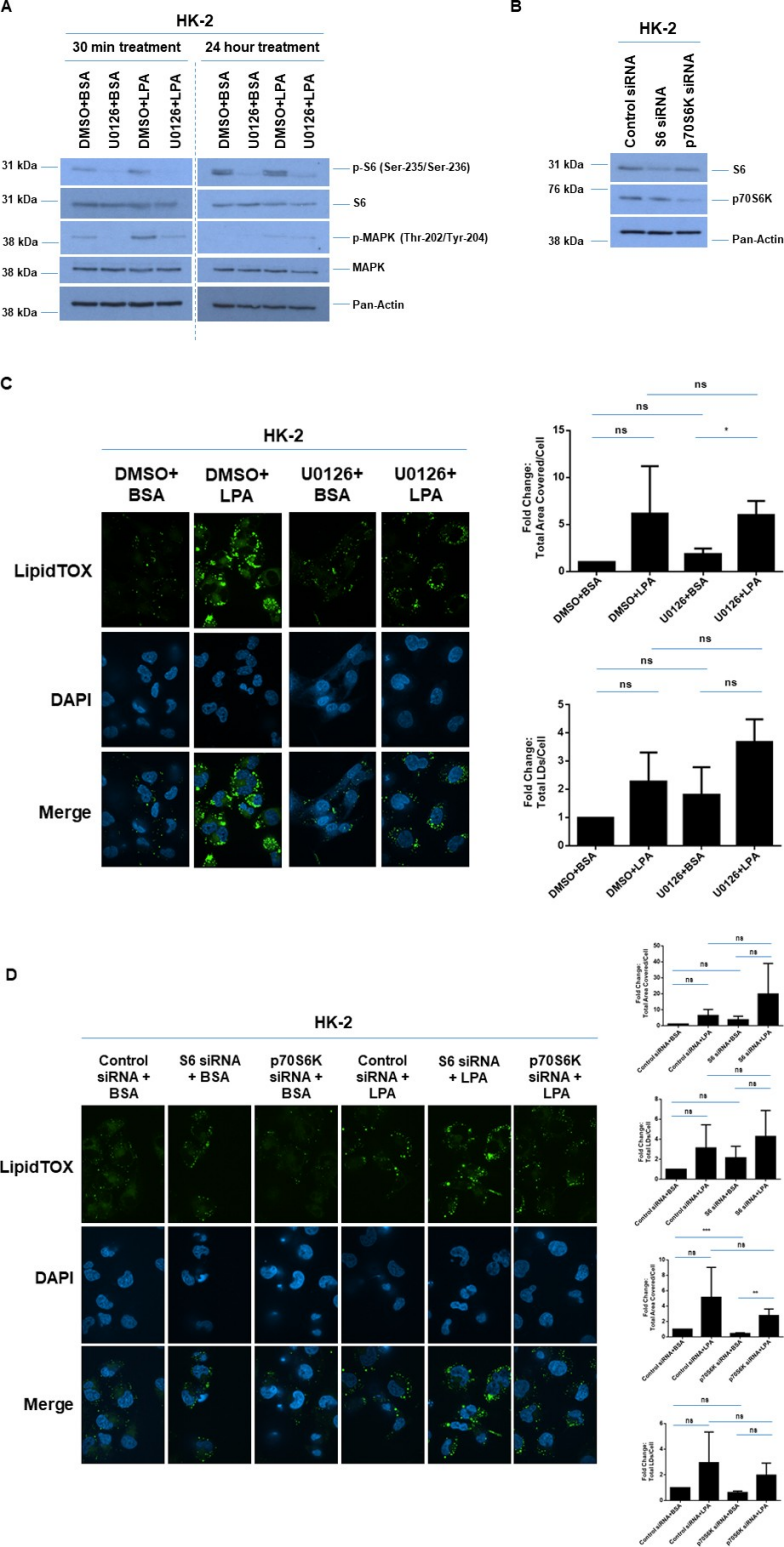
LPA alters LD abundance via the MAPK pathway and cellular viability in HK-2 cells. (A) Western blot analyses of HK-2 cells treated with 10 μM LPA in the absence of presence of U0126 for 30 minutes (left panels) or 24 hours (right panels) with the indicated antibodies. Three independent experiments were performed and representative blots are displayed. (B) Western blot analyses of HK-2 cells treated with S6 or p70S6K siRNA with the indicated antibodies. Three independent experiments were performed and representative blots are displayed. (C) LD assessment of HK-2 cells in response to 24 hour treatment with 10 μM LPA in the absence/presence of U0126 via immunofluorescence staining using LipidTOX. Three independent experiments were performed and representative images are displayed as well as quantification of Total Area Covered/Cell and Total LDs/Cell (presented as normalized Fold-Change). (D) LD assessment of HK-2 cells in response to 24 hour treatment with 10 μM LPA in the absence/presence of siRNA targeting S6 or p70S6K via immunofluorescence staining using LipidTOX. Three independent experiments were performed and representative images are displayed as well as quantification of Total Area Covered/Cell and Total LDs/Cell (presented as normalized Fold-Change).

**LPA increases LDs in renal cell lines and alters cellular viability of the normal immortalized HK-2 cells.** The alterations in LDs mediated by LPA in A-498 cells were generalizable to other renal cell lines including two other malignant cell lines (769-P and 786-O cells) as well as the normal immortalized HK-2 cells (Fig 7B). Indeed, we uncovered that LPA caused the formation of larger LDs in all of these renal cell lines. To assess the mechanism underlying alterations in LD formation upon LPA treatment, we performed western blot analysis using lysates from HK-2 cells maintained in complete media culture conditions treated with LPA across a time course (30 minutes up to 24 hours). As shown in Fig 7C, we observed MAPK and S6 activation in the absence of phospho-AKT or phospho-GSK3 alterations as early as 30 minutes following LPA addition. These levels appeared to be sustained up to 24 hours of LPA treatment. These changes were accompanied by elevated mRNA levels of DGAT2 in HK-2 cells (Fig 7D) suggesting that the increased LDs may be due to increased production and activity of enzymes in LD biogenesis. As shown in Fig 7E, we observed a statistically significant increase in cell viability in HK-2 cells following LPA treatment for 3 days. These findings suggest that LPA can enhance the tumor-promoting effect of kidney cells *in vitro*. To determine whether LPA-induced activation of the MAPK and/or S6 signaling cascade contributed to the LPA-induced LD formation in HK-2 cells, we inhibited MAPK using 10 μM of U0126 and inhibited S6 as well as p70S6K using siRNA. We confirmed the effectiveness of these inhibitors via western blot analyses (Fig 8A (at 30 minutes of LPA treatment) and 8B). We also analyzed LD abundance using LipidTOX (Fig 8C and 8D) and noted increased numbers of finer LDs associated following MAPK inhibition; however, the larger LDs induced by LPA alone were reduced following co-treatment with TEMS. Interestingly, we noticed that S6 protein reduction led to an increased number and size of LDs even in the absence of LPA, which was further enhanced with addition of LPA. These findings suggest that LPA mediates alterations in LDs partially via MAPK activation and was independent of S6/p70S6K.

## Discussion

Kidney tumors harbor a myriad of genetic alterations, including mutation or loss of the *VHL* gene, a tumor suppressor which is a common occurrence in >90% of clear cell kidney cancer cases [1, 24]. The ccRCC cell lines used for this study (769-P, 786-O and A-498) are associated with aberrations in VHL (TCGA data: Fig 1A). mTOR is a downstream effector of PI3K/AKT, a pathway that is well-established to be hyperactivated in ccRCC [4, 42]. Furthermore, several agents targeting mTOR have been developed including FDA-approved drugs such as everolimus and temsirolimus (TEMS) [4]. However, resistance to these agents has been a major contributor to suboptimal overall survival rates.

We now have identified that the mTOR inhibitor TEMS alters LD quality in both RCC and normal immortalized HK-2 cells, possibly to enhance lipid mobilization, which antagonizes cellular sensitivity to the treatment (Figs 2B and 3A). We also demonstrate that TEMS reduces expression of autophagy markers, which is accompanied by a decrease in expression of mitochondrial membrane proteins (TOM20, TOM40, and TOM70) and mitochondrial fused networks (Fig 3D and 3E). These cellular alterations may serve to relieve cellular stress induced by TEMS, thus promoting β-oxidation and fatty acid flux [6]. Since mitochondrial networks are highly dynamic in cancer and contribute to cancer cell proliferation, migration and altered cellular metabolism [43], it is notable that LDs have been found in close proximity to the ER and mitochondrial compartments to facilitate lipid exchange across these organelles [44]. Thus, the mitochondrial remodeling noted following TEMS treatment in the malignant renal cancer cell lines may thus promote increased energy usage to antagonize responsiveness to the mTOR inhibitor. Remodeling of mitochondria is likely to occur via regulated fission and fusion dynamics involving proteins present in the outer and inner mitochondrial membrane including MFN1/2, Opa1, and DRP1 [43, 44]. Furthermore, there is another report that describes that inhibitors of mTOR can lead to hyperfused and branched mitochondria, for which the underlying mechanism is thought to involve reduced expression of the mitochondrial fission process 1 (MTFP1) and DRP1 mitochondrial recruitment which may then regulate cellular survival [45].

With respect to increased LDs in response to exogenous supplementation with oleic acid, this cellular response appears to be similar to that with fatty acid synthase (FAS) inhibitors along with mTOR inhibitors which results in synergistic toxicity in breast cancer cells. Moreover, the mTOR inhibitor can modulate expression of FASN, which implicates the PI3K/AKT/mTOR pathway in its regulation [46]. Thus, these pathways may be similarly involved with oleic acid exogenous supplementation in our renal cell lines.

Suboptimal or ineffective cellular responses to mTOR inhibitors may be due to activation of cytoprotective pathways including autophagic flux [9, 30, 31, 47]. We now demonstrate that the autophagic flux inhibitor (HCQ) alters LD abundance in both malignant and normal renal cell lines (Fig 5C). The accumulation of LDs with HCQ appeared to occur within autophagosomes, implicating inhibition of the lipophagy pathway and hence promoting synergistic cytotoxicity of HCQ and TEMS. Recently, combinatorial treatment of HCQ with the mTOR inhibitor Everolimus was assessed in a phase 1 clinical trial, which showed promise as an effective therapeutic regimen [30]. Additional pathways that may antagonize mTOR inhibitors include feedback activation of the AKT signaling pathway [48, 49]. Indeed, targeting components in these upstream PI3K-AKT cascade (as a multipronged approach) has been tested [48, 49].

Since ATX (which generates the potent LPA mitogen via its potent lyso-PLD activity) is elevated in ccRCC [13, 41] and has been shown to mediate chemotherapeutic resistance to sunitinib [14], this may be another mechanism underlying resistance to mTOR inhibitors. Indeed, we noted that LPA could antagonize the cellular responsiveness of malignant renal cell lines to TEMS, in terms of LDs and mitochondrial remodelling. LPA is linked to promotion of cell survival and proliferation. For example, treatment of a rat myocardial infarction model with LPA promoted cardiac hypertrophy which coincided with autophagic inhibition [50]; furthermore, this was noted to occur via activation of the AKT/mTOR pathway [50]. Activation of this pathway following LPA treatment is well established, as defined from other studies as well [51–53]. Therefore, in future work, we will investigate the combinatorial treatment of LPA with TEMS compared to LPA alone in RCC cell lines.

Furthermore, since we demonstrated that LPA with TEMS treatment resulted in a rearrangement of mitochondrial networks to a tubular shortened form, this could involve altered LDs and autophagic response, leading to altered mitochondrial structures. Indeed, it is reported that starvation can promote movement of autophagy-mediated LDs to mitochondria (needed for fatty acid β-oxidation, where fatty acids are made available via the action of neutral lipases), thus affecting fusion dynamics [6]. Such LDs are noted to be in close proximity to mitochondria to allow the uptake of fatty acids. Based on this report, we propose that LPA-induced LD formation and TEMS-induced autophagy may work coordinately to alter mitochondrial networks [6]. In our results, we show that LPA and TEMS combinatorial treatment results in a tubular shortened form of mitochondrial networking, suggesting that there may be resultant alterations in fatty acid movement and hence, altered β-oxidative activity. Thus, altered mitochondrial networking may result in restricted movement of fatty acids from lipid droplets to mitochondria (possibly, its expulsion from the cells thus a reduction in fatty acid metabolism would ensue) and hence, lipid accumulation in LDs.

Interestingly, we also noted that ATX mRNA levels were reduced with TEMS treatment (Fig 6A). Although we did not measure LPA content in the culture media following TEMS treatment, we propose that LPA may be elevated with mTOR inhibition and thus may downregulate ATX expression in a feedback loop [54]. In future work, the contribution of LDs to LPA-mediated drug resistance in renal cancers could be investigated. We propose that targeting the LPA/ATX axis may be a strategy to improve the sensitivity of ccRCC tumors to chemotherapeutic agents.

The molecular mechanisms involved in chemotherapeutic resistance in ccRCC are not well established. Herein, we identify that LPA can antagonize the cellular response of renal cancer cells to TEMS, specifically altering lipid droplets and mitochondrial networks. Moreover, in normal immortalized renal cells, we discovered that LPA induced LD formation in a MAPK-dependent manner, which was accompanied by changes in DGAT2 and cellular viability. Overall, this study implicates the LPA signaling pathway as an important target for combating the resistance acquired by RCC cells towards molecular-based therapies.

## Supporting information

S1 Raw images (PPTX)

## Abbreviations

ATGL: Adipose triglyceride lipase
ATX: Autotaxin
CIDEC: Cell death inducing DFFA like effector C
ccRCC: Clear cell renal cell carcinoma
CM: Complete media
DAPI: 4′,6-diamidino-2-phenylindole
DAG: Diacylglycerol
DGAT2: Diacylglycerol O-aceyltransferase 2
DMSO: Dimethylsulfoxide
DRP1: Dynamin-related protein 1
ENPP2: Ectonucleotide pyrophosphatase
FAF-BSA: Fatty-acid free bovine serum albumin
LDs: Lipid droplets
GSK3: Glycogen synthase kinase 3
HCQ: Hydroxychloroquine
LC3B: Microtubule associated protein 1 light chain 3 beta
LPA: Lysophosphatidic acid
LPC: Lysophosphatidylcholine
mTOR: Mechanistic target of rapamycin kinase
PARP1: Poly(ADP-ribose) polymerase 1
MAPK: Mitogen-activated protein kinase 1
PLIN1: Perilipin 1
PLPP1: Phospholipid phosphatase 1
PNPLA2: Patatin like phospholipase domain containing 2
SFM: Serum free media
SQSTM1/p62: Sequestosome 1
TAG: Triacylglycerol
TCGA: The Cancer Genome Atlas
TEMS: Temsirolimus
TOM: Translocase of outer mitochondrial membrane
VHL: Von Hippel-Lindau tumor suppressor
