# Supplementary material for: Lysophosphatidic acid reverses Temsirolimus-induced changes in lipid droplets and mitochondrial networks in renal cancer cells
Source: PLoS One. 2020 Jun 3;15(6):e0233887. doi: 10.1371/journal.pone.0233887 (PMC7269261; doi:10.1371/journal.pone.0233887)
Supplement: S1 Raw images — (PPTX) [file pone.0233887.s001.pptx]

## Slide 1
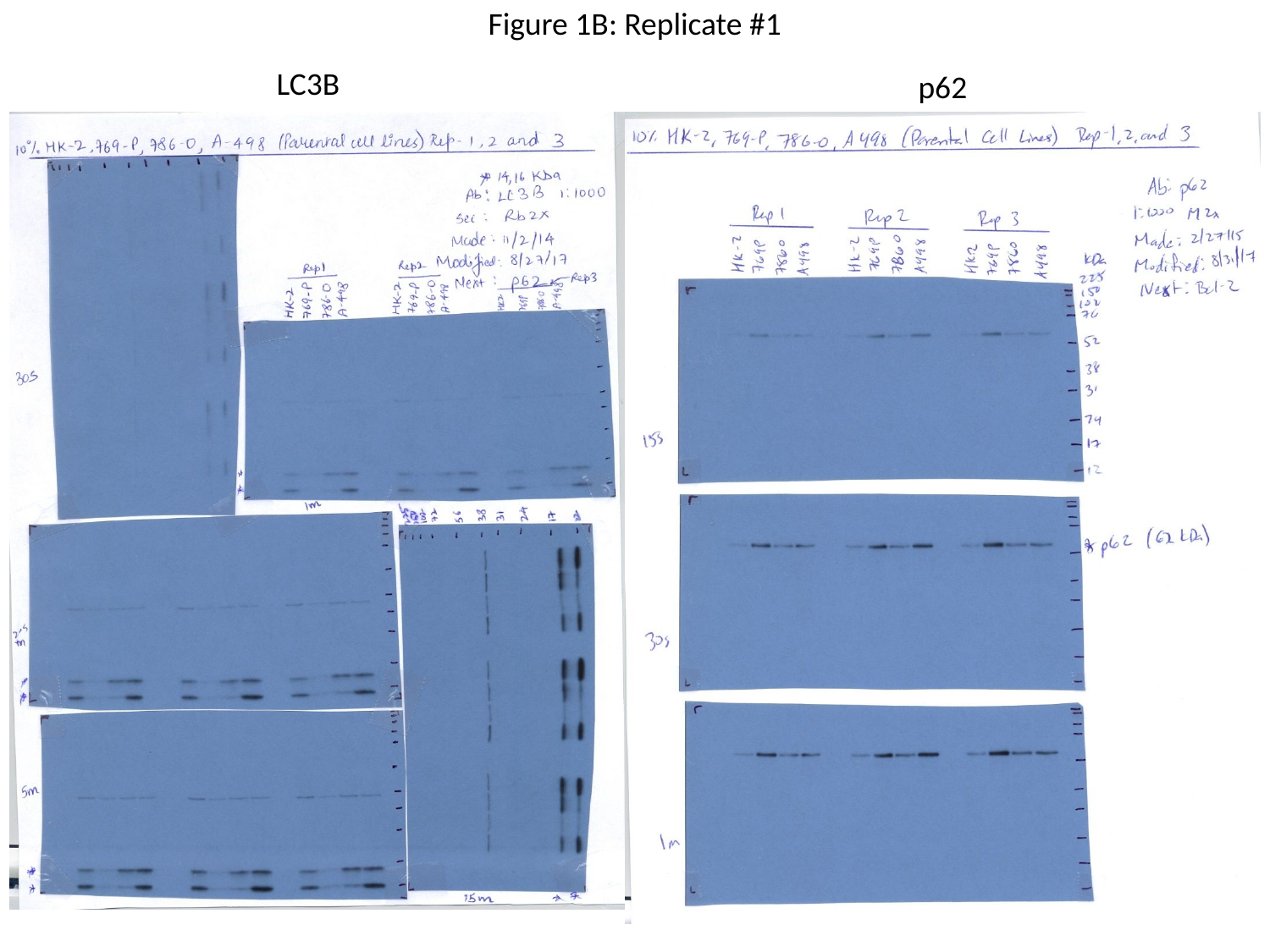

Figure 1B: Replicate #1
LC3B
p62

## Slide 2
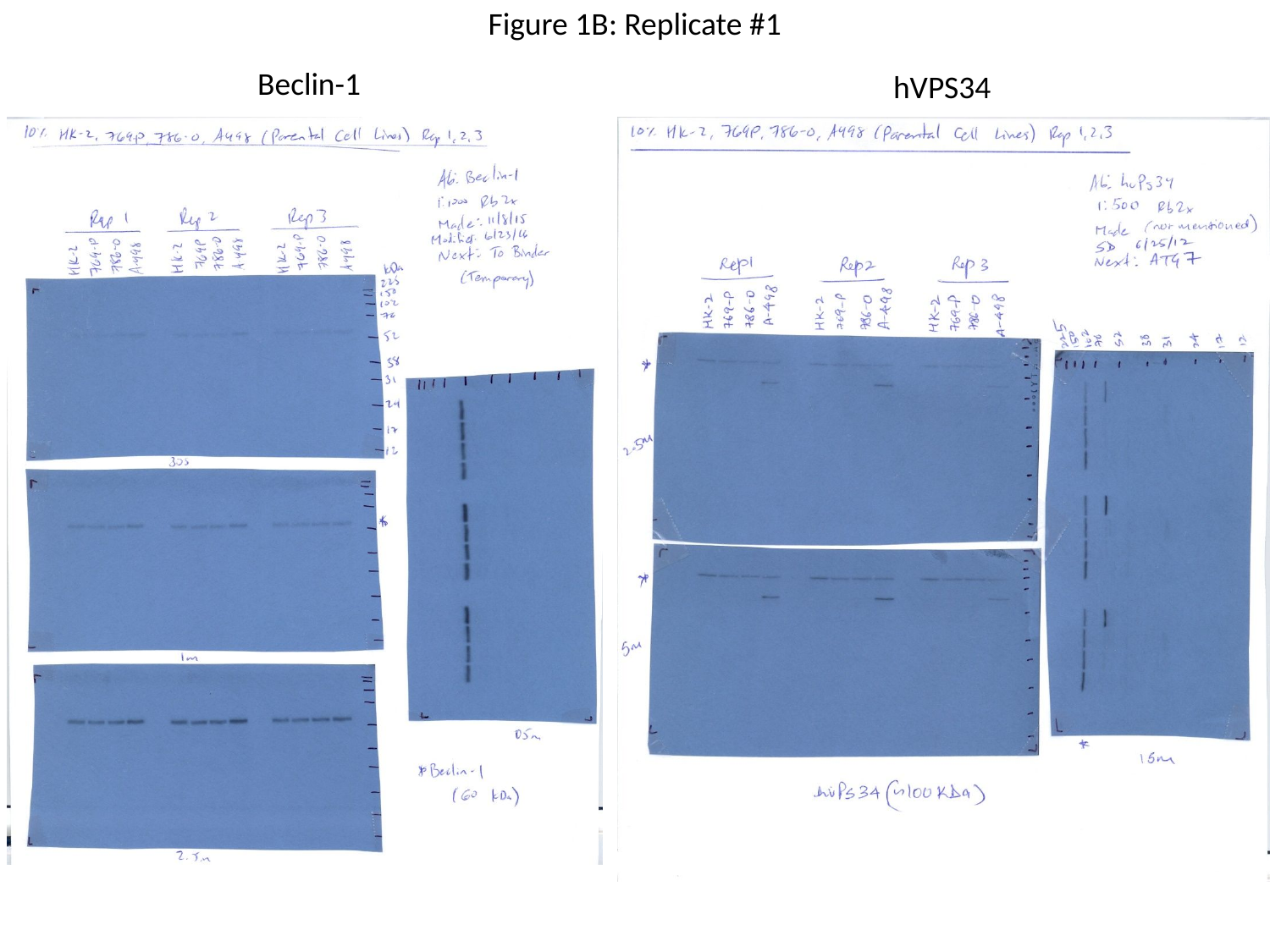

Figure 1B: Replicate #1
Beclin-1
hVPS34

## Slide 3
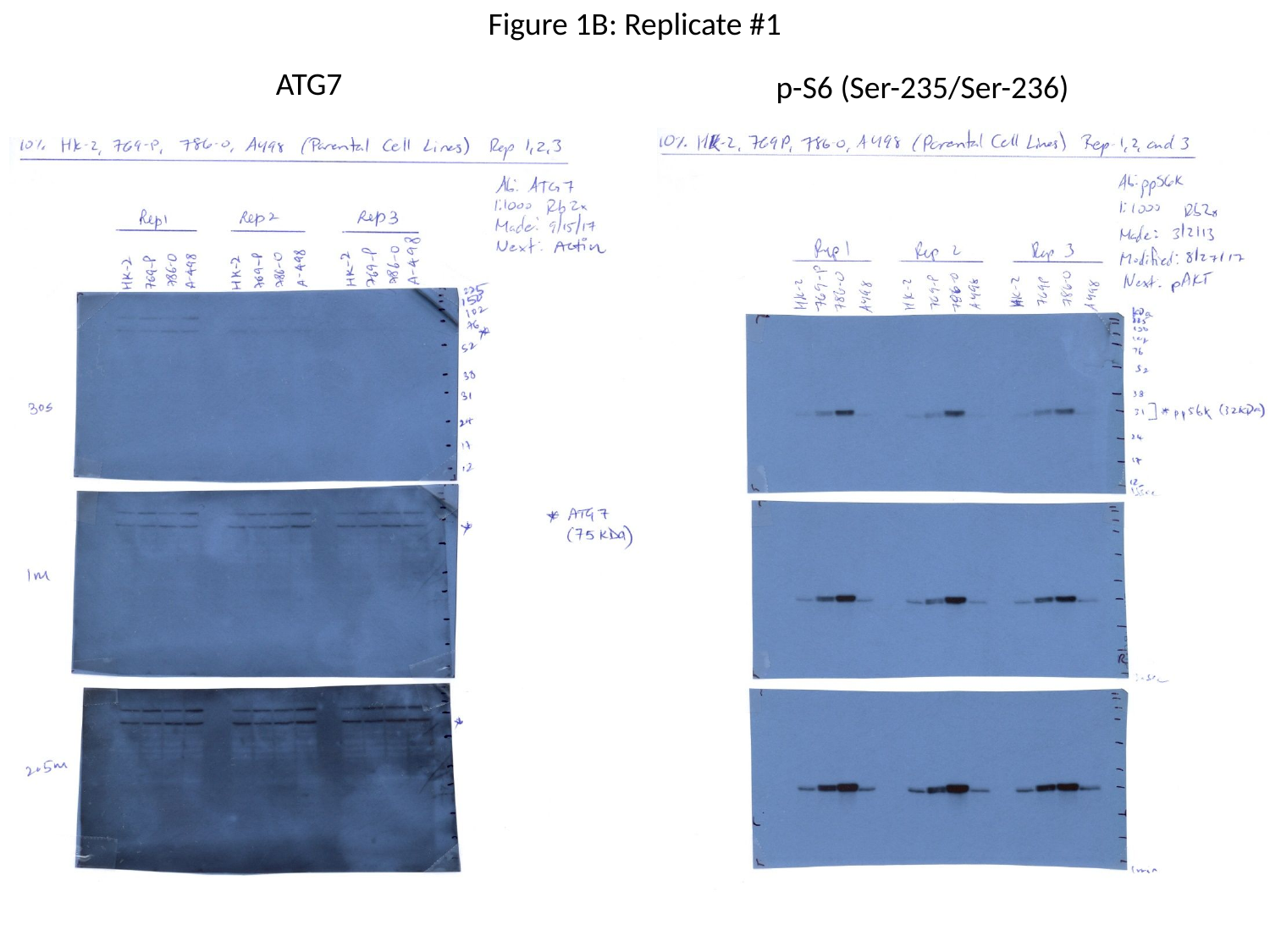

Figure 1B: Replicate #1
ATG7
p-S6 (Ser-235/Ser-236)

## Slide 4
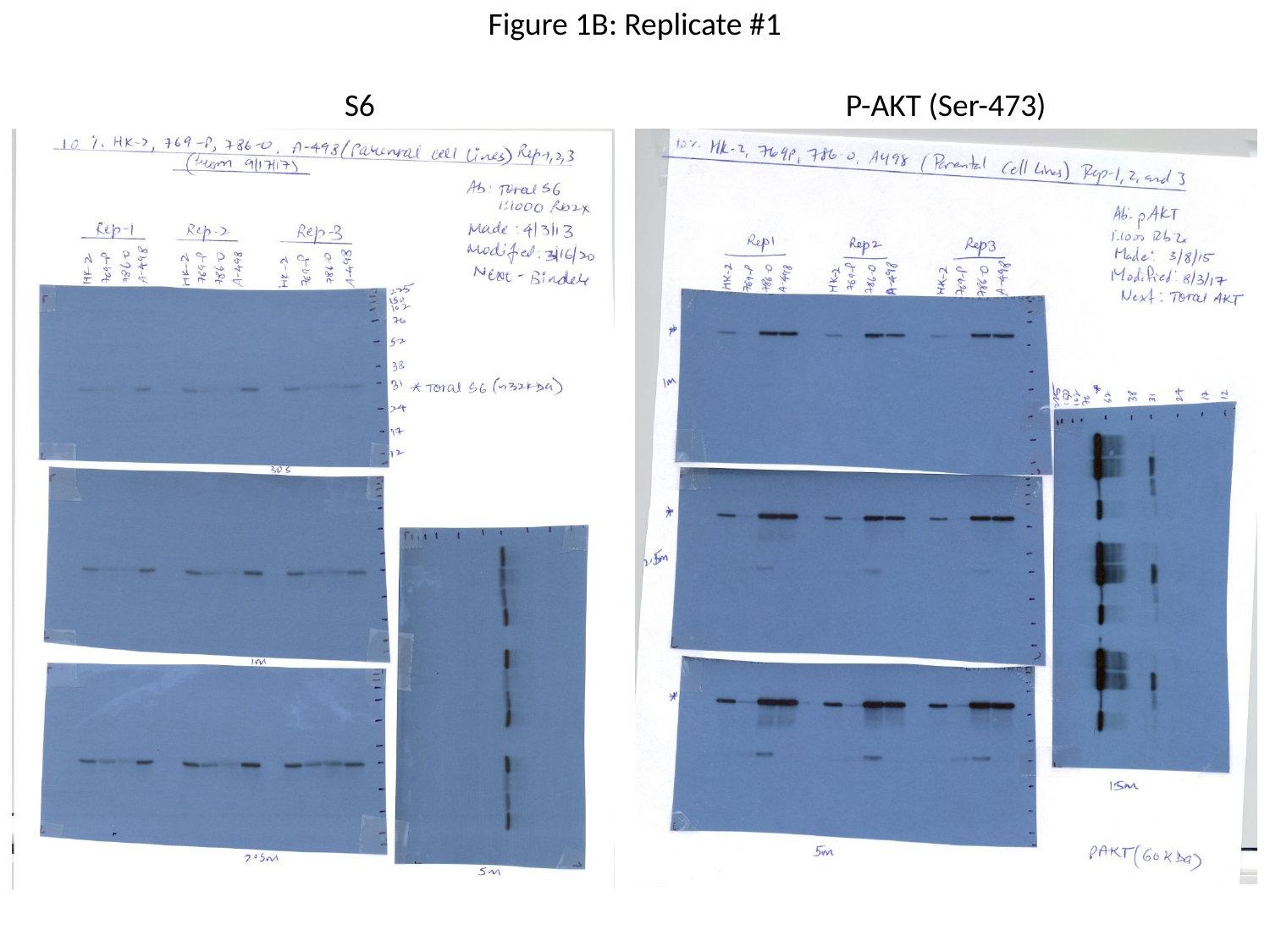

Figure 1B: Replicate #1
S6
P-AKT (Ser-473)

## Slide 5
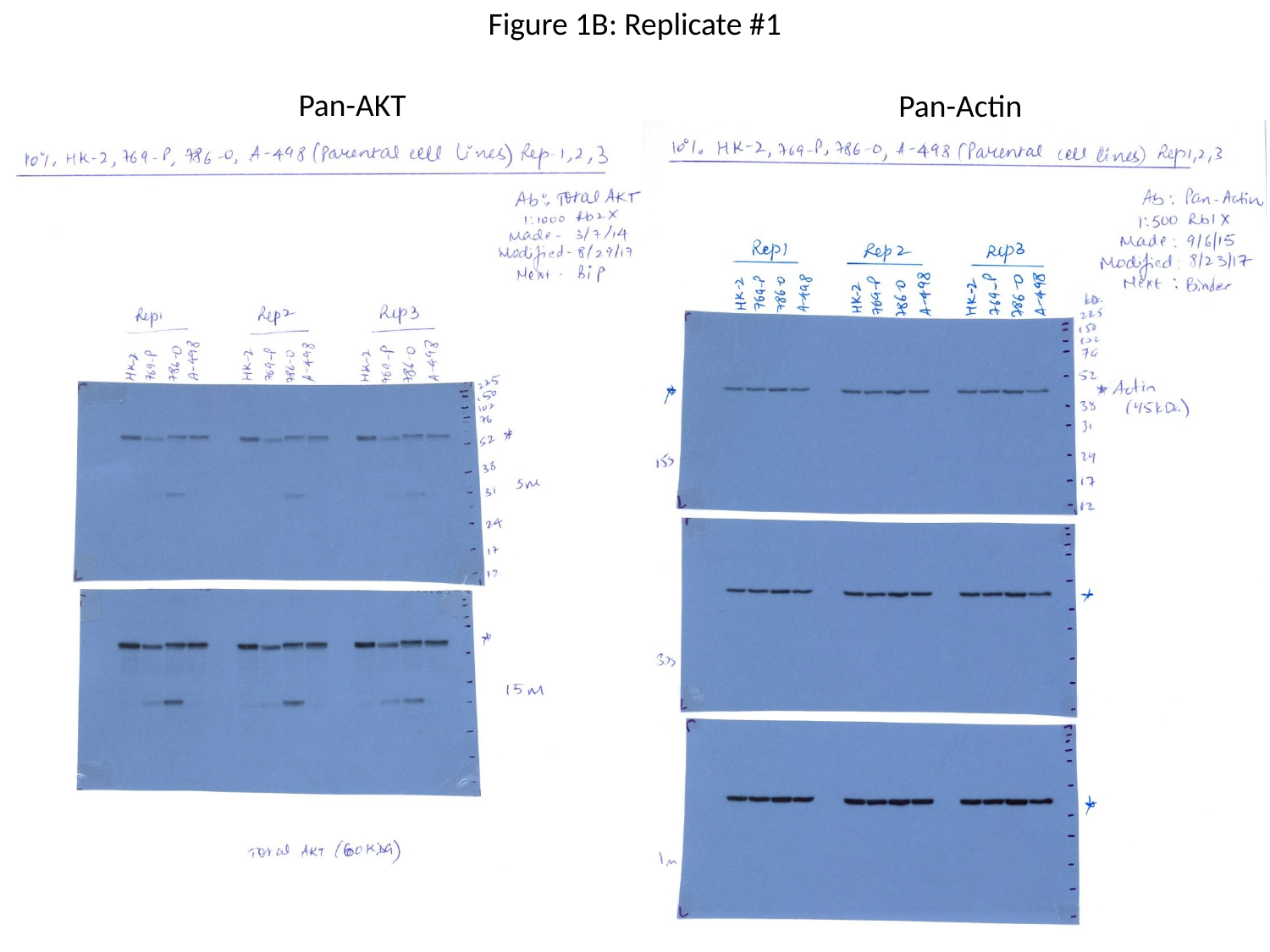

Figure 1B: Replicate #1
Pan-AKT
Pan-Actin

## Slide 6
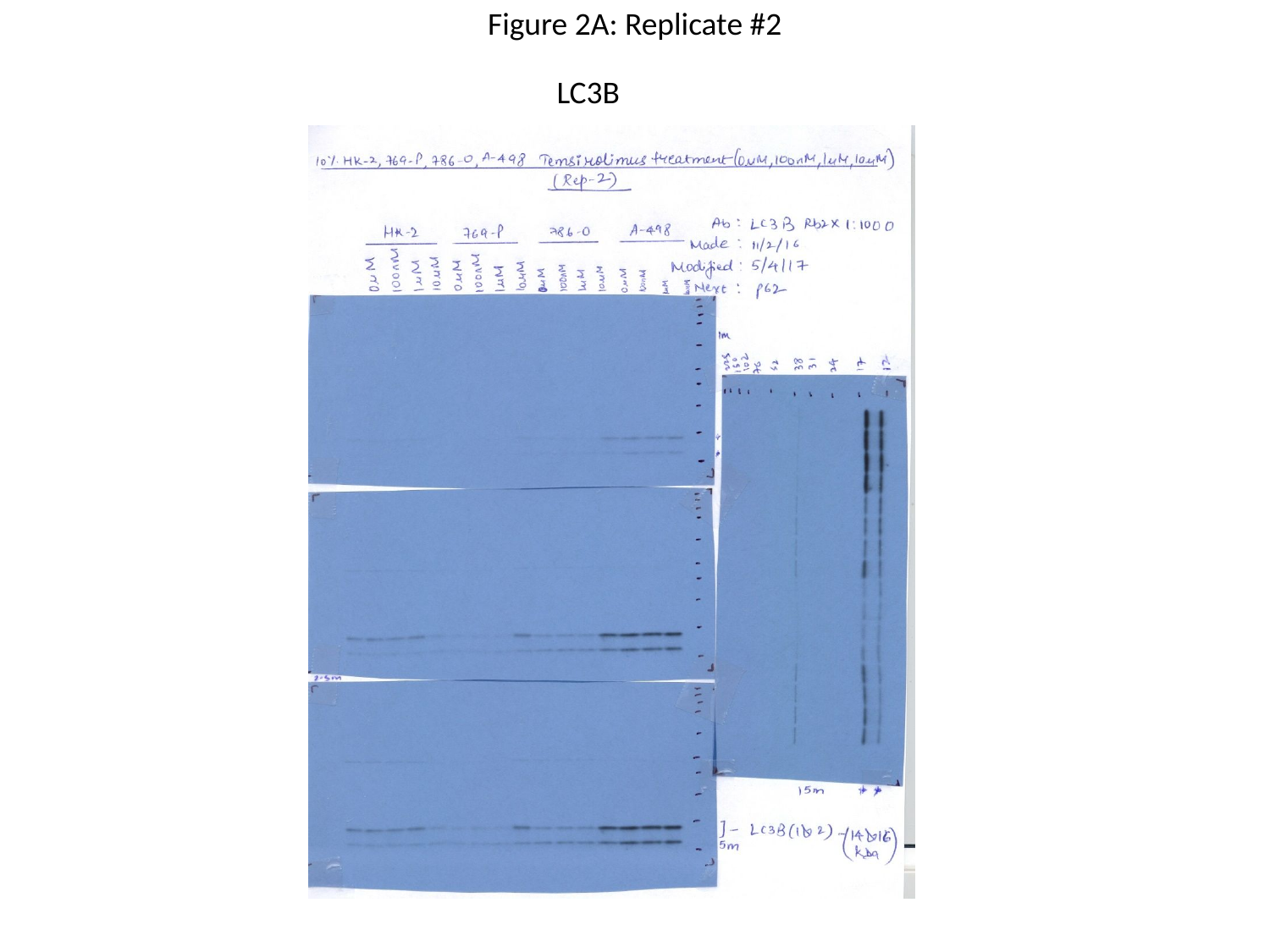

Figure 2A: Replicate #2
LC3B

## Slide 7
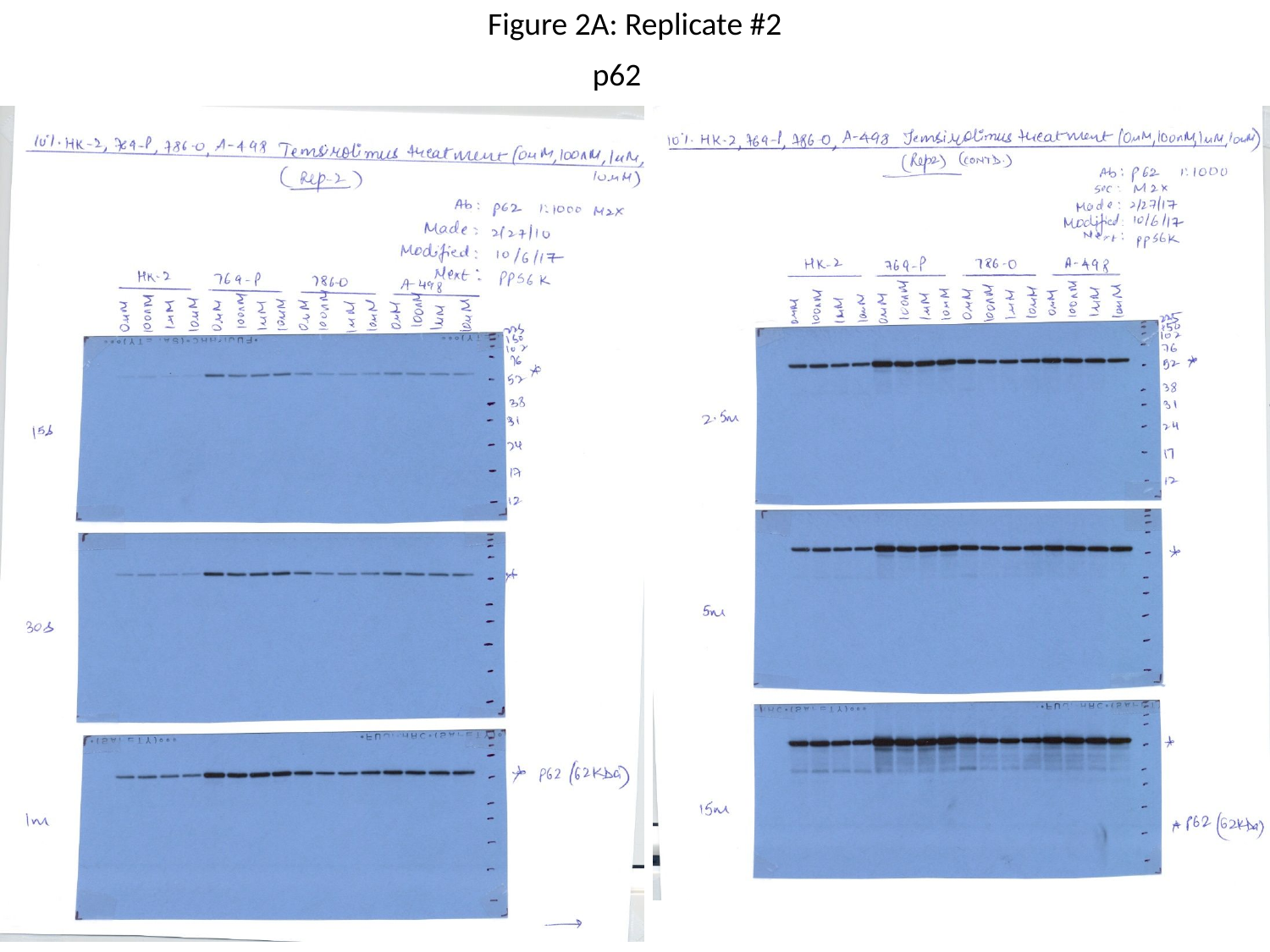

Figure 2A: Replicate #2
p62

## Slide 8
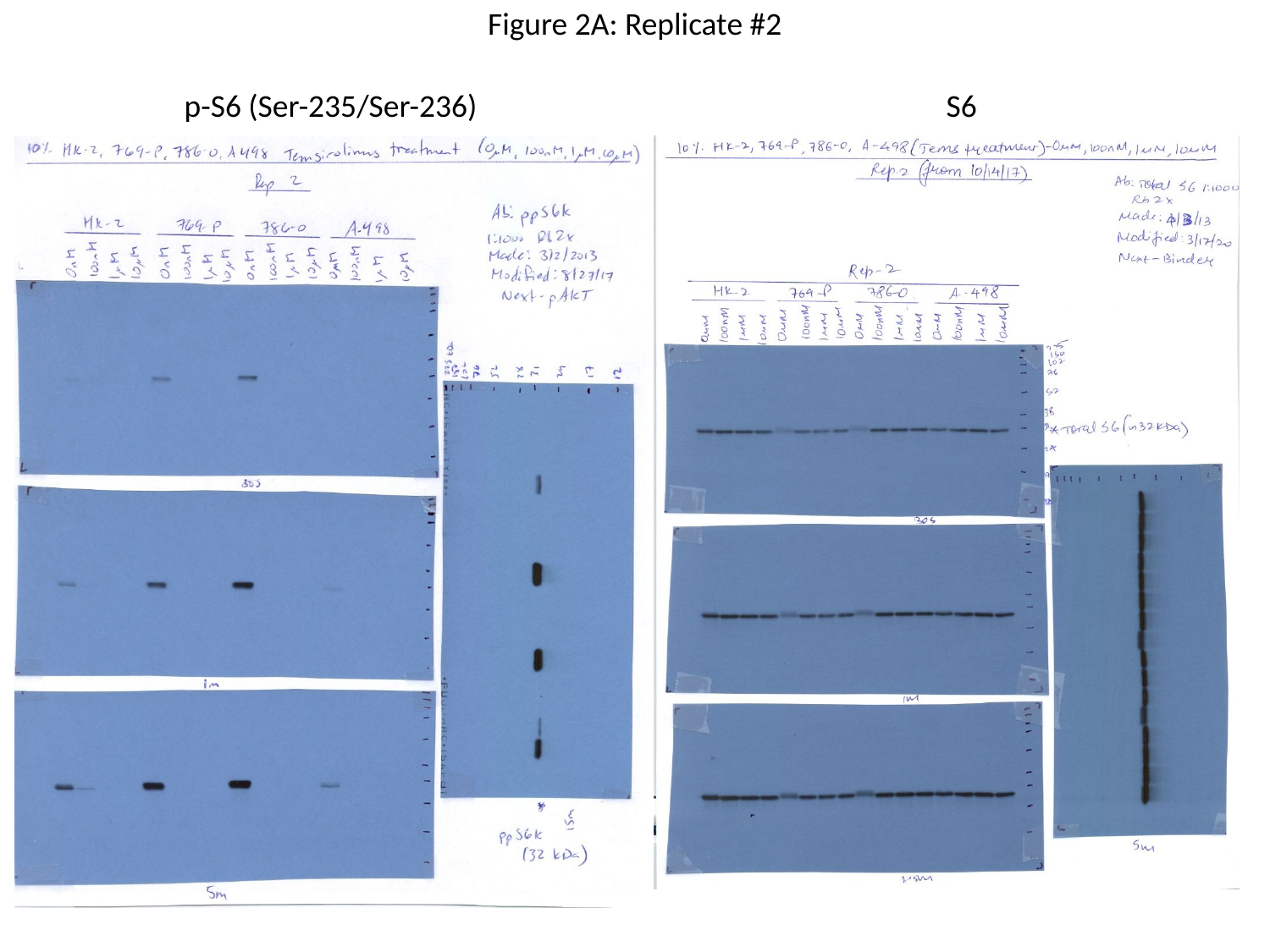

Figure 2A: Replicate #2
p-S6 (Ser-235/Ser-236)
S6

## Slide 9
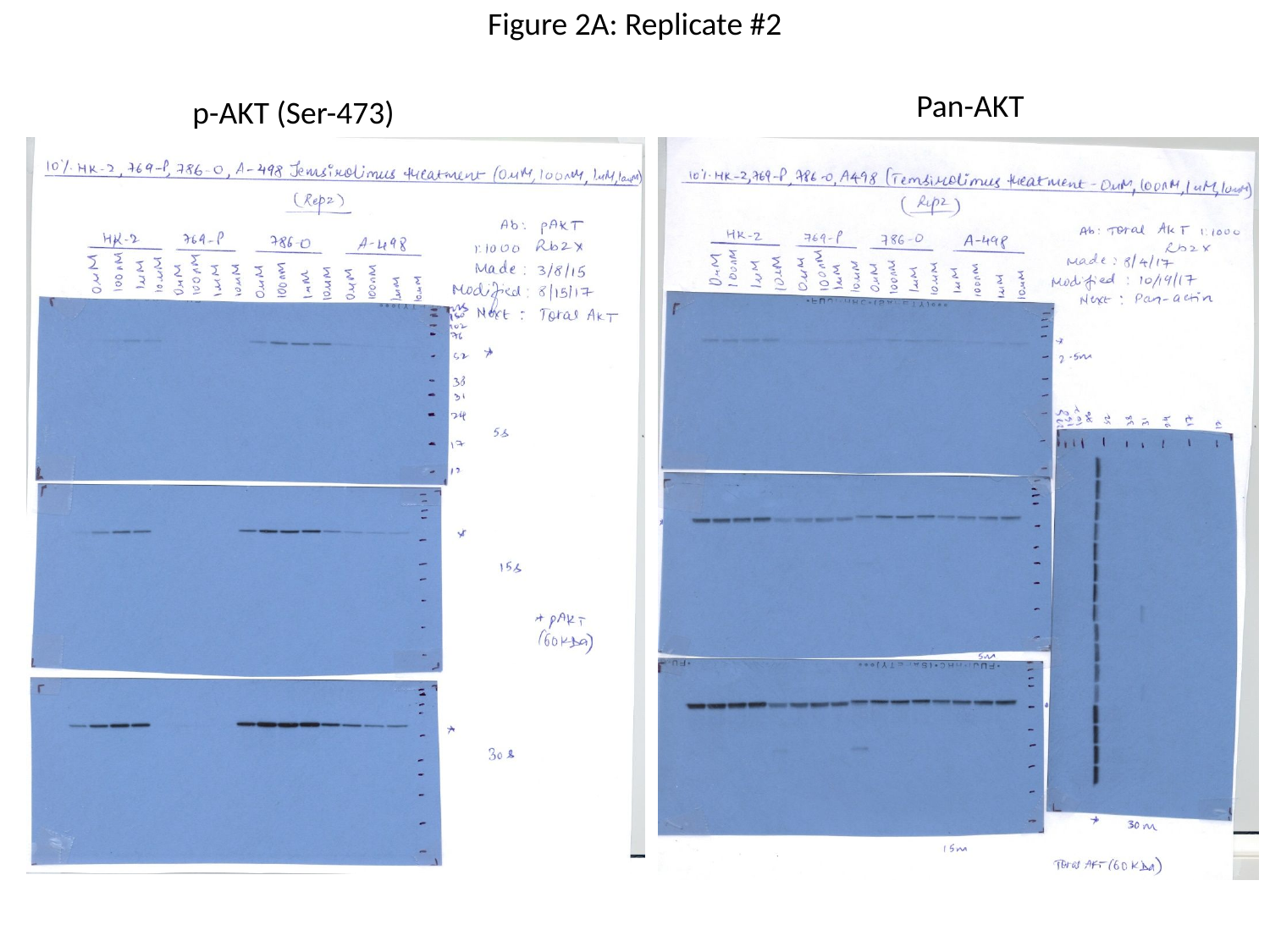

Figure 2A: Replicate #2
Pan-AKT
p-AKT (Ser-473)

## Slide 10
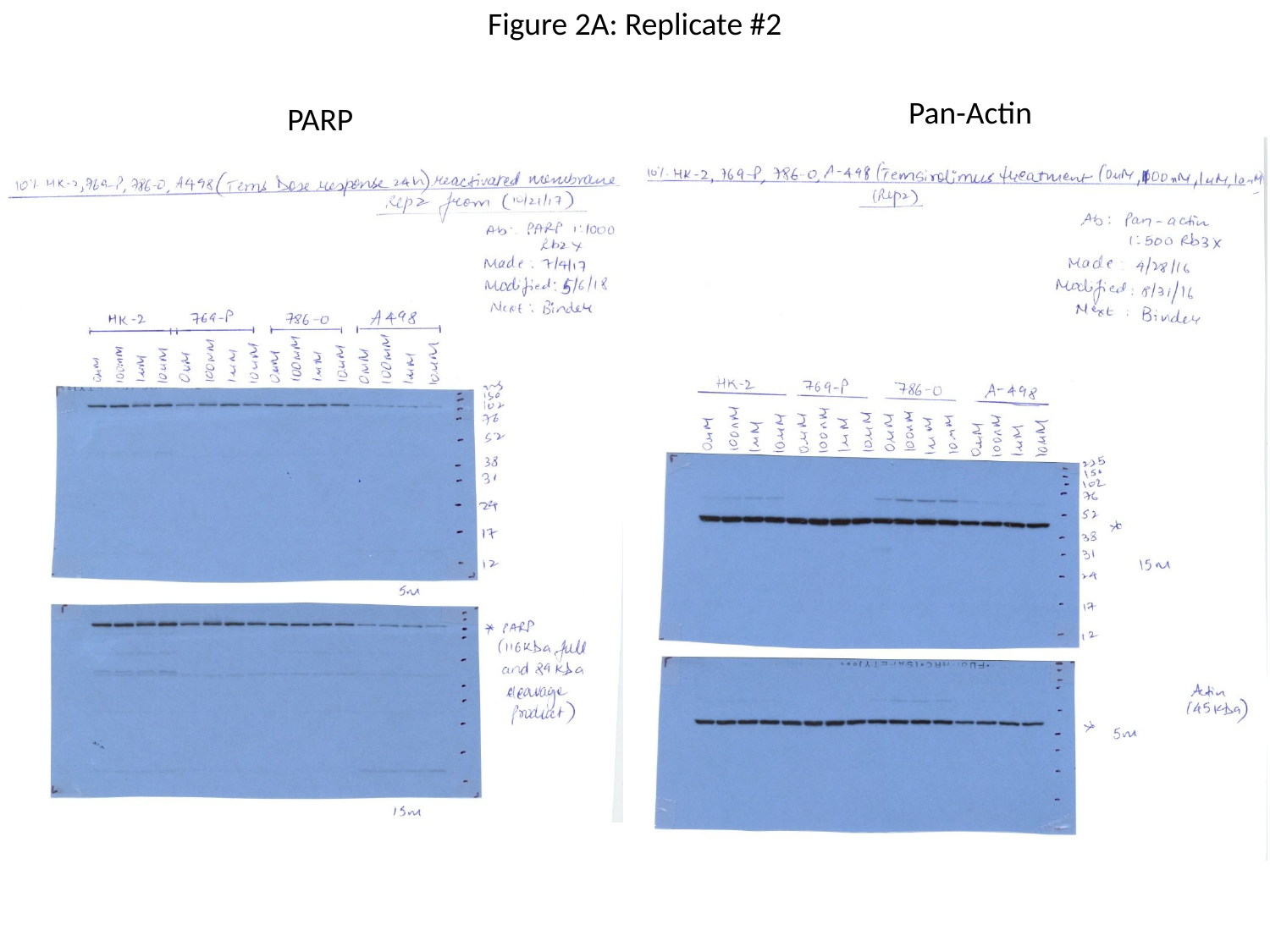

Figure 2A: Replicate #2
Pan-Actin
PARP

## Slide 11
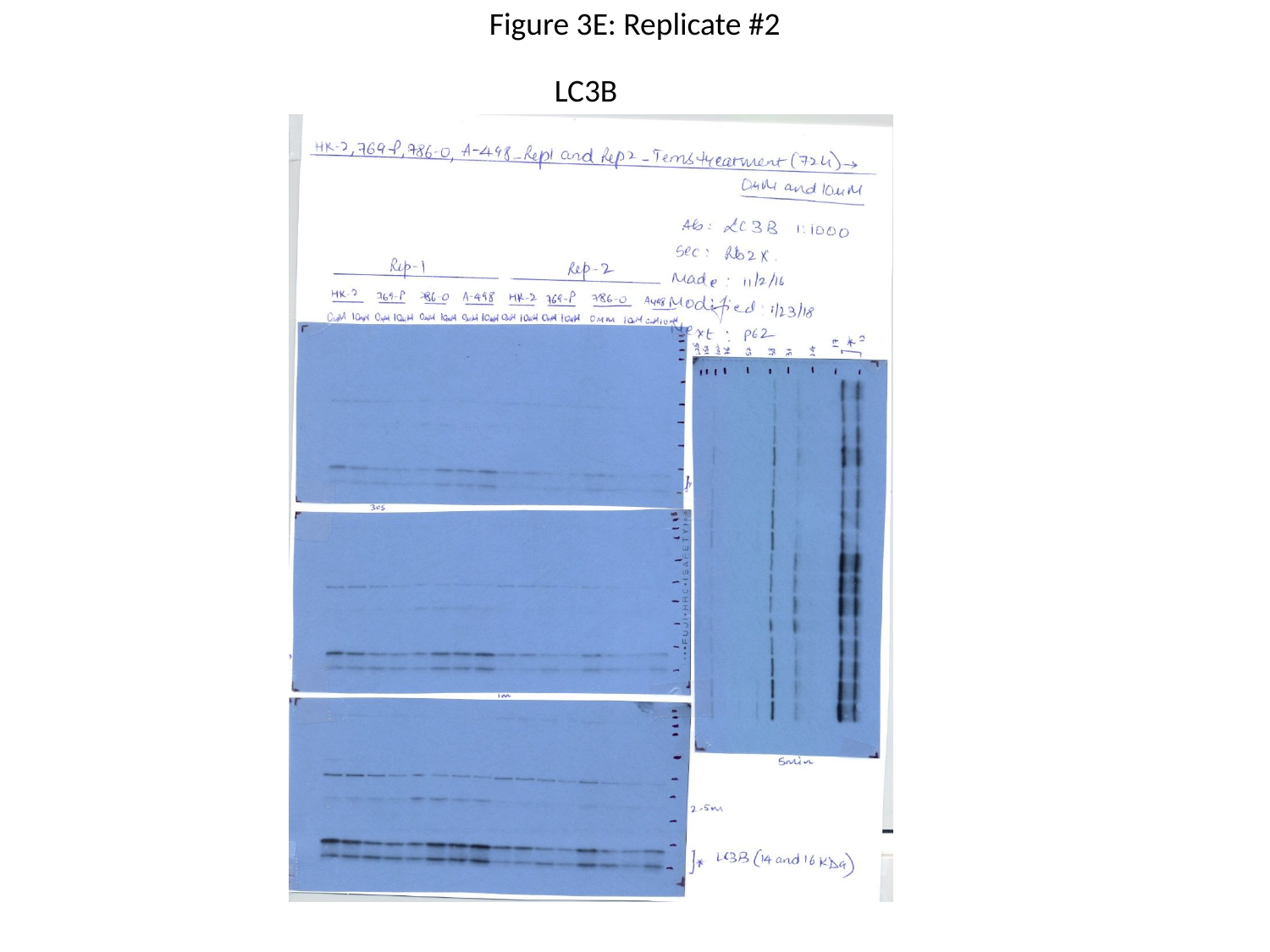

Figure 3E: Replicate #2
LC3B

## Slide 12
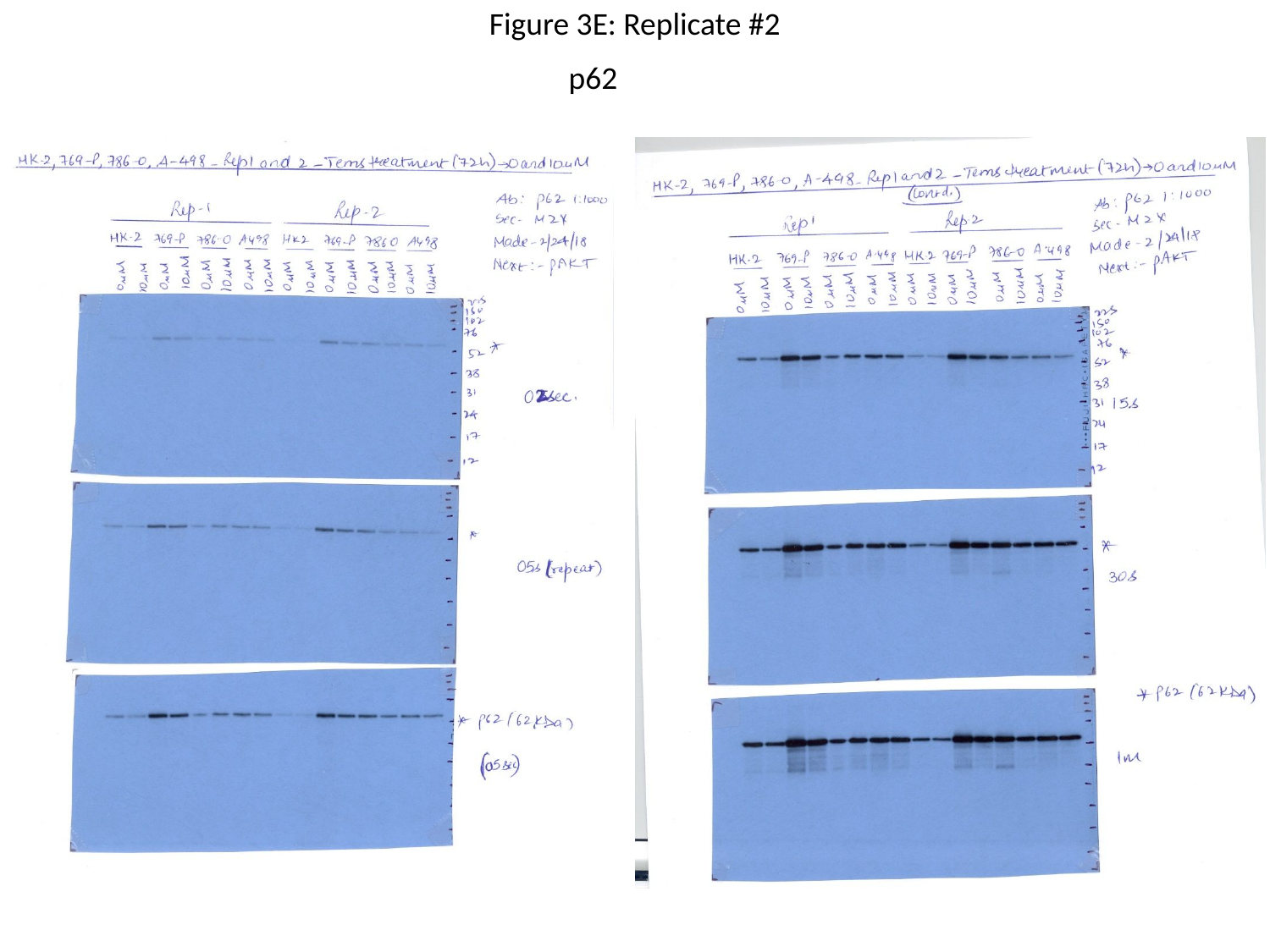

Figure 3E: Replicate #2
p62

## Slide 13
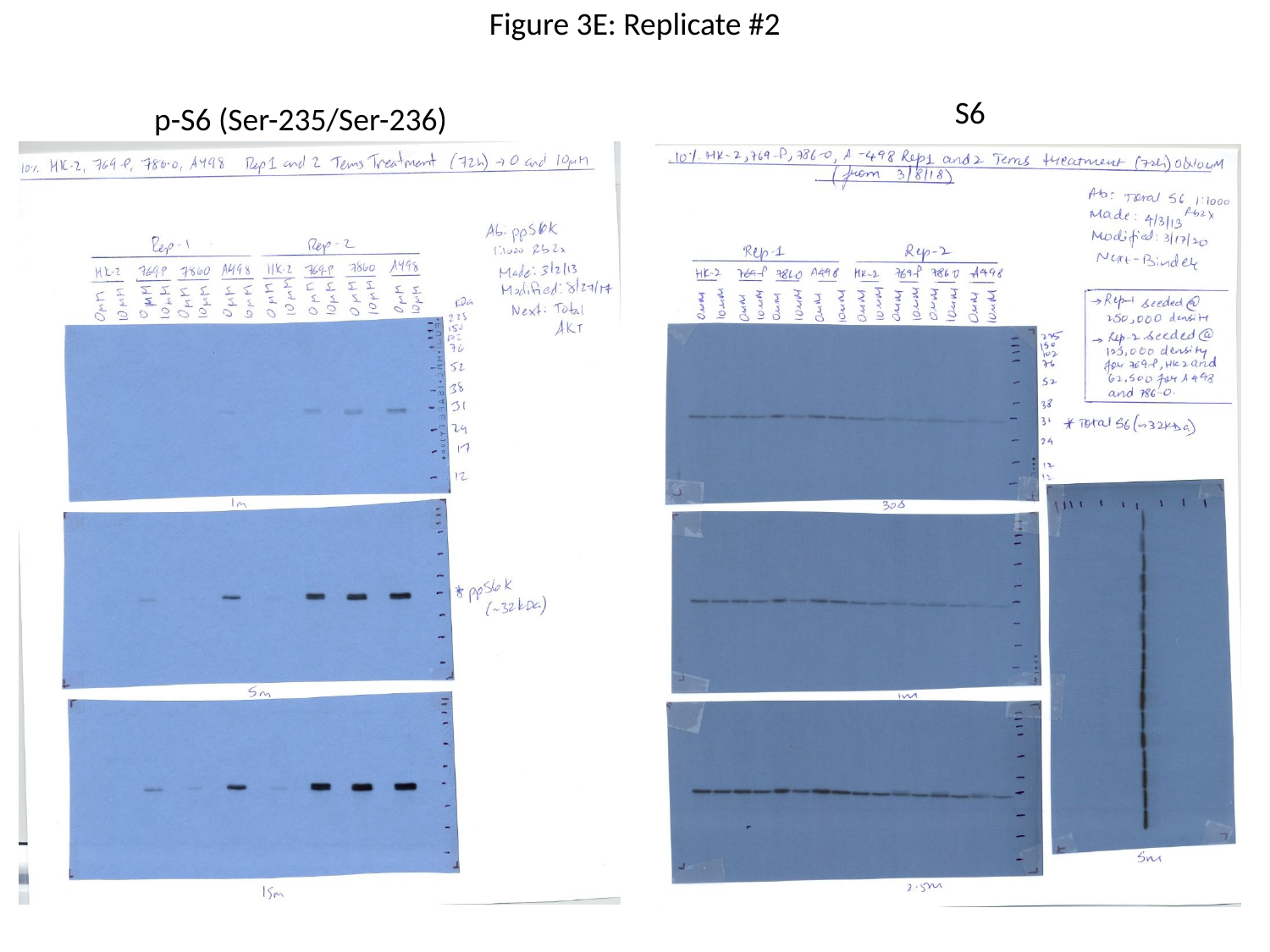

Figure 3E: Replicate #2
S6
p-S6 (Ser-235/Ser-236)

## Slide 14
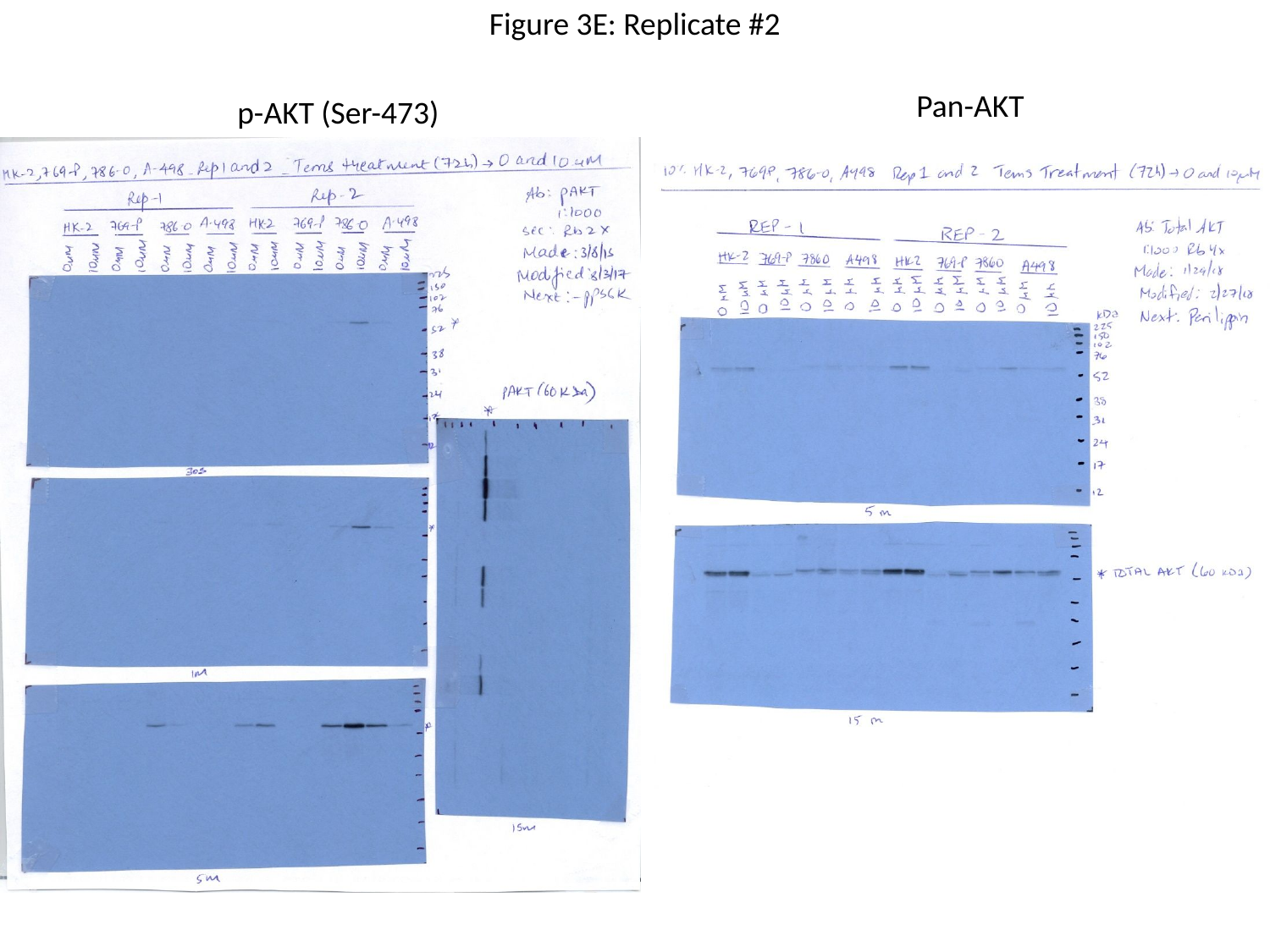

Figure 3E: Replicate #2
Pan-AKT
p-AKT (Ser-473)

## Slide 15
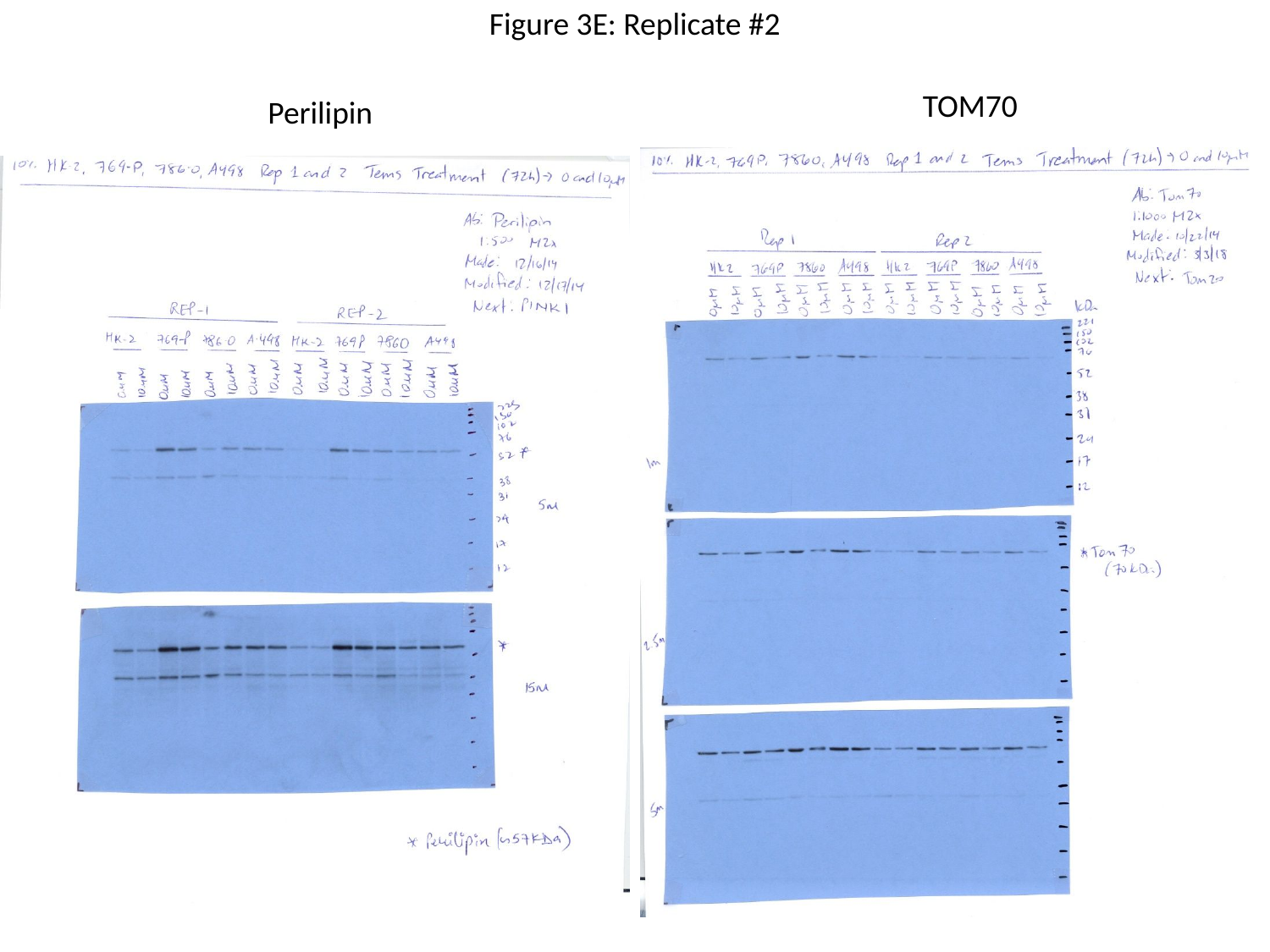

Figure 3E: Replicate #2
TOM70
Perilipin

## Slide 16
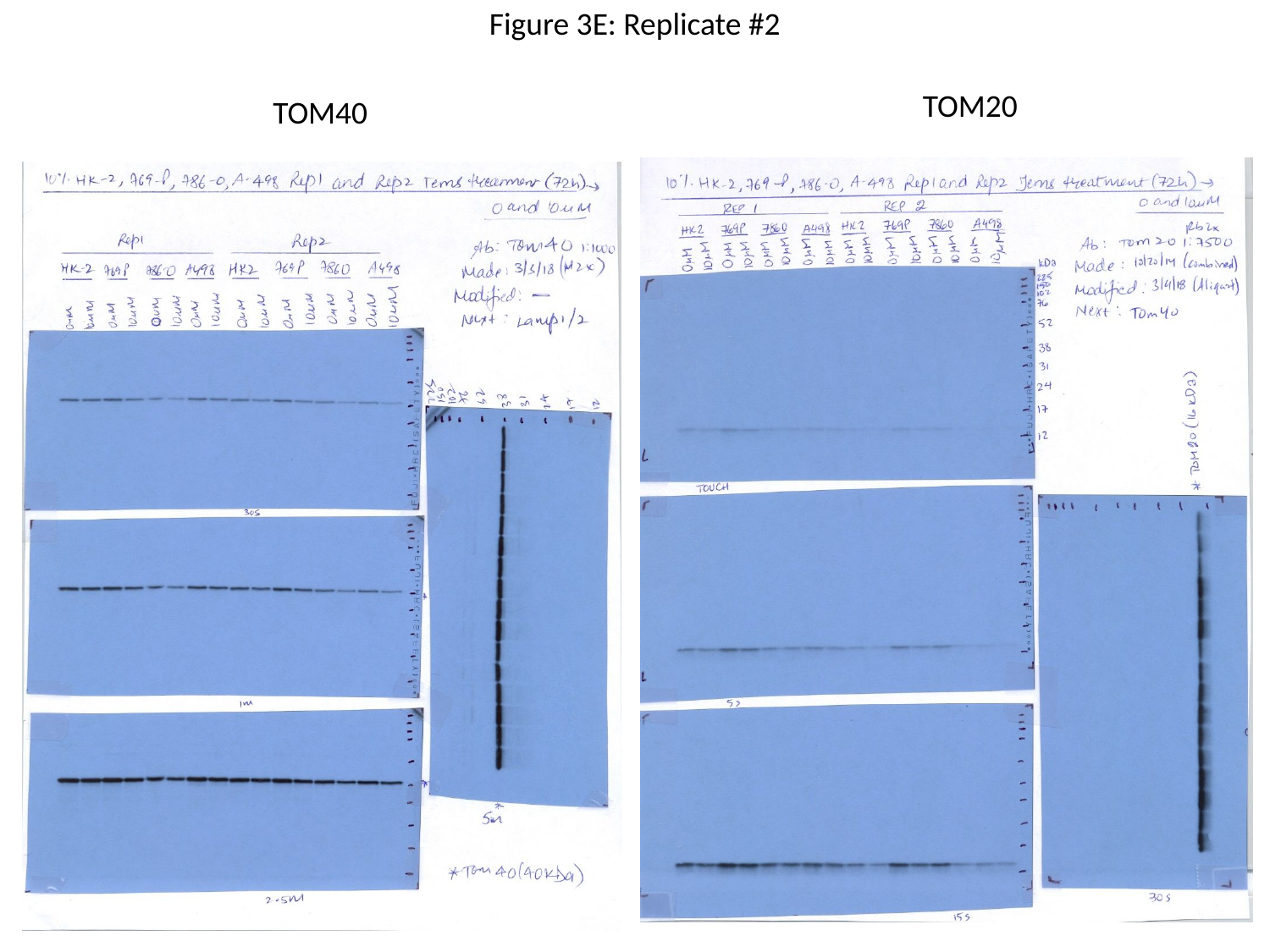

Figure 3E: Replicate #2
TOM20
TOM40

## Slide 17
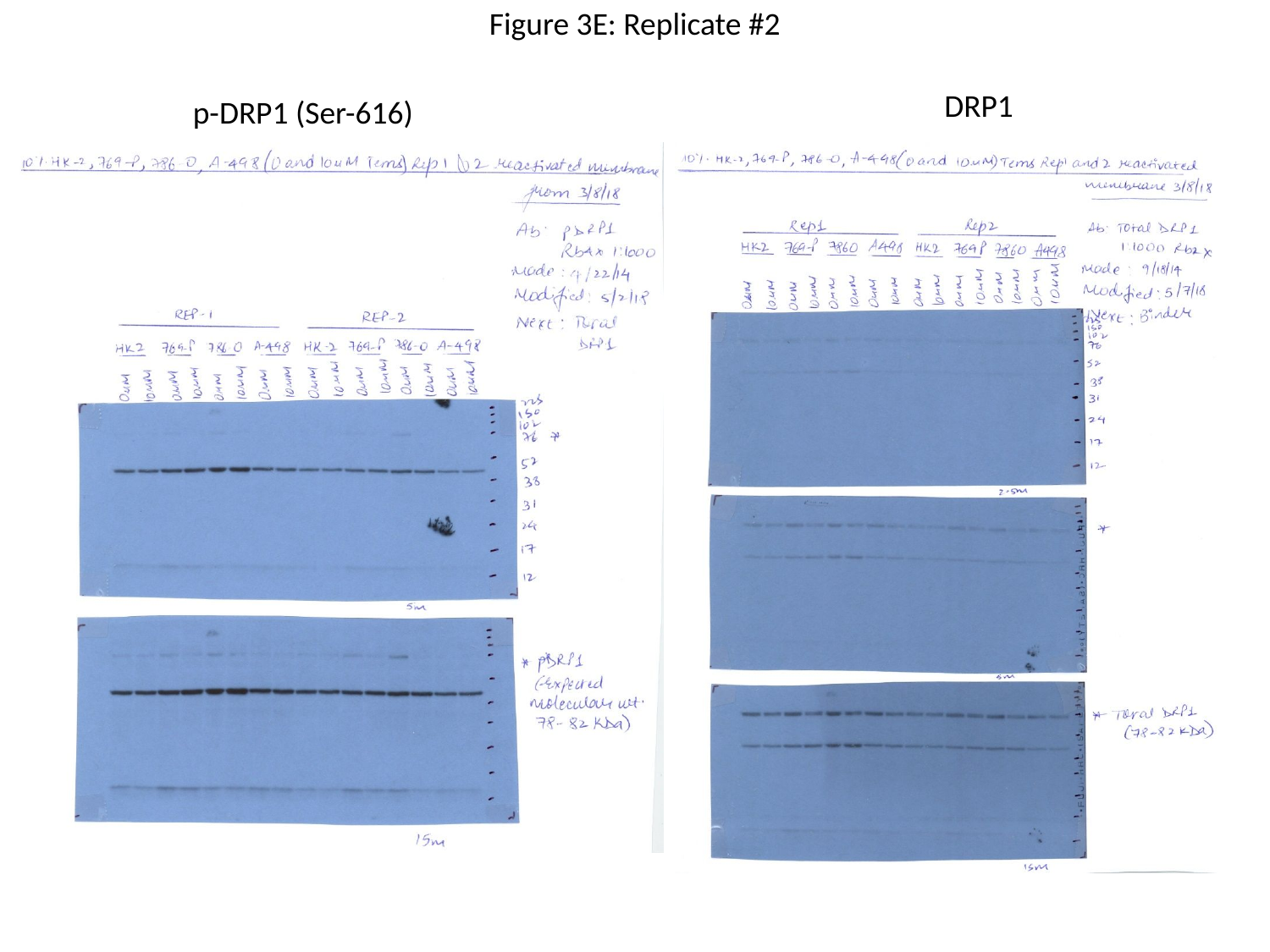

Figure 3E: Replicate #2
DRP1
p-DRP1 (Ser-616)

## Slide 18
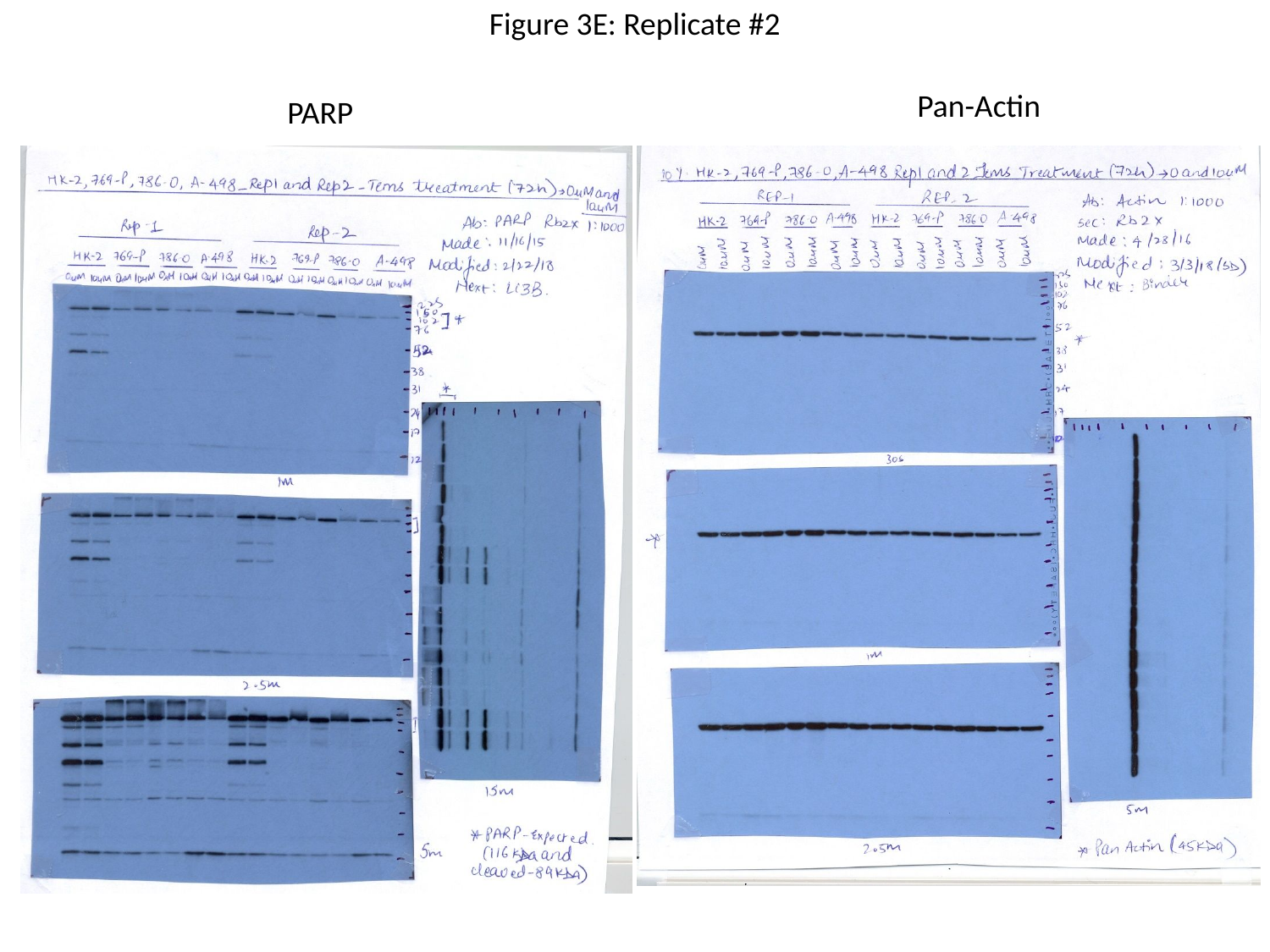

Figure 3E: Replicate #2
Pan-Actin
PARP

## Slide 19
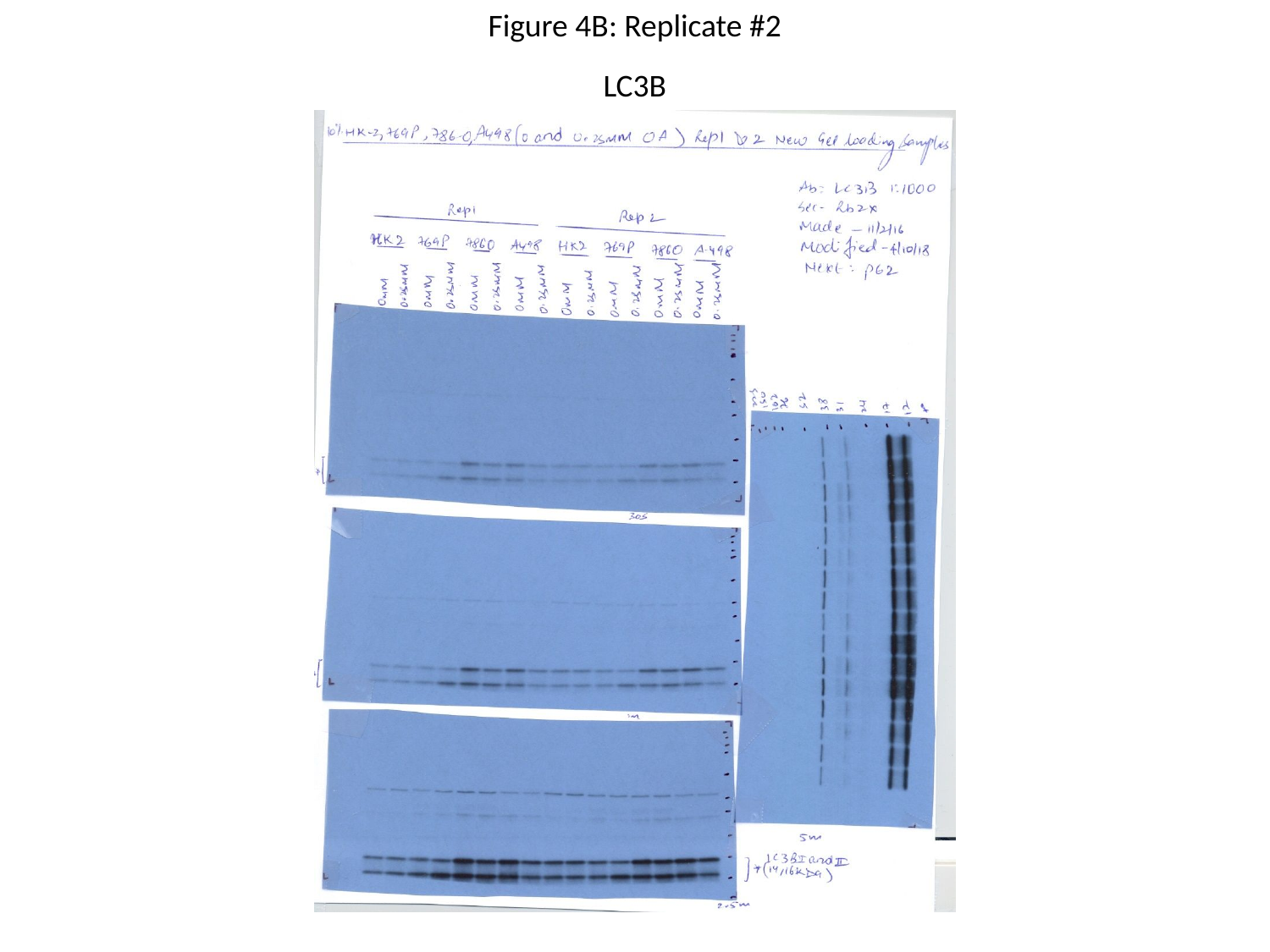

Figure 4B: Replicate #2
LC3B

## Slide 20
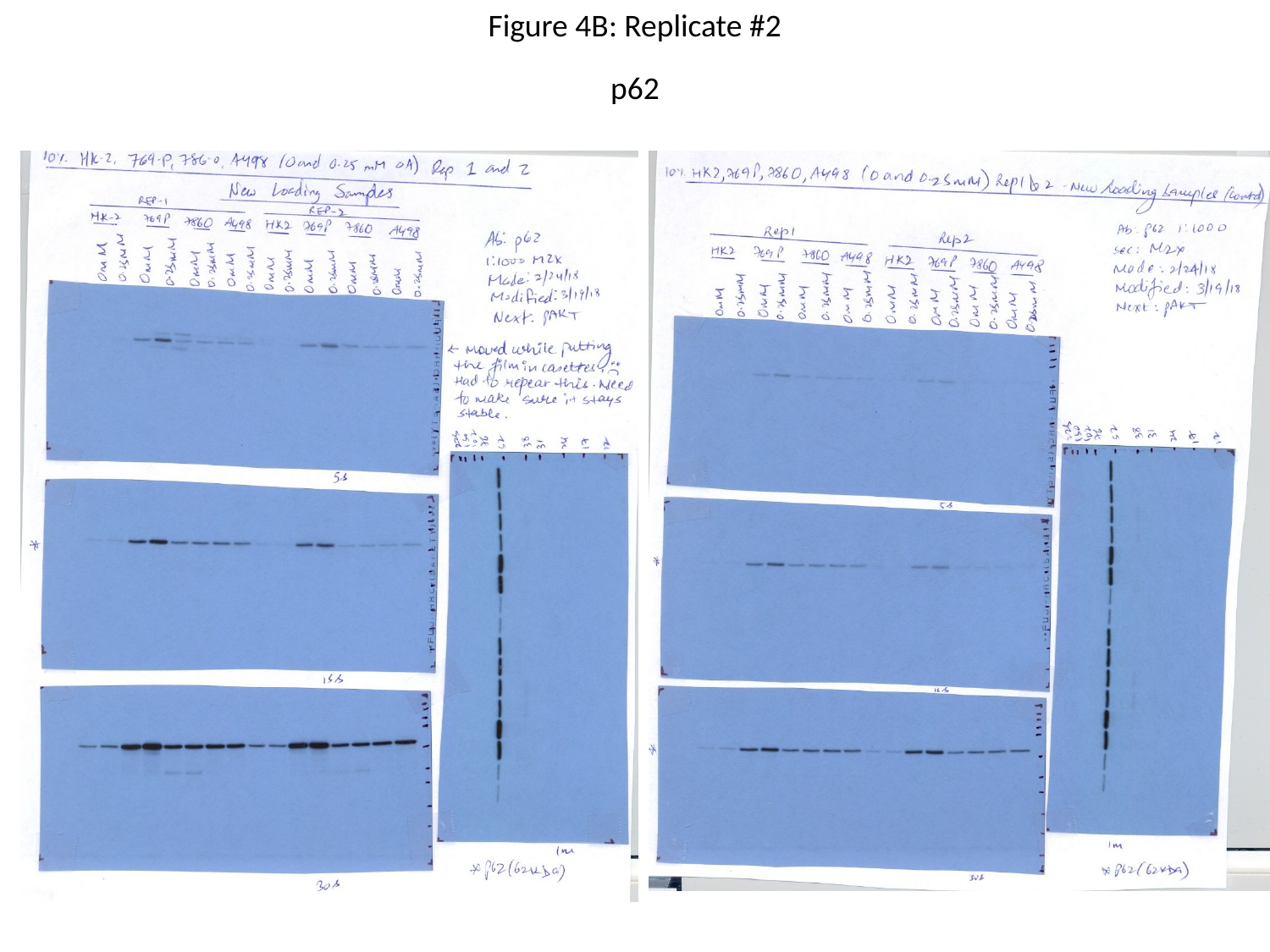

Figure 4B: Replicate #2
p62

## Slide 21
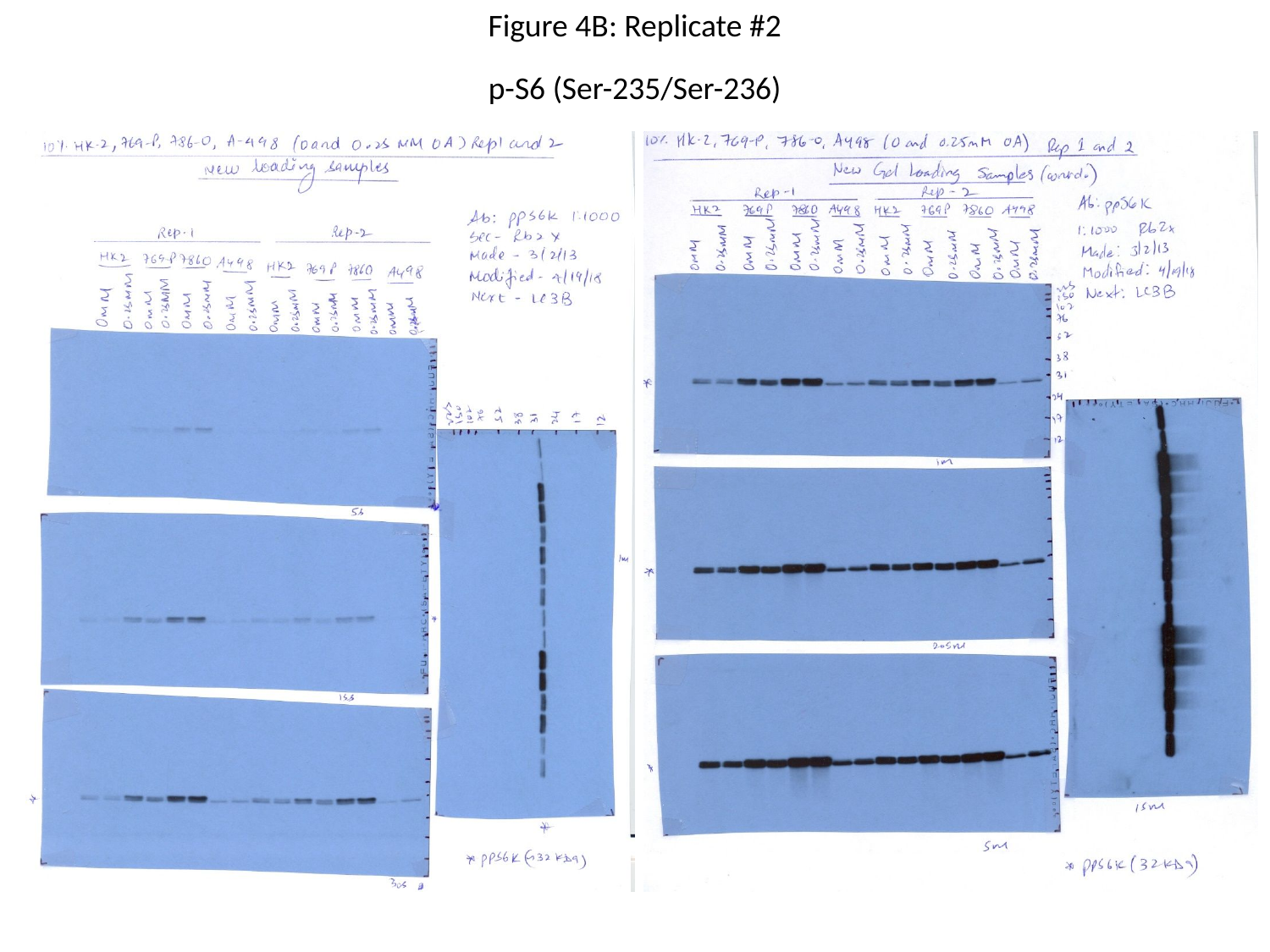

Figure 4B: Replicate #2
p-S6 (Ser-235/Ser-236)

## Slide 22
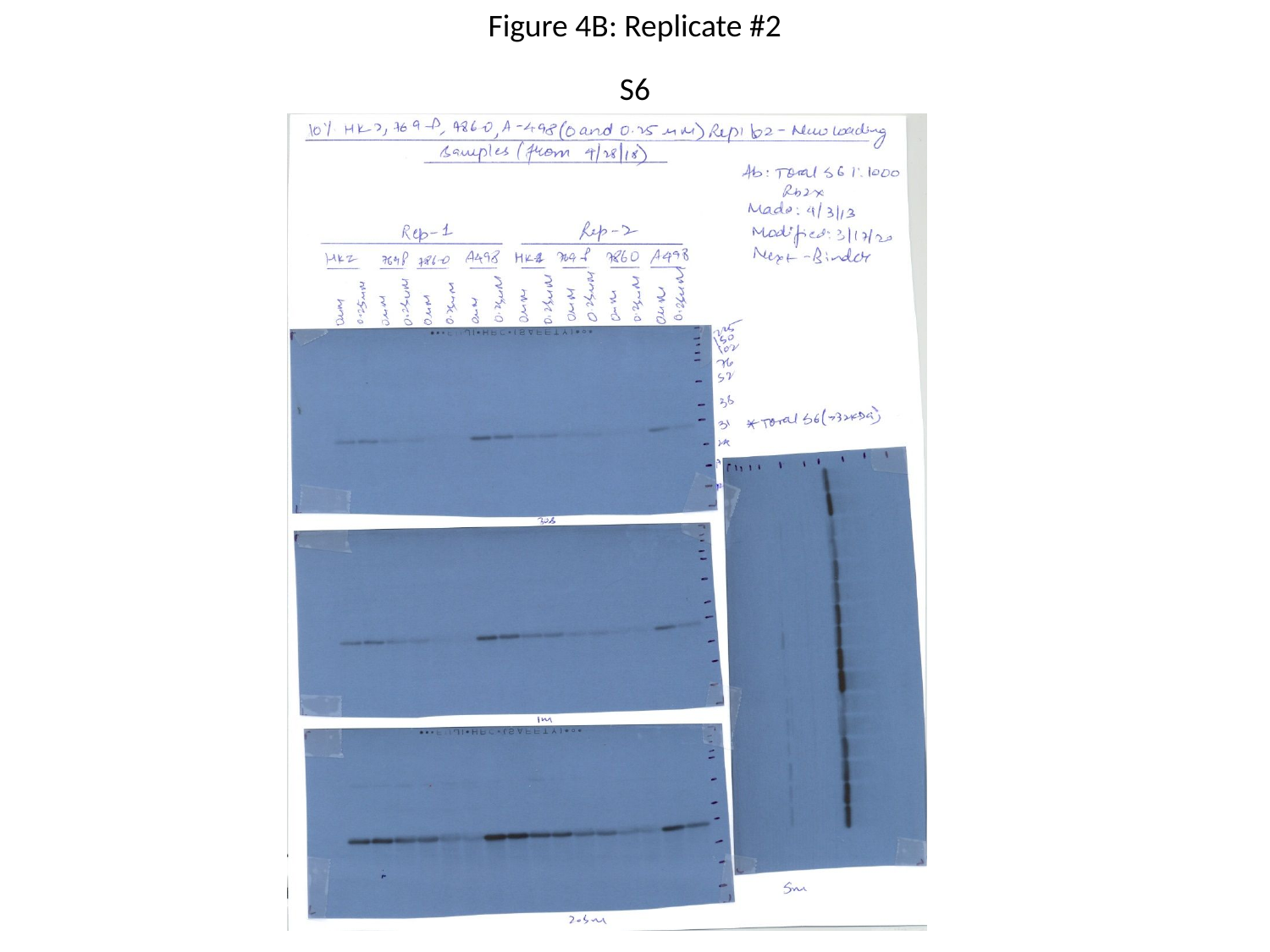

Figure 4B: Replicate #2
S6

## Slide 23
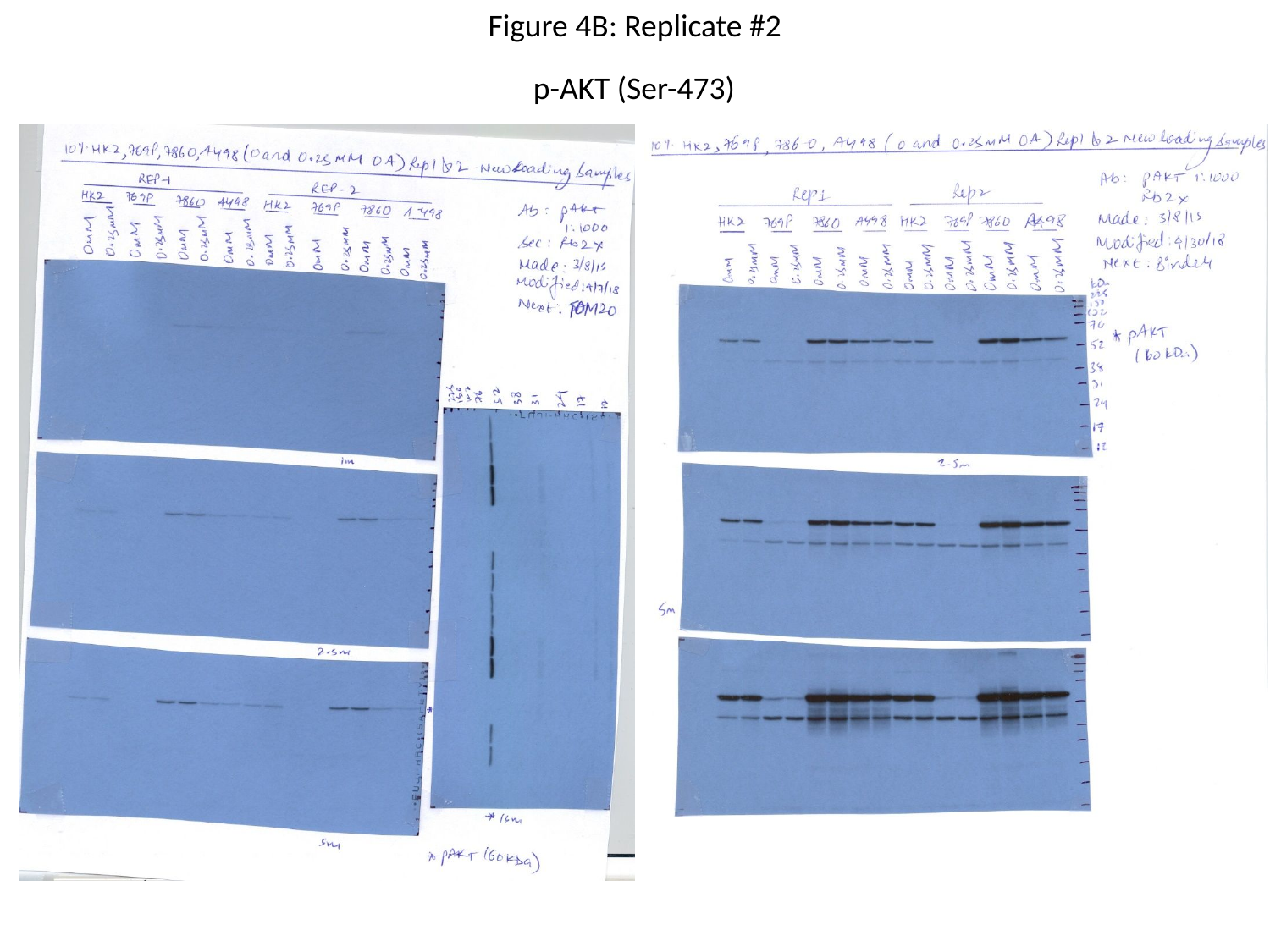

Figure 4B: Replicate #2
p-AKT (Ser-473)

## Slide 24
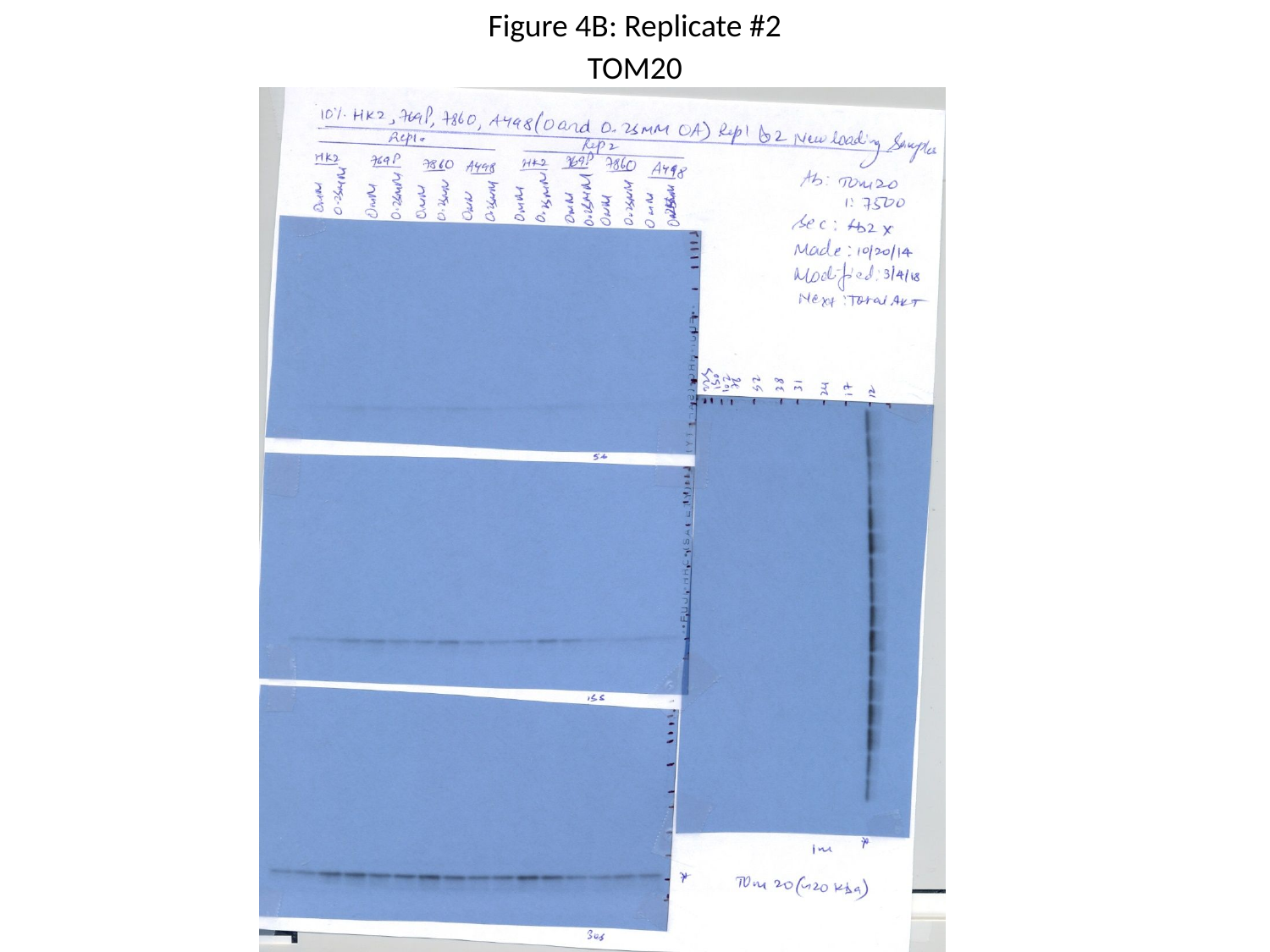

Figure 4B: Replicate #2
TOM20

## Slide 25
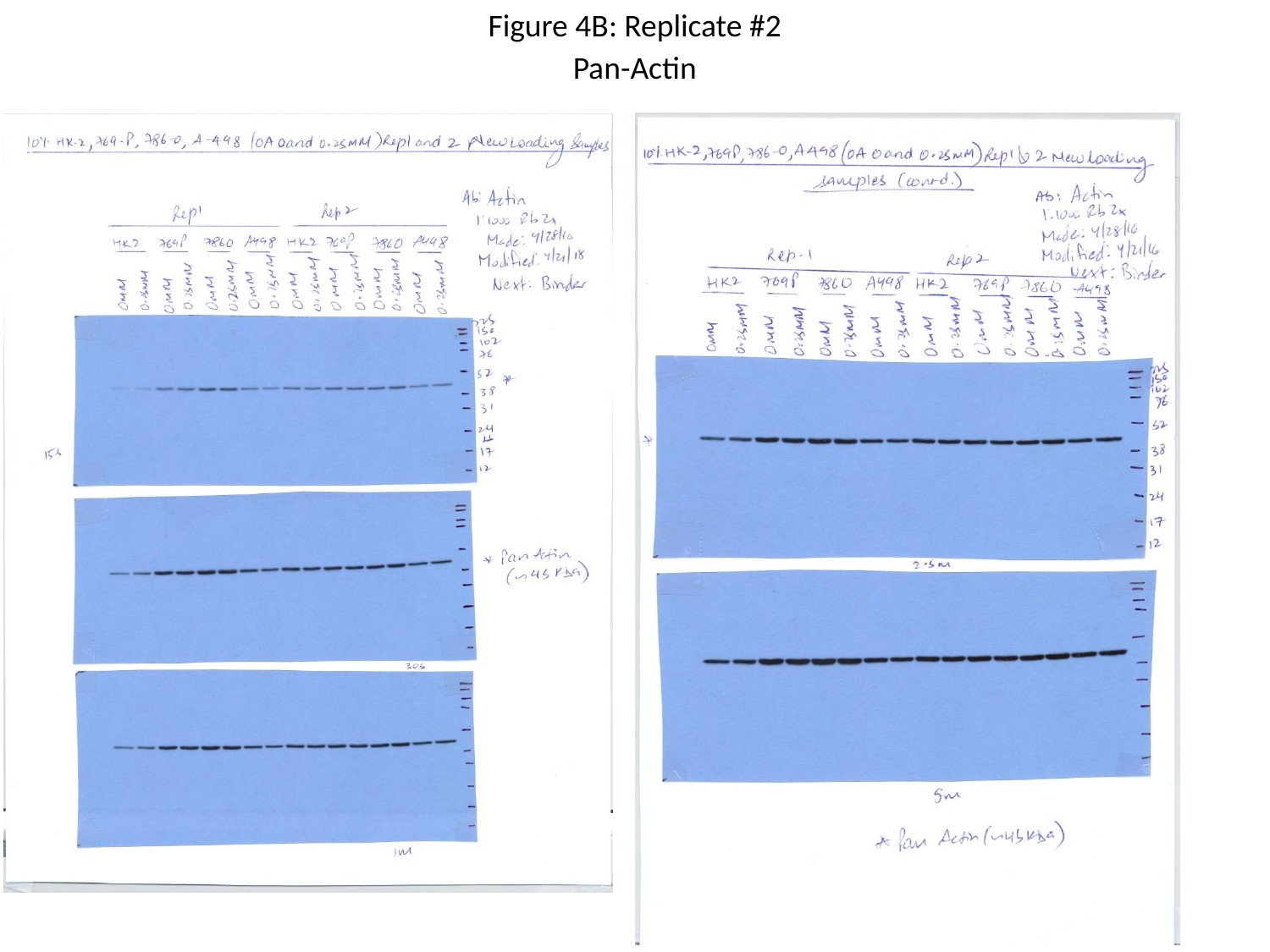

Figure 4B: Replicate #2
Pan-Actin

## Slide 26
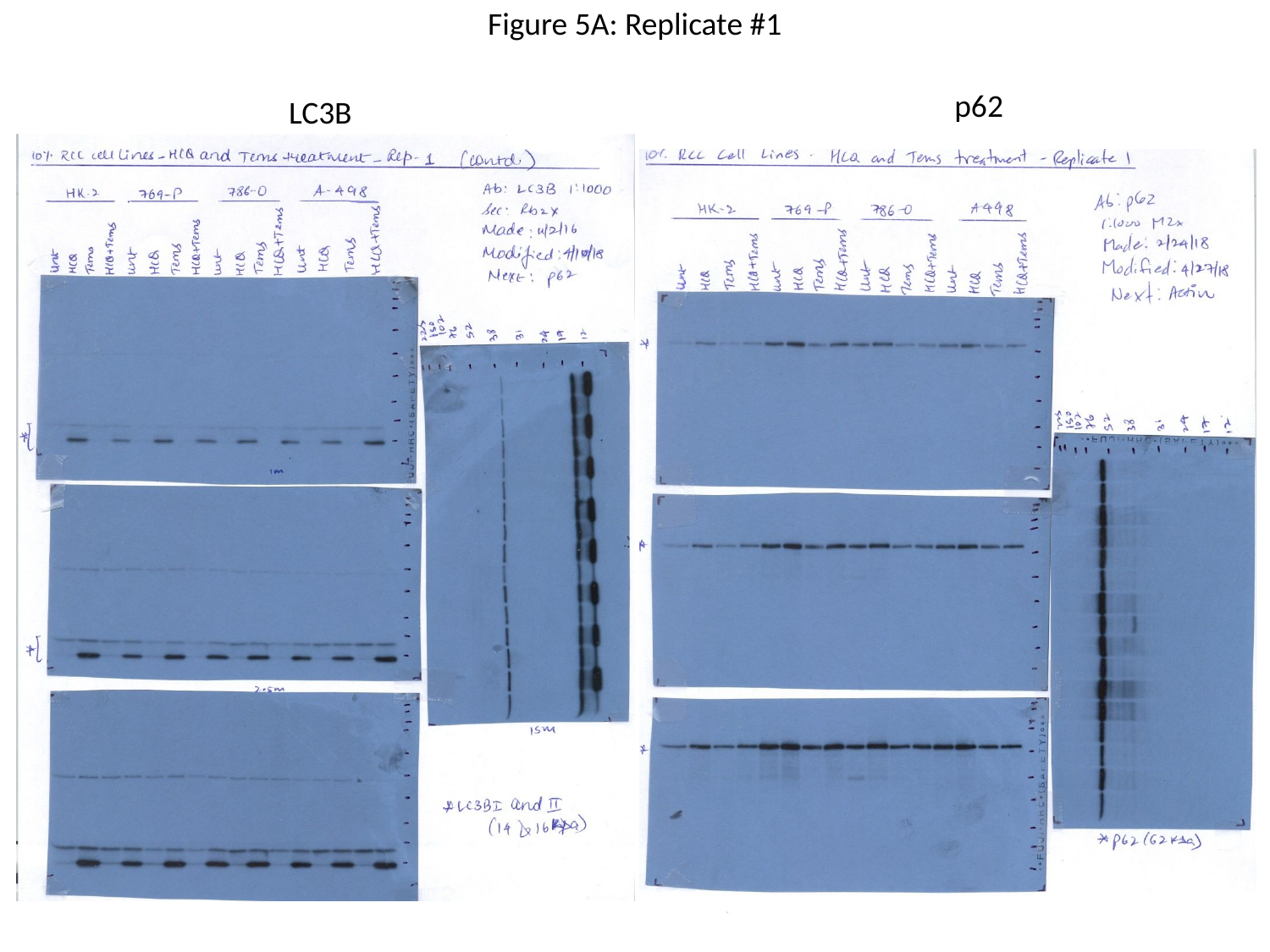

Figure 5A: Replicate #1
p62
LC3B

## Slide 27
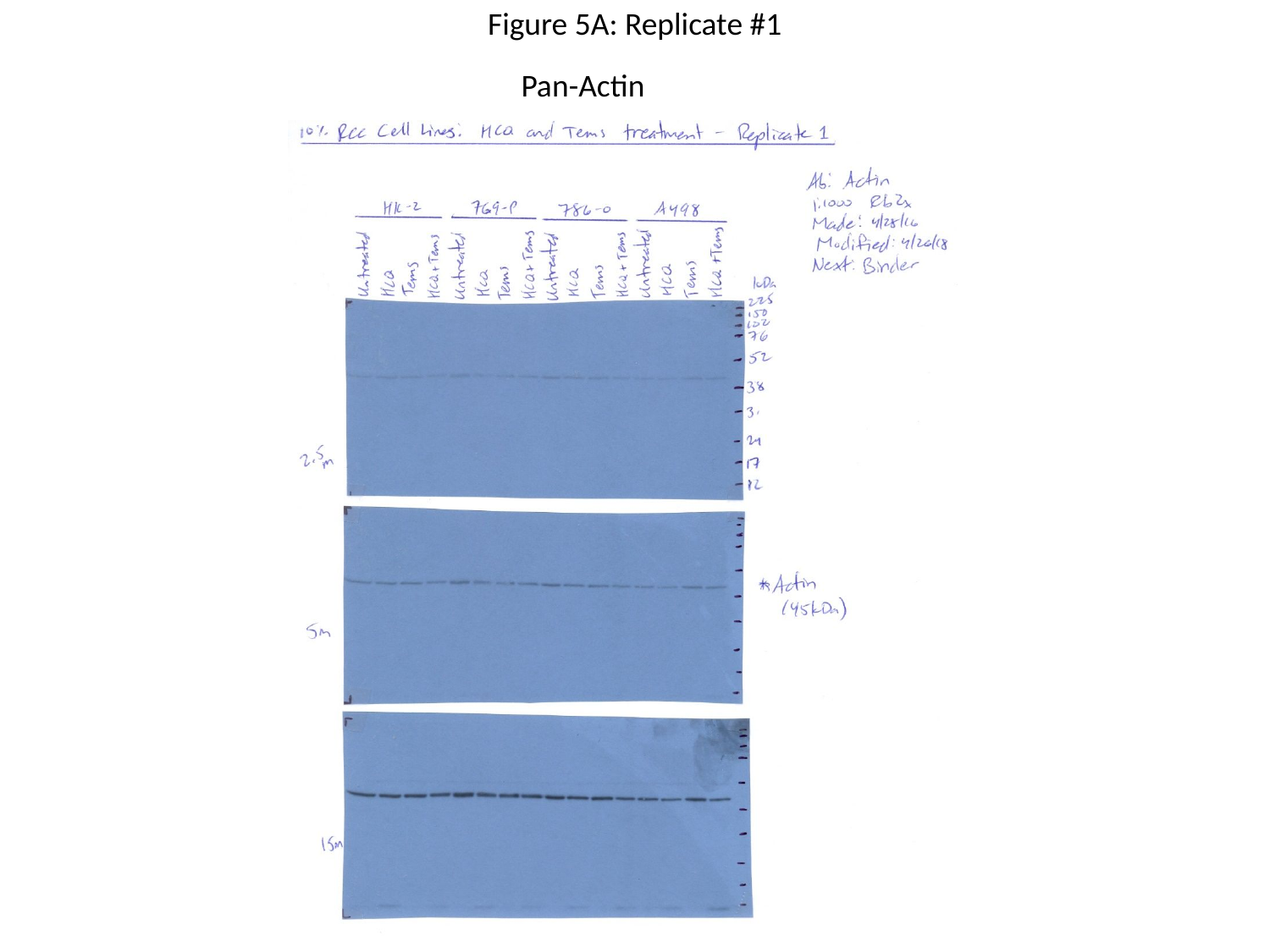

Figure 5A: Replicate #1
Pan-Actin

## Slide 28
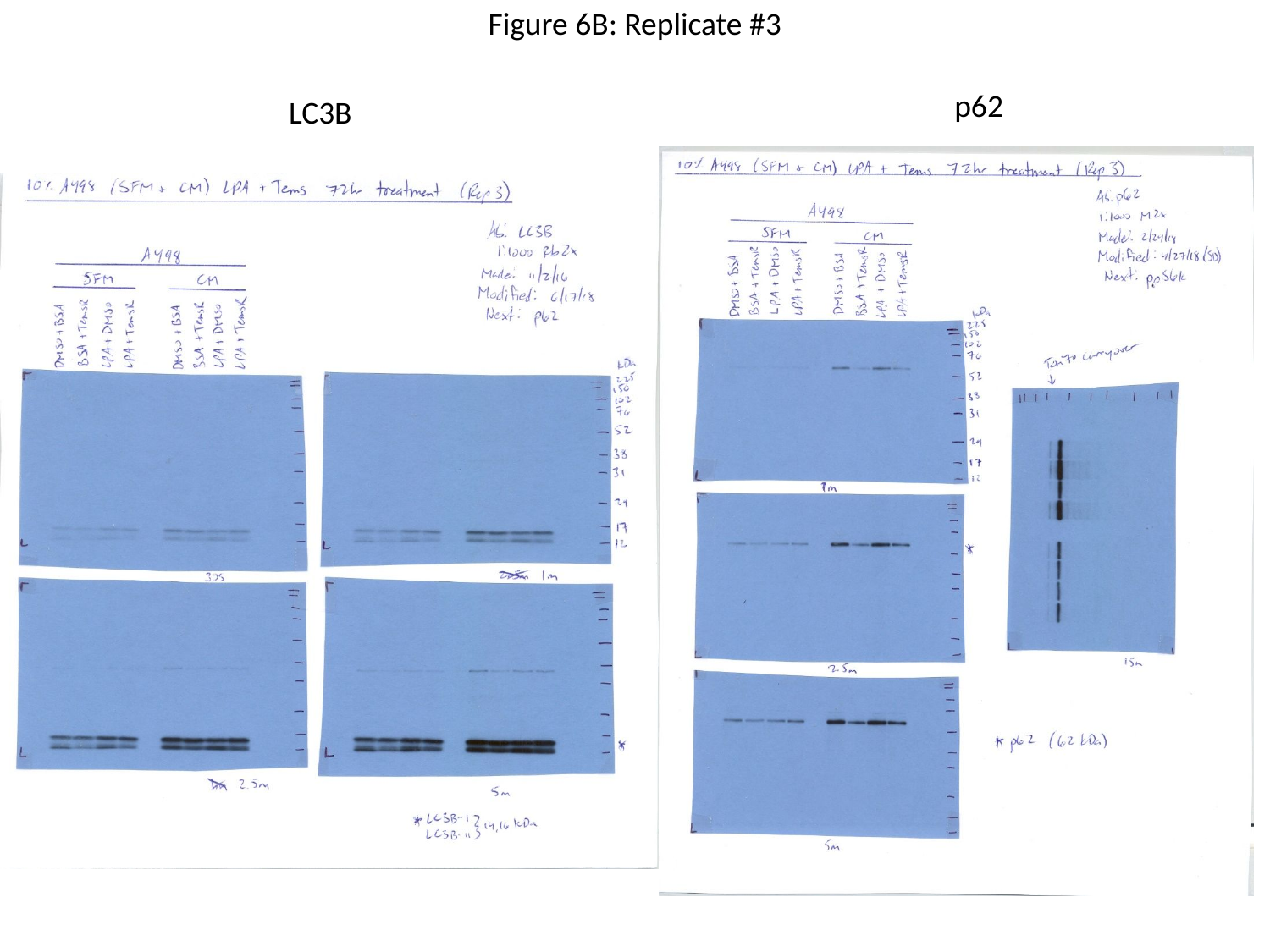

Figure 6B: Replicate #3
p62
LC3B

## Slide 29
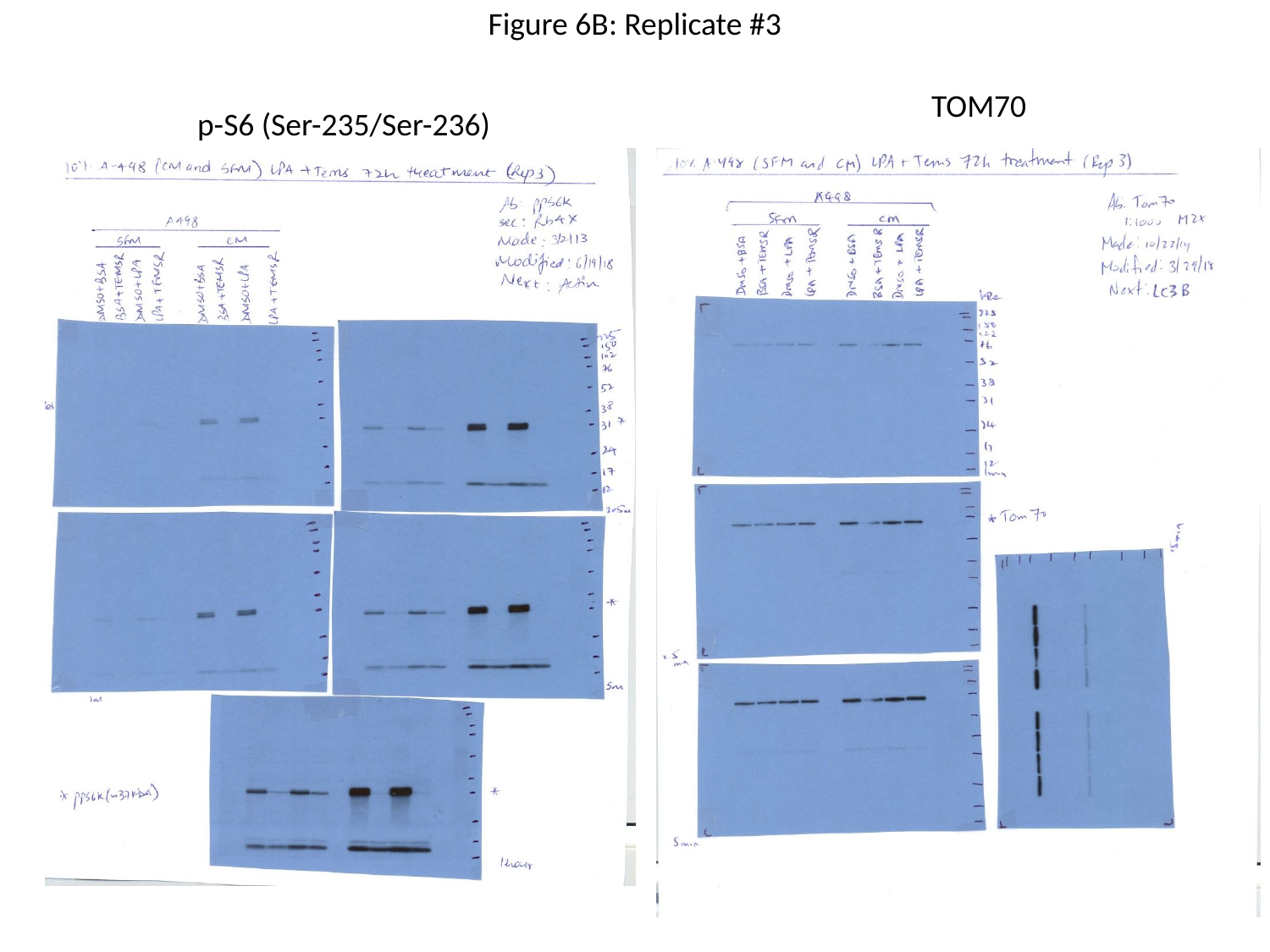

Figure 6B: Replicate #3
TOM70
p-S6 (Ser-235/Ser-236)

## Slide 30
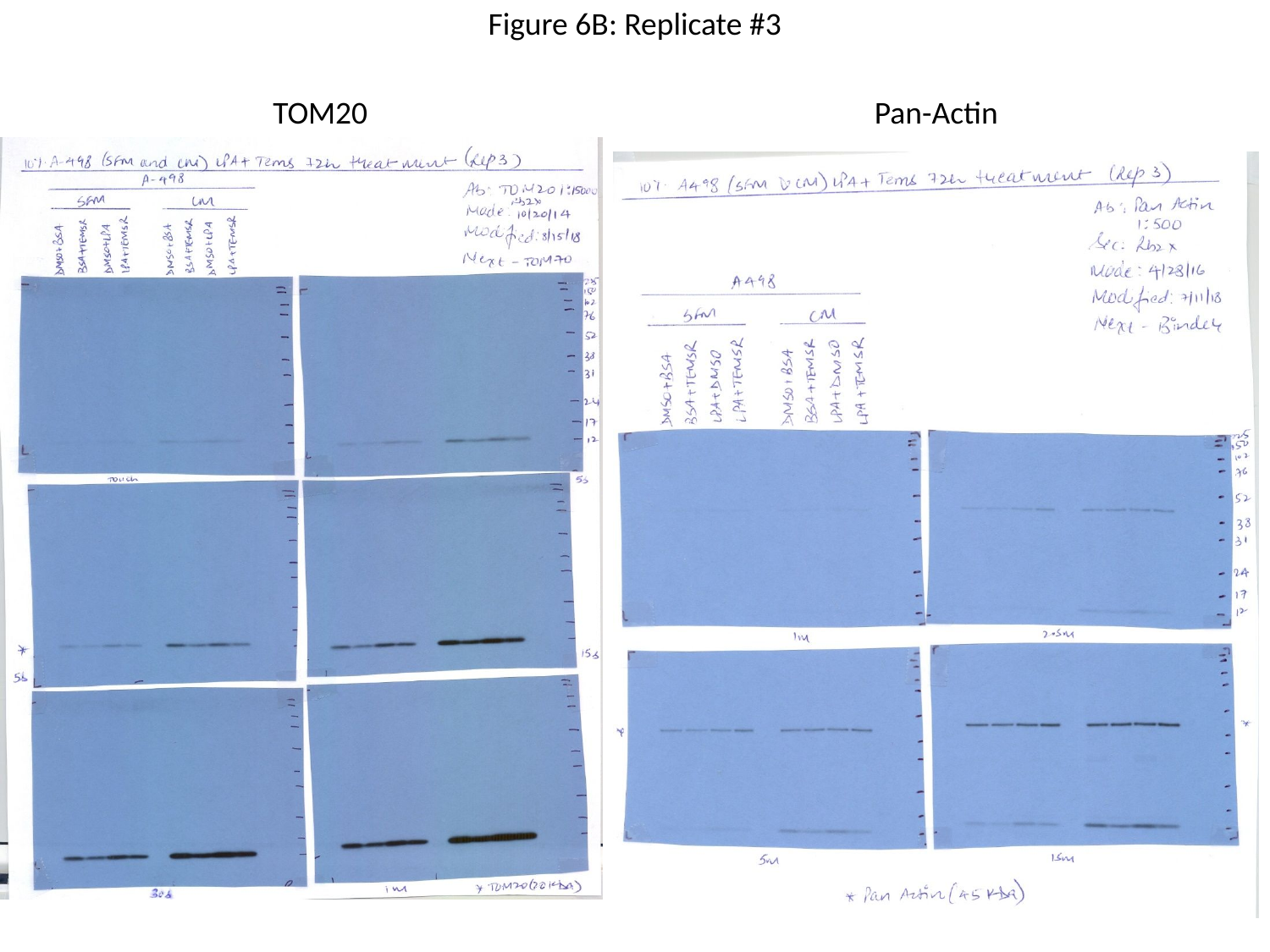

Figure 6B: Replicate #3
TOM20
Pan-Actin

## Slide 31
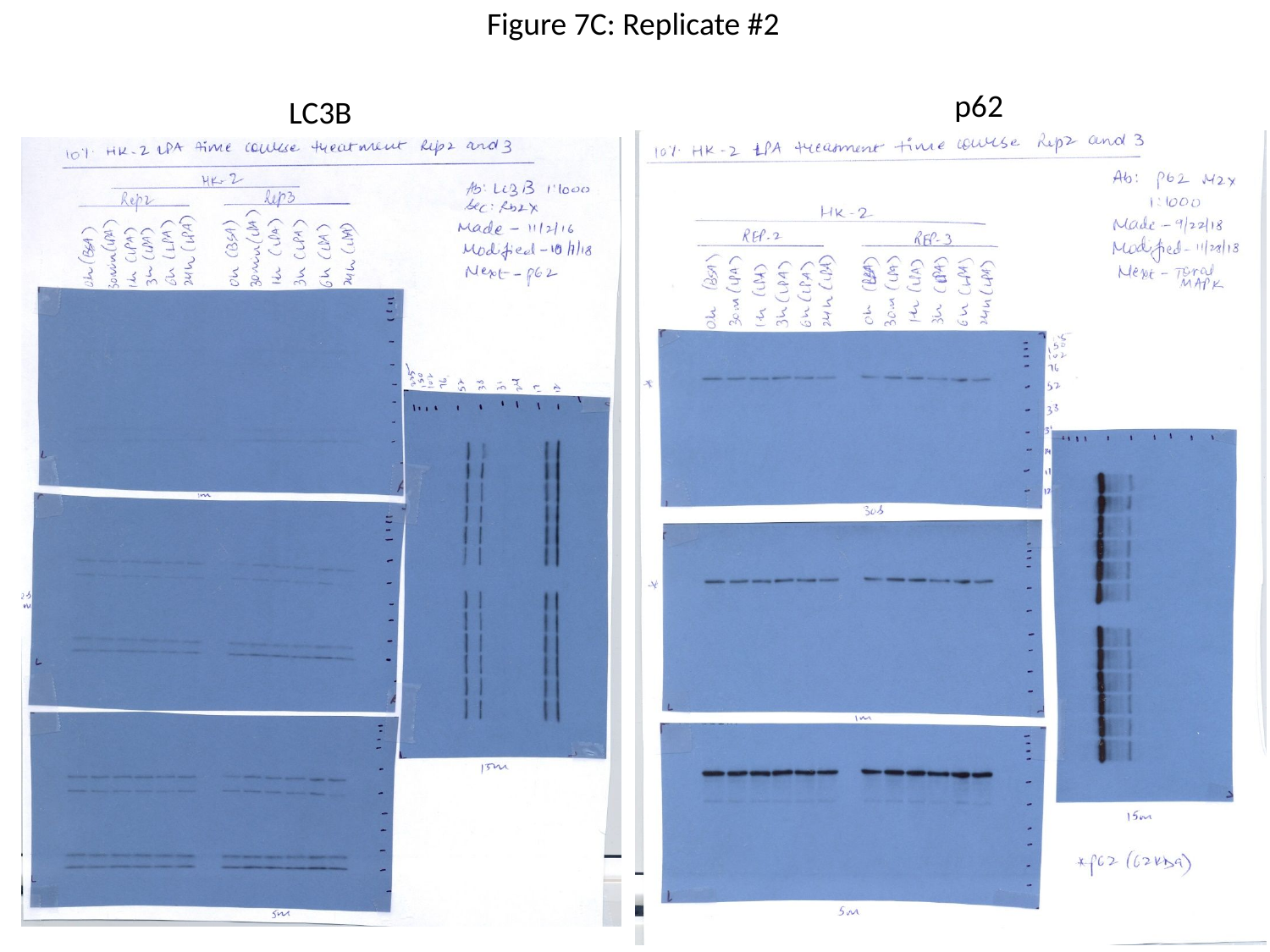

Figure 7C: Replicate #2
p62
LC3B

## Slide 32
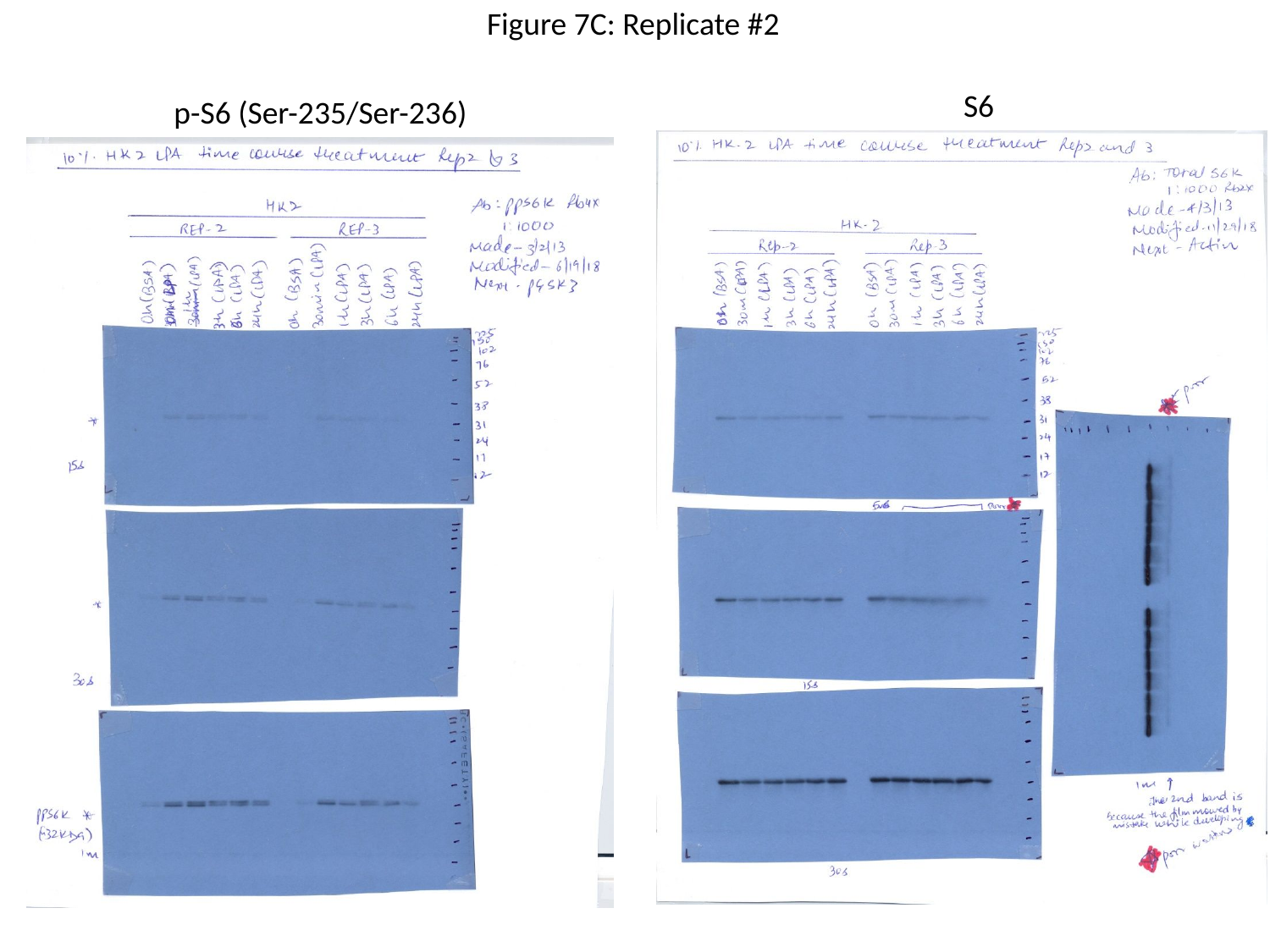

Figure 7C: Replicate #2
S6
p-S6 (Ser-235/Ser-236)

## Slide 33
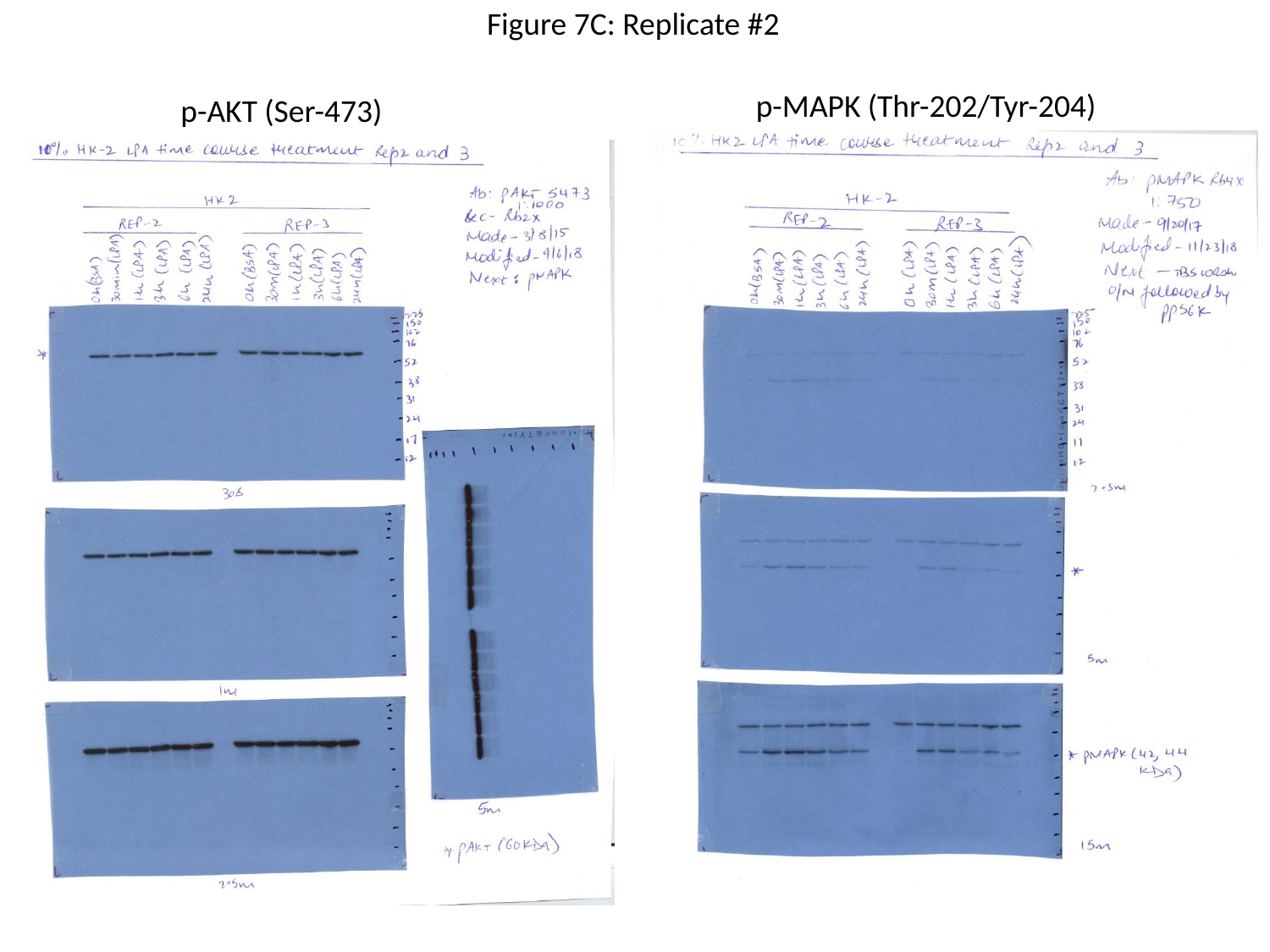

Figure 7C: Replicate #2
p-MAPK (Thr-202/Tyr-204)
p-AKT (Ser-473)

## Slide 34
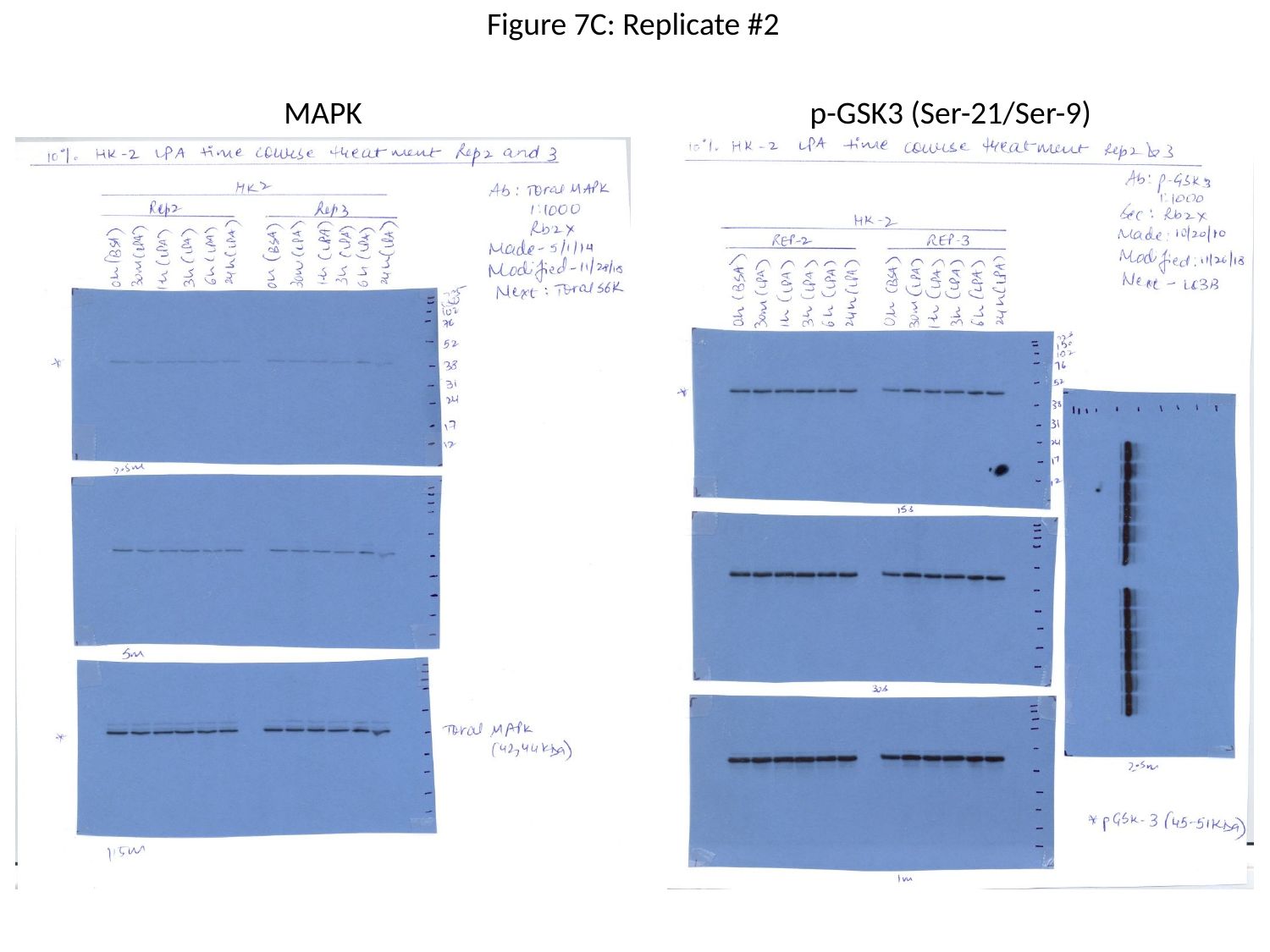

Figure 7C: Replicate #2
MAPK
p-GSK3 (Ser-21/Ser-9)

## Slide 35
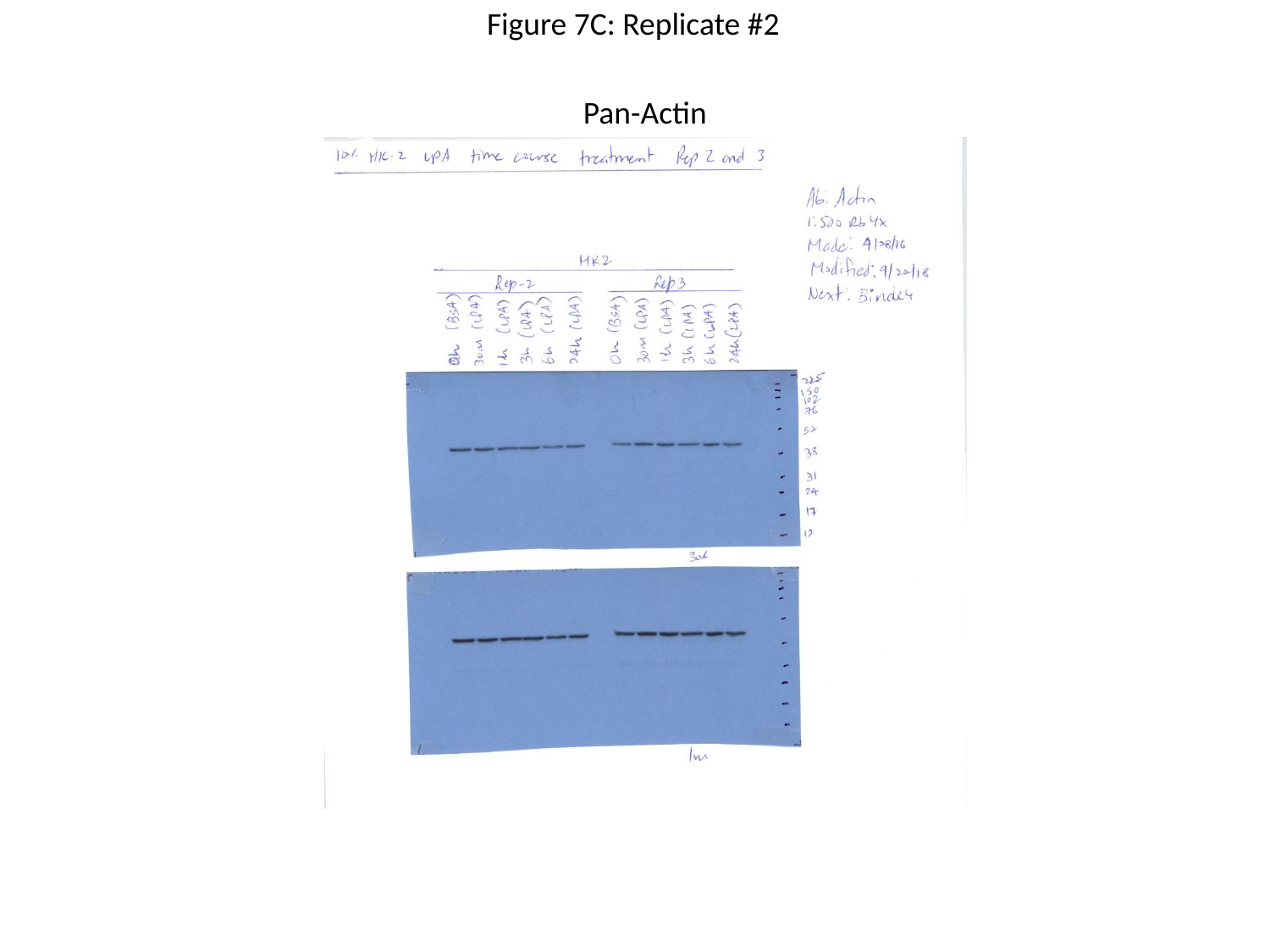

Figure 7C: Replicate #2
Pan-Actin

## Slide 36
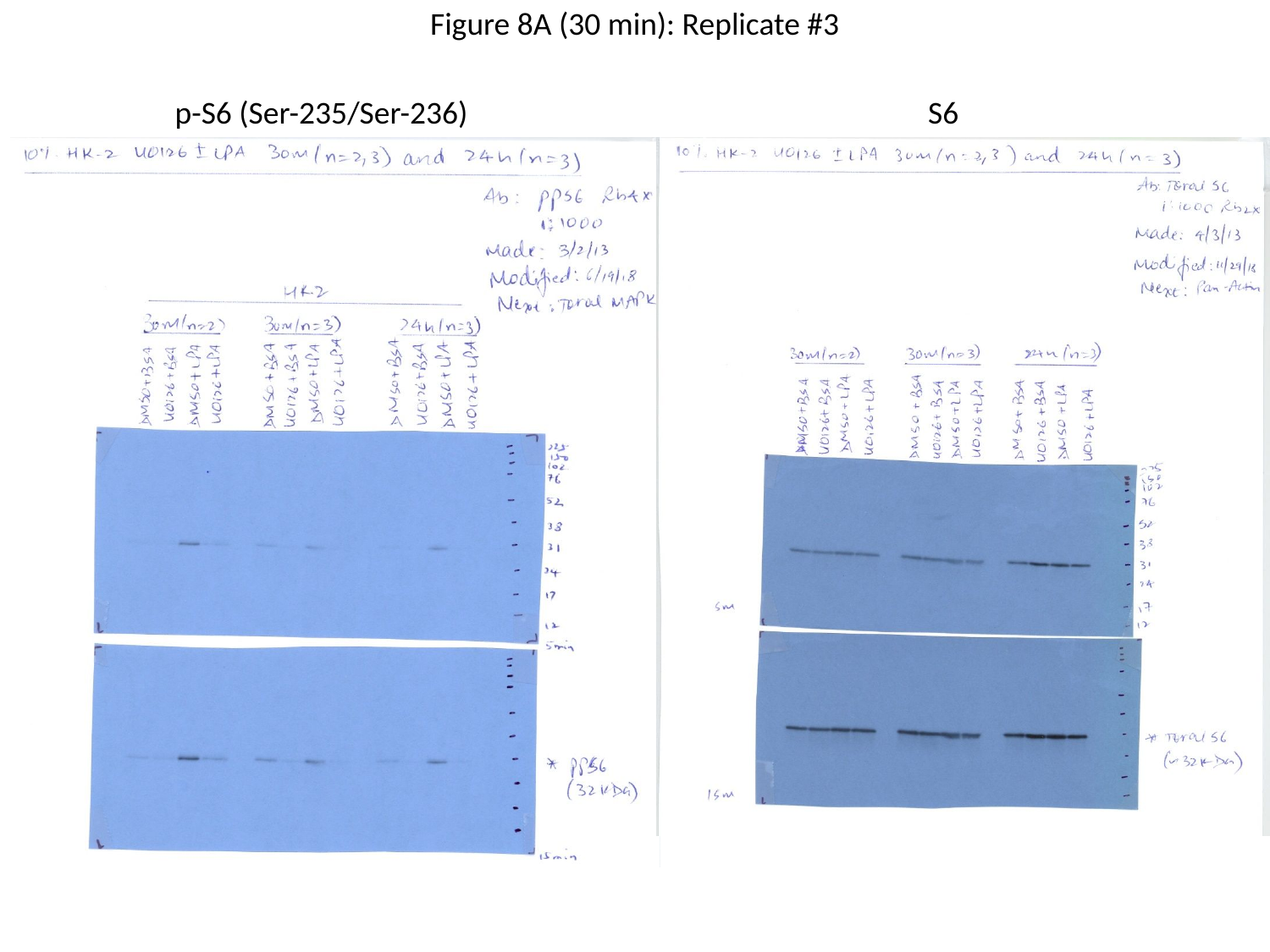

Figure 8A (30 min): Replicate #3
p-S6 (Ser-235/Ser-236)
S6

## Slide 37
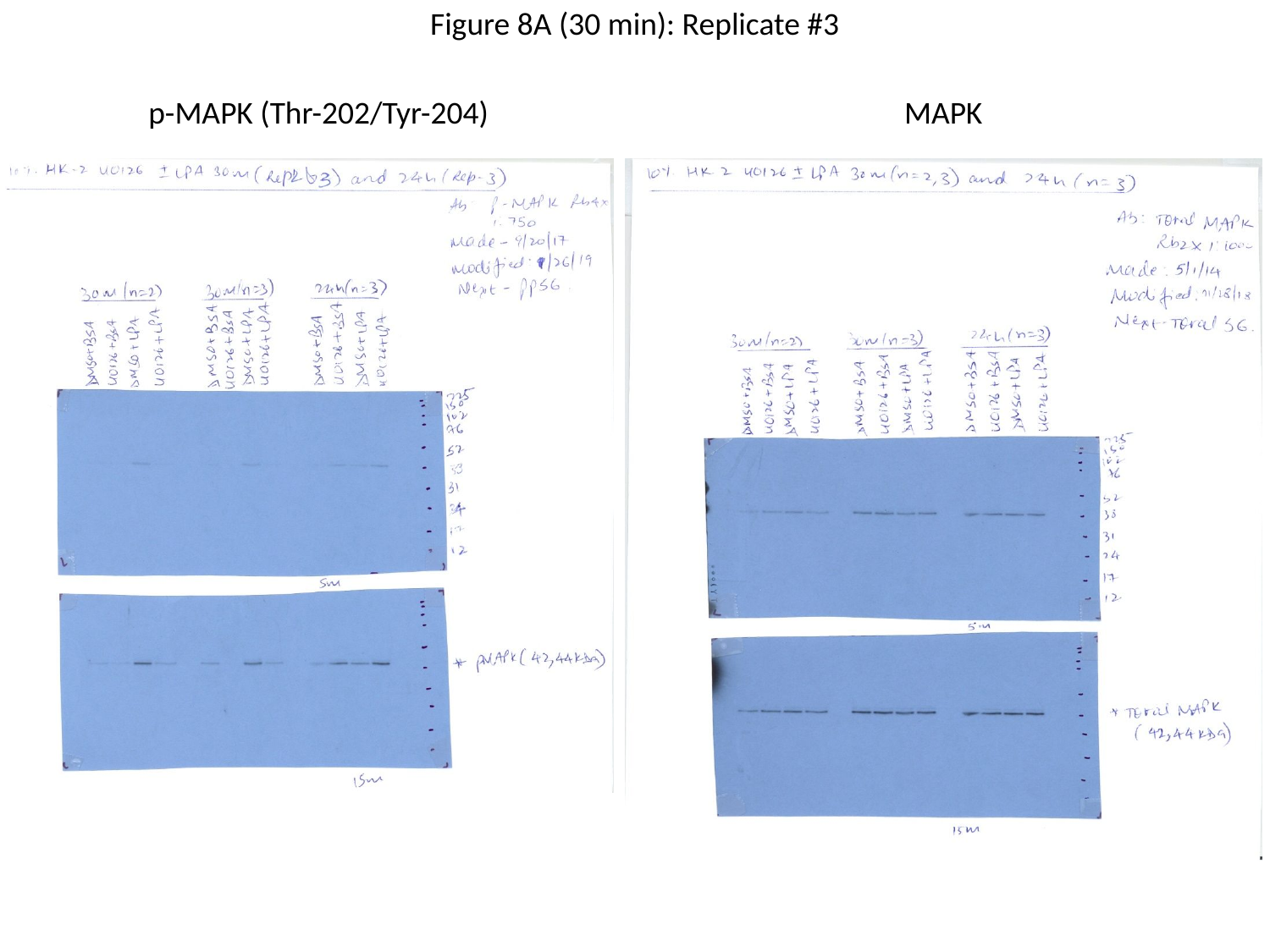

Figure 8A (30 min): Replicate #3
p-MAPK (Thr-202/Tyr-204)
MAPK

## Slide 38
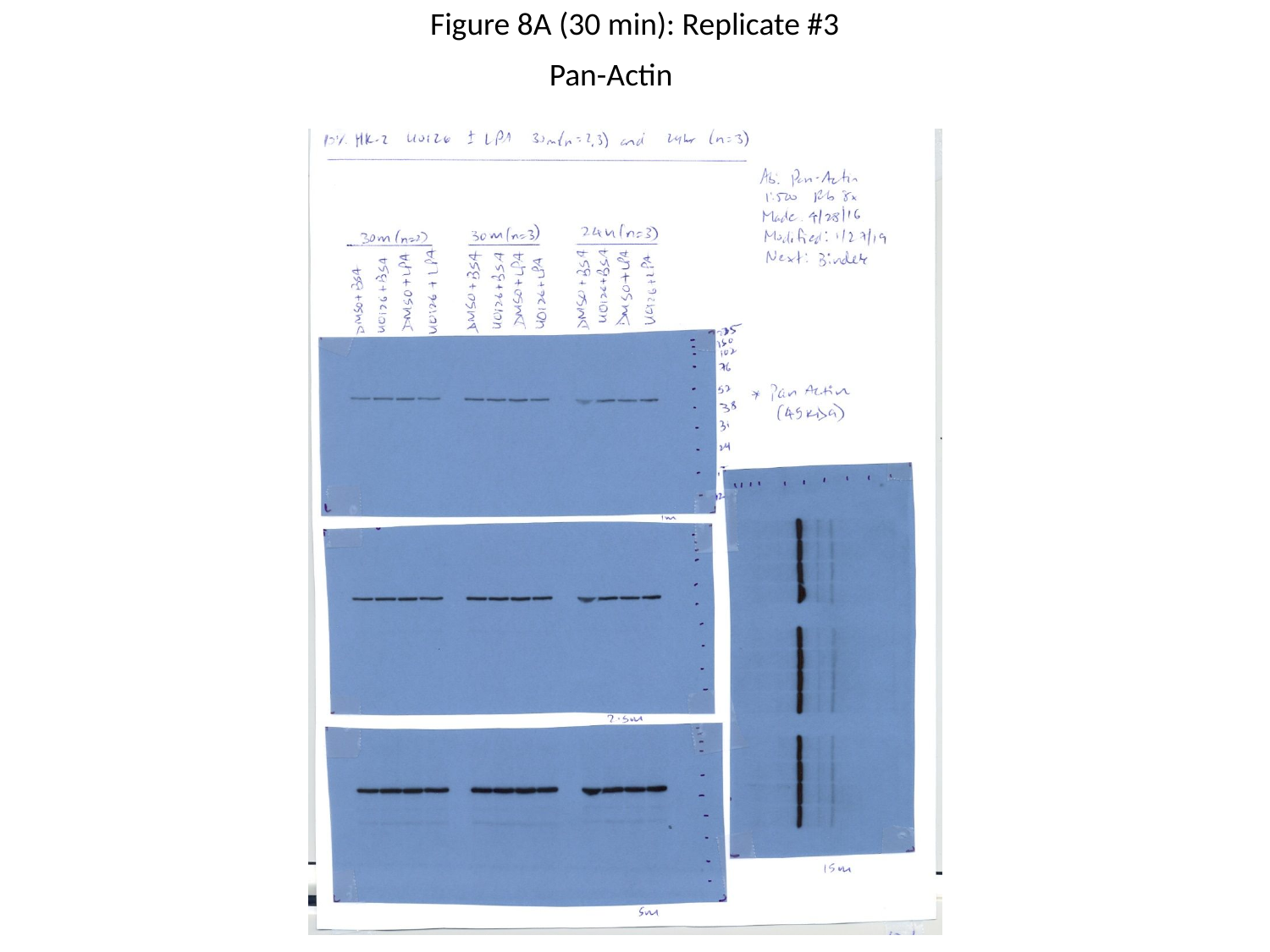

Figure 8A (30 min): Replicate #3
Pan-Actin

## Slide 39
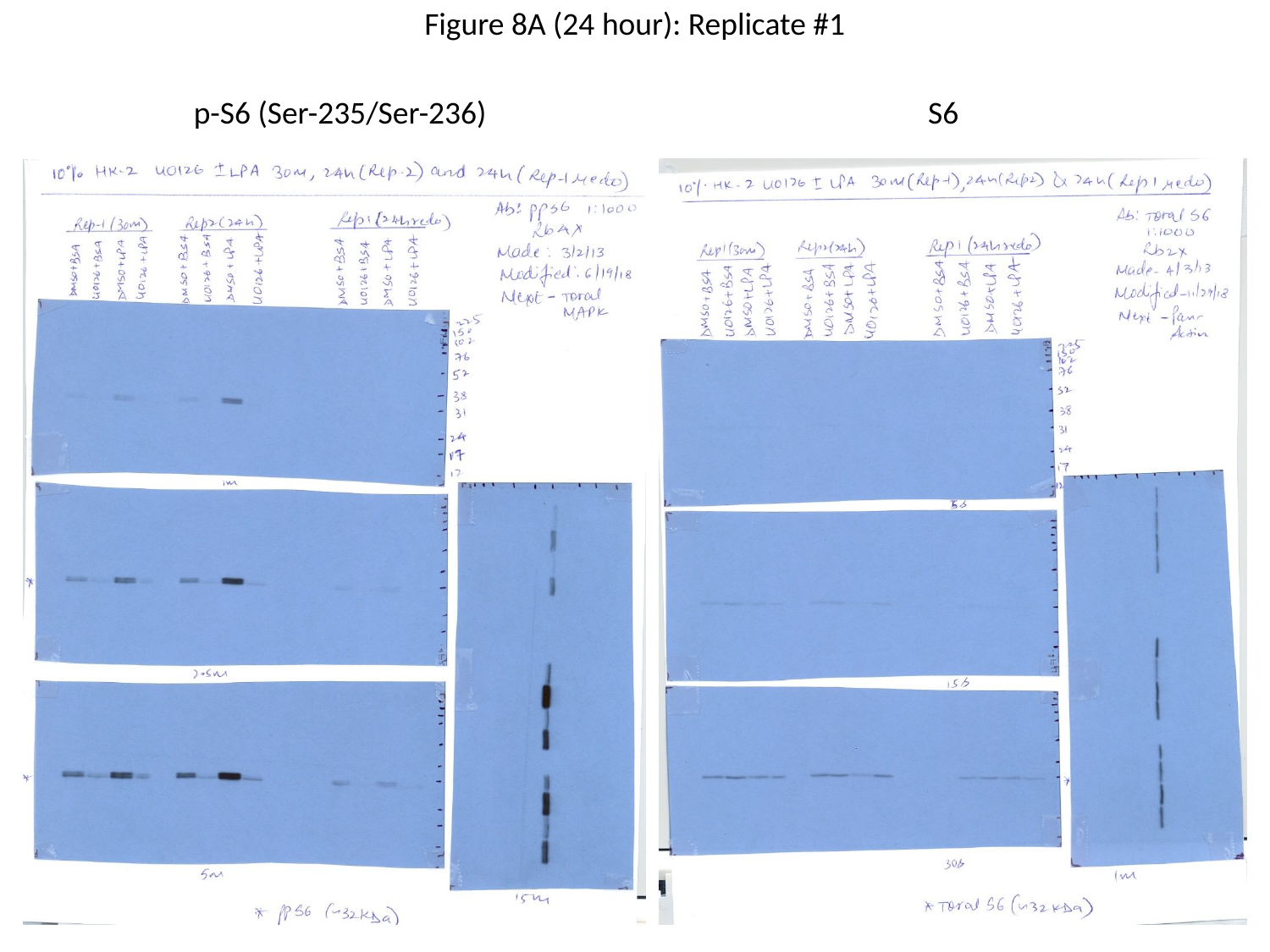

Figure 8A (24 hour): Replicate #1
p-S6 (Ser-235/Ser-236)
S6

## Slide 40
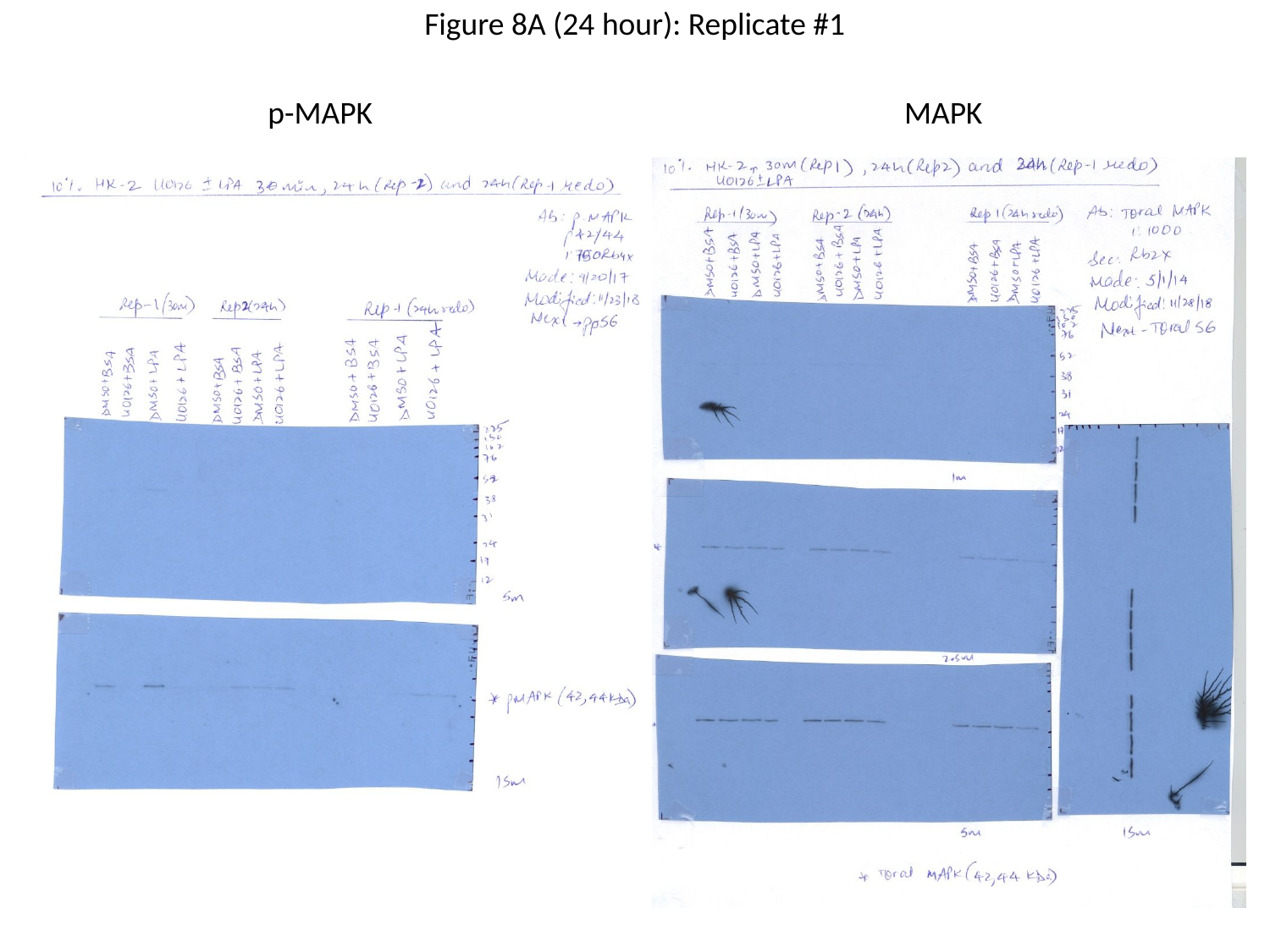

Figure 8A (24 hour): Replicate #1
p-MAPK
MAPK

## Slide 41
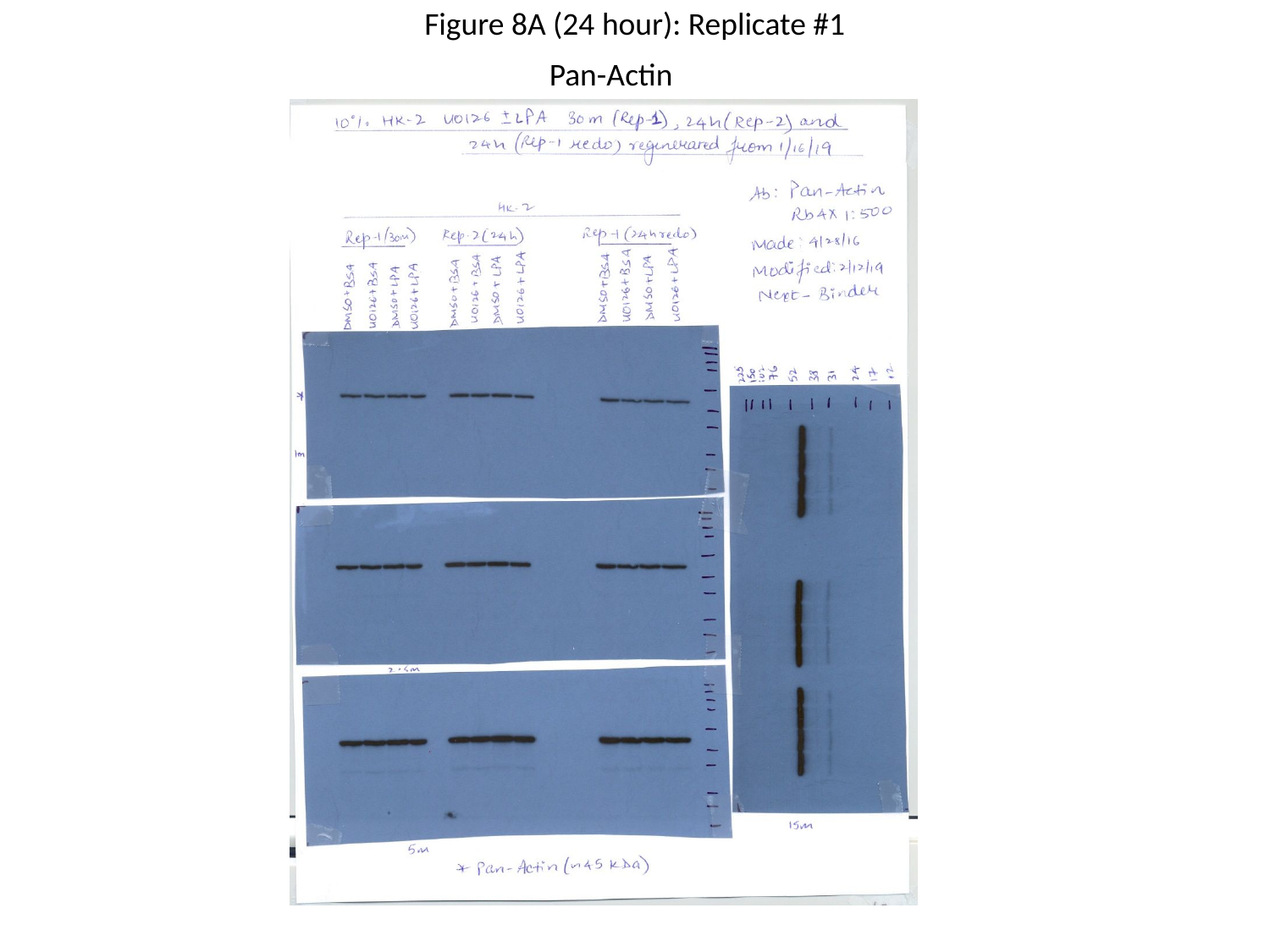

Figure 8A (24 hour): Replicate #1
Pan-Actin

## Slide 42
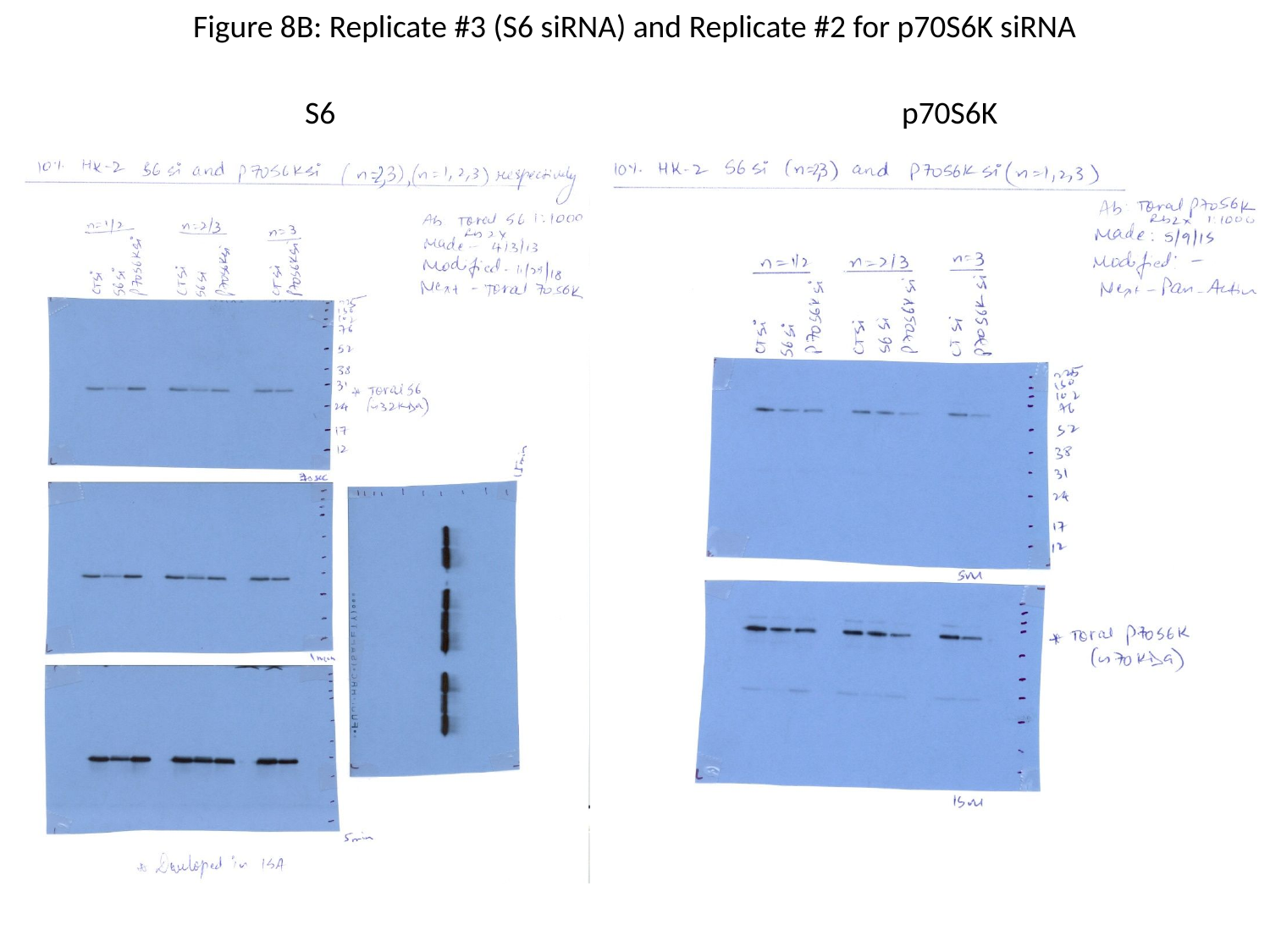

Figure 8B: Replicate #3 (S6 siRNA) and Replicate #2 for p70S6K siRNA
S6
p70S6K

## Slide 43
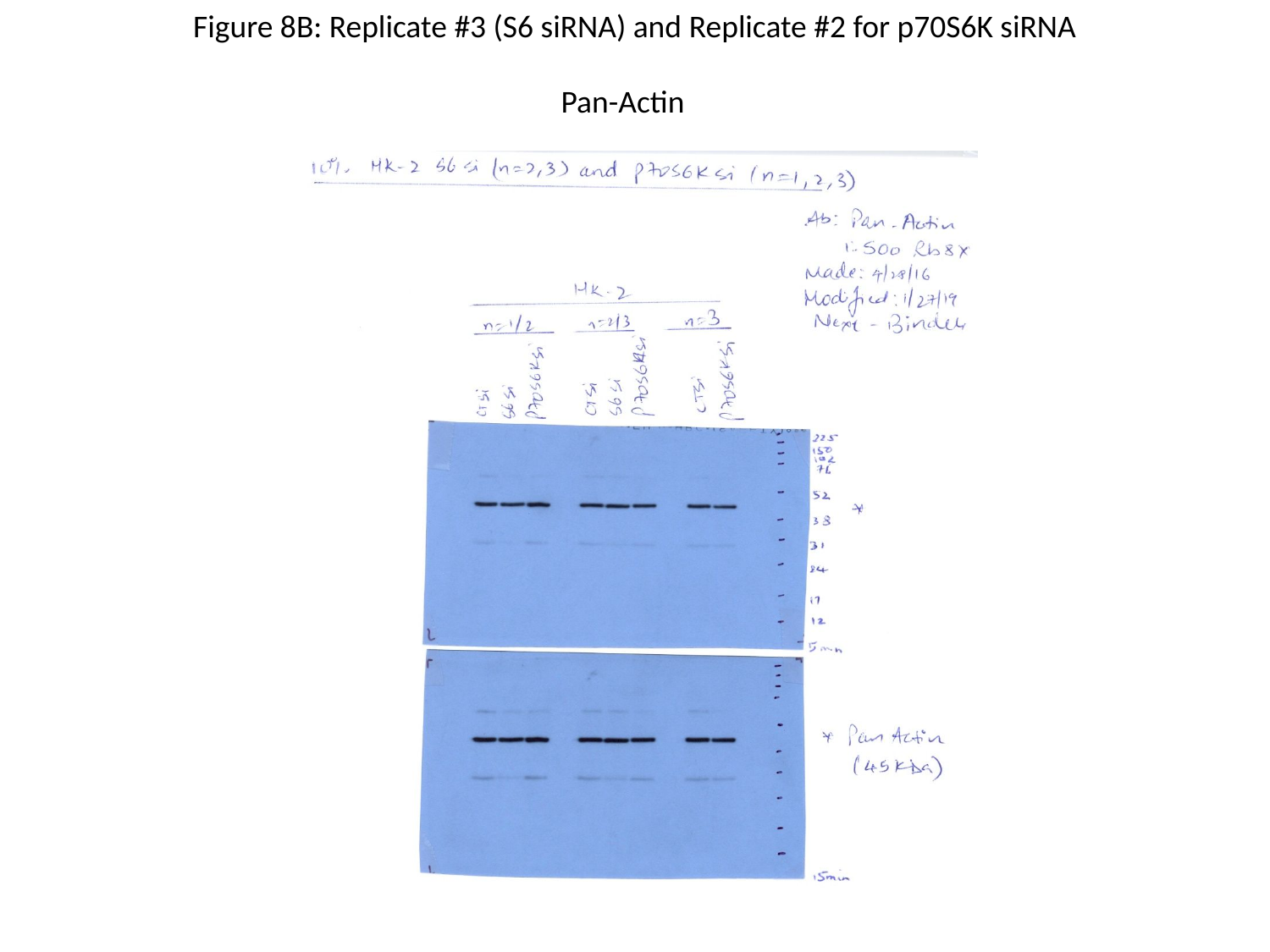

Figure 8B: Replicate #3 (S6 siRNA) and Replicate #2 for p70S6K siRNA
Pan-Actin
